# G-quadruplex homeostasis is a determinant of PARP inhibitor toxicity in BRCA2-deficient cells

**DOI:** 10.1038/s41467-026-75083-9

**Published:** 2026-07-03

**Authors:** Abhishek Bharadwaj Sharma, Joanna Krwawicz, Leandre Tappenden, Muhammad Khairul Ramlee, Arwa A. Abugable, Xin Zhen, Simran Khurana, Travis H. Stracker, Nicholas D. Lakin

**Affiliations:** 1 Department of Biochemistry, University of Oxford, South Parks Road, Oxford, UK; 2 Division of Cancer Biology, Council of Scientific & Industrial Research-Central Drug Research Institute, Lucknow, India; 3 Cancer and Stem Cell Biology Program, Duke-NUS Medical School, 8 College Road, Singapore, Singapore; 4 Radiation Oncology Branch, National Cancer Institute, National Institutes of Health, Bethesda, MD 20892 USA

**Keywords:** DNA damage and repair, DNA replication

## Abstract

Inhibition of PARPs is a key strategy to treat tumours with defects in homologous recombination (HR), including those with mutations in the tumour suppressor gene *BRCA2*. PARP inhibitors generate replication stress, creating a dependence on HR to repair the resulting DNA damage. However, the DNA lesions generated upon PARP inhibition that impede replication fork progression and trigger a requirement for BRCA2 in cell survival are poorly defined. Here, we demonstrate that elevated levels of G-quadruplex (G4) DNA structures is a determinant of genome instability and PARP inhibitor toxicity, while suppressing these structures results in PARP inhibitor resistance. The HUWE1-associated stress response protein HAPSTR1 and BRCA2 function in parallel pathways to PARP1/PARP2 to suppress G4 levels during S-phase. Mechanistically, PARP1/PARP2 disruption in HAPSTR1 or BRCA2-deficient cells leads to G4-replication conflicts, ssDNA gaps, replication-associated DNA damage and genome instability. HAPSTR1 turnover is regulated through HUWE1-dependent proteasome degradation. As such, HUWE1 disruption results in elevated HAPSTR1 and suppression of elevated G4 levels in BRCA2-deficient cells, resulting in PARP inhibitor resistance. Together, these data identify G4 structures as a determinant of PARP inhibitor toxicity, while the HAPSTR1/HUWE1 axis is essential to suppress these structures and confer PARP inhibitor resistance.

## Abstract (web summary)

The DNA structures underlying PARP inhibitor toxicity in BRCA2-deficient cells remain unclear. Here, the authors identify G-quadruplex structures as determinants of PARP inhibitor sensitivity and find that the BRCA2 and HAPSTR1–HUWE1 pathways suppress G4s to influence the PARP inhibitor response.

## Introduction

PARPs are a cornerstone of the DNA damage response (DDR) that catalyse ADP-ribosylation (ADPr) of target proteins in response to genotoxic stress. Whilst PARP1 and PARP2 are best characterised in signalling and repair of DNA single strand breaks (SSBs)^1,2^, they also regulate other strand break repair mechanisms, including processing of stalled and/or damaged replication forks. Targeting these pathways through chemical or genetic disruption of PARP1/PARP2 is toxic to homologous recombination (HR) deficient cells, and PARP inhibitors are used to treat HR-defective tumours, such as breast and ovarian cancers with mutations in *BRCA1* or *BRCA2*^3–5^. However, the mechanisms that underpin the synthetic lethal interaction between PARP1/PARP2 disruption and BRCA-deficiency, including the DNA lesions induced by PARP1/PARP2 dysfunction, are poorly understood.

The current view of PARP inhibitor toxicity in HR-deficient cells is that unresolved SSBs, or PARP1/PARP2 trapped at these sites, leads to replication fork stalling and/or collapse that requires HR-mediated repair^5,6^. More recently, however, PARP inhibitors were found to induce single-stranded DNA (ssDNA) gaps in BRCA-deficient cells and an inability to repair these structures to facilitate nascent strand maturation was proposed to be toxic to these cells^7^. The situation is further complicated by recent findings that DNA-RNA hybrids (R-loops) generated when replication forks collide with ongoing transcription also contribute to PARP inhibitor toxicity^8,9^. Although these models present a complex view of the relationship between PARP1/PARP2 and HR-mediated DNA repair, a unifying theme is that PARP1/PARP2 disruption hinders DNA replication, creating a critical need for HR to alleviate the associated replication stress. Nevertheless, the endogenous DNA lesions induced upon PARP1/PARP2 disruption that induce replication stress and a requirement for HR are poorly defined.

Factors that can stall the replisome include DNA damage, protein-DNA complexes and collision of replication forks with sites of ongoing transcription^10^. In addition, non-canonical DNA structures can also impede DNA replication. For example, G-quadruplexes (G4s) form at guanine-rich regions of the genome through Hoogsteen hydrogen bonds between DNA strands^11^. G4s are particularly prevalent in key genomic regions, including telomeres, gene promoters, and replication origins, and recent computational^12^ and genome-wide mapping techniques^13,14^ predict thousands of G4-forming motifs across the genome. G4s have been implicated in a variety of biological processes, including transcriptional regulation^15^, epigenetic programming, telomere maintenance, replication initiation and antibody maturation^11,15^. However, these structures can also act as potent replication barriers, requiring resolution by a number of factors, including the helicases Pif1, BLM, WRN, FANCJ and the DNA translocase HLTF^11,15^. Importantly, disrupting these processes results in replication fork breakage that is associated with genome instability and malignancy^11,16,17^. Indeed, defects in BRCA1 and BRCA2 have been implicated in G4 regulation to maintain genome stability^18,19^. In addition, G4s regulate R-loops, structures that contribute to PARP inhibitor toxicity in HR-defective cells^9^. Genome-wide profiling reveals R-loops form preferentially in regions enriched for G4 motifs^20,21^ and G4 stabilisation promotes R-loop accumulation in proximity to G4 sites^16^. Conversely, R-loops enhance G4 formation^22–24^, and R-loops formed at G4 structures (termed G-loops) are critical intermediates in G4 formation and resolution^25^. Taken together, these observations highlight a reciprocal relationship between R-loops and G4s. However, despite these mechanistic links, the precise contribution of R-loop/G4 structures to PARP inhibitor toxicity in HR-deficient cells remains to be determined.

Recently, we discovered that the HUWE1-associated stress response protein HAPSTR1 (also known as C16orf72/TAPR1) is synthetic lethal with PARP1/PARP2 disruption^8^. HAPSTR1 is a small, evolutionarily conserved protein identified in genetic and in silco screens as a factor involved in telomere maintenance^26^ and the cellular stress response^27,28^, including replication stress tolerance^29^. HAPSTR1 interacts with the E3 ubiquitin ligase HUWE1, serving both as a ubiquitination substrate and as a modulator of HUWE1 substrate specificity towards nuclear proteins^27,28,30^. Our recent work demonstrated that whilst HAPSTR1 is dispensable for tolerance of cells to DNA double-strand breaks (DSBs) and MMS-induced base damage, it is required to mitigate replication stress by suppressing replication-associated R-loop accumulation, maintain genome stability, and confer resistance to PARP inhibitors^8^. Here, we demonstrate that HAPSTR1, PARP1 and BRCA2 function in parallel pathways to suppress G4 levels, particularly during S-phase, and that loss of these pathways results in elevated G4s and PARP inhibitor toxicity in cells. Importantly, we also identify that disruption of HUWE1-dependent turnover of HAPSTR1 rescues the elevated G4 accumulation in BRCA2-deficient cells, resulting in PARP inhibitor resistance. Together, these data identify G4s as a lesion that contributes to PARP inhibitor sensitivity and the HAPSTR1/HUWE1 axis as a potential target to overcome PARP inhibitor resistance in BRCA2-deficient cells.

## Results

### HAPSTR1 regulates G-quadruplexes to suppress replication-associated DNA damage

**Fig. 1:**
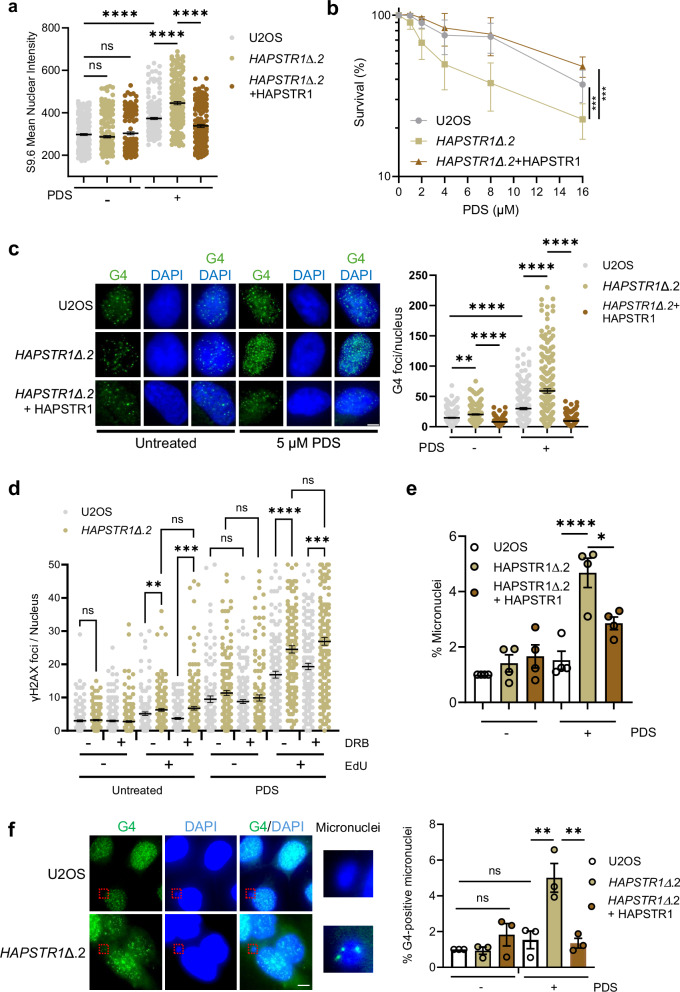
HAPSTR1 maintains G-quadruplex homoeostasis and genome stability. **a** Parental U2OS, *HAPSTR1*Δ.2 cells or *HAPSTR1*Δ.2 cells expressing Flag-HA-HAPSTR1 (*HAPSTR1Δ.2* + HAPSTR1) were treated with 5 µM PDS and R-loop levels assessed immuno-florescence with the S9.6 antibody. Mean values +/− SEM from 3 independent experiments. At least 189 cells were examined per condition. **b** Clonogenic survival assay of U2OS, *HAPSTR1Δ.2* and *HAPSTR1Δ.2* + HAPSTR1 following exposure to increasing concentrations of PDS. Data represent mean values ± SEM of 3 biological independent experiments. **c** Parental U2OS, *HAPSTR1*Δ.2 cells or *HAPSTR1*Δ.2 cells expressing Flag-HA-HAPSTR1 (*HAPSTR1Δ.2* + HAPSTR1) were treated with 5 µM PDS and nuclear G4 levels assessed by immuno-florescence. Mean values +/− SEM from 3 independent experiments, with at least 161 cells/condition, are represented. **d** U2OS and HAPSTR1Δ.2 cells were incubated −/+ 50 µM PDS and −/+ 100 µM DRB for 3 hours as indicated and γH2AX foci were counted via immunofluorescence microscopy. Cells were also pulsed with EdU to mark cells undergoing DNA replication at the time of treatment. Mean values +/− SEM from 3 independent experiments, with at least 307 cells examined per treatment condition, are represented. **e** DAPI-stained micronuclei were scored from U2OS, *HAPSTR1Δ.2 and HAPSTR1Δ.2* + HAPSTR1 cells exposed to 5 µM PDS. Mean values +/− SEM from 4 independent experiments, with at least 281 cells examined per condition over four independent biological experiments, are represented. Error bars are the +/-SEM. **f** G4 positive micronuclei were scored from cells treated as in (**e**). Representative images are presented (left hand panel). Scale bar is 5 µM. Data represent the normalised number of G4 positive micronuclei per 100 cells, with at least 256 cells/condition examined over the 3 independent biological experiments. Statistical significance was assessed by Ordinary one-way ANOVA or Kruskal-Wallis non-parametric test. Clonogenic survival assays were assessed by two-way ANOVA (*p  ≤  0.05, **p  ≤  0.01, ***p  ≤  0.001, ****p  ≤  0.0001 and ns = not significant). Source data are provided as a Source Data file.

Previously, we identified that HAPSTR1 acts in a pathway parallel to PARP1/PARP2 to supress R-loop accumulation in response to replication stress and PARP inhibitors^8^. R-loops are dynamic structures that form at various regions of the genome, including those that harbour G4 structures^11^. Therefore, we considered whether HAPSTR1 participates in G4 regulation and if the disruption of this mechanism is the cause of R-loop accumulation in *HAPSTR1* KO cells. To explore this possibility, we tested whether stabilising G4 structures with the G4 ligand pyridostatin (PDS) elevated R-loops and whether this was deregulated in the absence of HAPSTR1. Consistent with R-loops only being induced in the presence of replication stress or PARP inhibition in *HAPSTR1* KO cells^8^, no significant increase in R-loop accumulation was apparent in untreated *HAPSTR1* KO cells relative to parental U2OS cells (Fig. 1a and Supplementary Fig. 1a). However, PDS increased R-loop accumulation in parental U2OS cells, and this was further elevated in *HAPSTR1* KO cells. Importantly, the elevated levels of R-loops in *HAPSTR1* KO cells were rescued by expression of recombinant FLAG-HA-HAPSTR1. Together, these data indicate a role for HAPSTR1 in suppressing R-loop accumulation in response to G4 stabilisation.

Next, we tested the role of HAPSTR1 in regulating G4 structures. *HAPSTR1* KO cells were sensitive to PDS, and this was rescued by expression of recombinant FLAG-HA-HAPSTR1 (Fig. 1b), indicating a role for HAPSTR1 in allowing cells to tolerate G4 accumulation. To test this further, we quantified G4 levels in nuclei by indirect immunofluorescence using an antibody that recognises G4 structures (Fig. 1c). G4 structures were significantly increased in U2OS nuclei exposed to PDS and elevated by a further 2-fold in *HAPSTR1* KO cells (Fig. 1c). This phenotype was rescued by expression of recombinant FLAG-HA-HAPSTR1, confirming that suppression of G4 levels depends on HAPSTR1. A similar increase in G4 levels was observed in *HAPSTR1* KO cells when probing U2OS DNA extracts with G4-specific antibodies, in addition to G4 foci in RPE1 cells (Supplementary Fig. 1b, c), illustrating the role for HAPSTR1 in limiting the formation or stability of G4 structures is not cell type-specific. G4s and R-loops form co-transcriptionally, and in addition to G4s promoting R-loop formation, R-loops can also stabilise G4s^16,22–24,31^. Importantly, although G4 levels were significantly increased in untreated *HAPSTR1* KO cells, a corresponding increase in R-loops was not observed in the absence of PDS, even in cells undergoing DNA replication (Fig. 1a, c and Supplementary Fig. 1d), arguing these structures occur independently of R-loops in these cells under basal conditions. In addition, whilst PDS-induced R-loops were sensitive to the RNA polymerase II inhibitor 5,6-dichlorobenzimidazole 1-β-D-ribofuranoside (DRB; Supplementary Fig. 1e), the elevated G4 levels observed in PDS-treated *HAPSTR1* KO cells were unchanged (Supplementary Fig. 2a, b), indicating that R-loop formation does not contribute to G4 induction under these conditions, despite the robust accumulation of R-loops in these cells (Fig. 1a). Together, these data argue that the elevated levels of G4 structures observed in *HAPSTR1* KO cells are independent of transcription and are not a consequence of R-loop formation.

Given that G4 structures can block replication forks to promote genome instability^11,16,17^, we next assessed the consequences of the elevated G4 levels in HAPSTR1-deficient cells on replication dynamics and genome stability. *HAPSTR1* KO cells displayed increased levels of the DNA damage marker γH2AX following PDS treatment (Supplementary Fig. 2c), suggesting a role for HAPSTR1 in mitigating DNA damage induced by G4 accumulation. To determine whether the observed DNA damage was associated with DNA replication, cells were pulsed with the thymidine analogue EdU for 15 min to label S-phase cells, followed by assessment of γH2AX levels after PDS treatment. In the absence of PDS, EdU-negative *HAPSTR1* KO cells did not show a significant increase in γH2AX foci compared to parental U2OS cells (Fig. 1d). In contrast, replicating EdU-positive *HAPSTR1* KO cells displayed a marked elevation in γH2AX nuclear foci, even in the absence of PDS treatment. Consistent with no detectable R-loop induction in untreated *HAPSTR1* KO cells, this increase in γH2AX foci was unaffected by DRB treatment. Upon PDS exposure, γH2AX foci increased further in all cells, with the highest levels detected in replicating *HAPSTR1* KO cells. Following PDS treatment, a modest but non-significant reduction in γH2AX foci was observed in non-replicating cells in response to DRB, indicating only a minor contribution of R-loops to DNA damage induction in these cells. Further, DNA damage was resistant to DRB treatment in replicating cells, reinforcing the conclusion that it arises from elevated levels of G4 structures rather than R-loop formation.

To assess DSBs directly, we quantified 53BP1 foci under the same conditions. Consistent with our previous study^8^, we observed a modest but significant increase in 53BP1 nuclear bodies in untreated non-replicating cells, likely reflecting unrepaired DNA lesions carried over from the preceding S phase. As with γH2AX, elevated 53BP1 foci in untreated replicating *HAPSTR1* KO cells remained insensitive to DRB (Supplementary Fig. 2d). However, DRB treatment reduced 53BP1 foci formation in untreated non-replicating and PDS-treated *HAPSTR1* KO cells undergoing DNA replication. Importantly, DRB did not suppress 53BP1 foci formation to levels observed in U2OS cells, indicating that, like untreated cells, a proportion of DSBs in HAPSTR1 KO cells were independent of transcription. Together, these findings suggest the presence of distinct subsets of DNA damage in PDS-treated cells, with replication-associated γH2AX signalling occurring independently of transcription, whereas a proportion of subsequent replication fork breakage is transcription-dependent.

To assess the consequences of replication-associated DNA damage on genome stability, we determined the impact of HAPSTR1 loss on micronuclei formation, established markers of replication stress and genome instability^32^. Consistent with the presence of replication stress and DNA damage, micronuclei formation was elevated in *HAPSTR1* KO cells upon PDS exposure (Fig. 1e). Notably, while few G4 structures were detected in PDS-induced micronuclei in U2OS cells, *HAPSTR1* KO cells exhibited a fivefold increase in G4-containing micronuclei (Fig. 1f). In both cases, these phenotypes were rescued by the expression of HAPSTR1, confirming its role in preventing G4-associated genomic instability and micronuclei formation. Taken together, these data indicate a requirement for HAPSTR1 in supressing replication associated DNA damage to maintain genome stability in response to elevated levels of G4 structures.

### HAPSTR1 and PARP1/PARP2 function in parallel pathways to suppress G4 accumulation during DNA replication

**Fig. 2:**
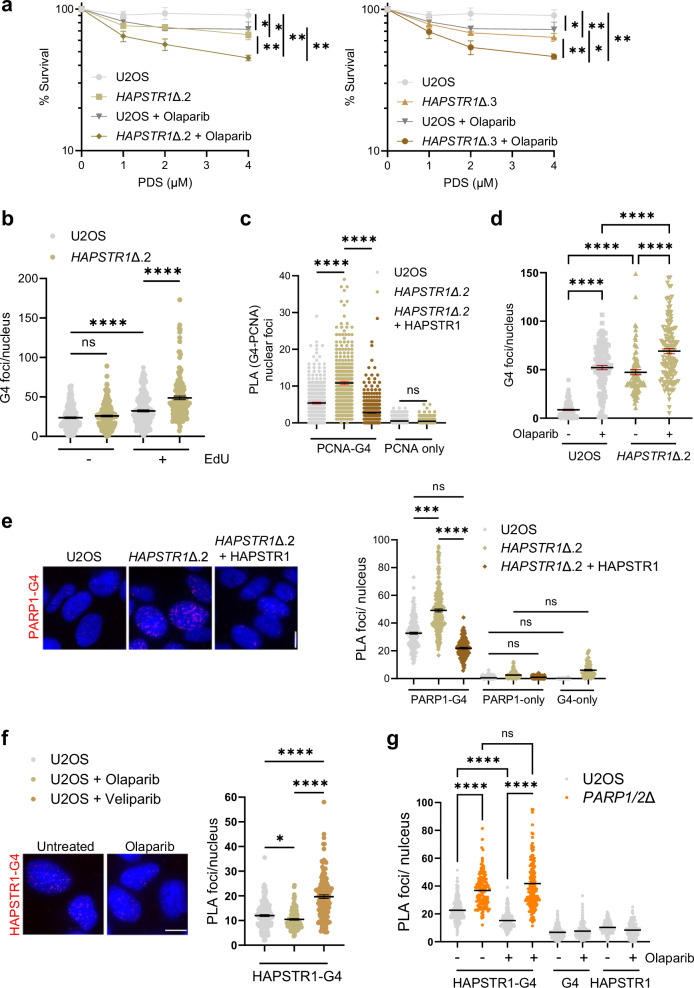
HAPSTR1 and PARP1 work in separate pathways to regulate G4s in replicating cells and prevent replication associate genome instability. **a** Survival assays of U2OS, *HAPSTR1Δ.2* and *HAPSTR1Δ.3* cells with PDS −/+ exposure to 5 µM olaparib. Data using the *HAPSTR1*Δ.2 (left) and *HAPSTR1*Δ.2 (right) were generated in parallel using the same U2OS control, but graphs for the two knockouts are plotted separately. Data represent mean values ± SEM of 4 independent experiments. **b** *HAPSTR1Δ.2* and U2OS cells were incubated with 10 µM EdU to mark replicating cells prior to assessing G4 levels by immunoflorescence. Mean values +/− SEM from 3 independent experiments, with at least 153 cells examined/condition. **c** U2OS and *HAPSTR1* KO cells were subjected to PLA using the indicated antibodies. Mean values +/− SEM from 3 independent experiments are represented, with at least 254 cells examined/condition. **d** *HAPSTR1Δ.2* and U2OS cells were incubated with 5 µM olaparib prior to assessing G4 levels by immunofluorescence. Mean values +/− SEM from 3 independent experiments, with at least 223 cells scored/condition. **e** U2OS, *HAPSTR1Δ.2* and *HAPSTR1Δ.2* + HAPSTR1 cells were subjected to proximity ligation assays using the anti-PARP1 and anti-G4 antibodies as indicated. Representative images (left) and mean values +/− SEM (right) from 3 independent experiments are represented, with at least 369 cells examined/condition. Scale bar =5 μm.** f** U2OS cells were incubated with 5 µM olaparib or 10 µM veliparib and subjected to proximity ligation assays using the anti-HAPSTR1 and anti-G4 antibodies. Representative images (left) and mean values +/− SEM (right) from 3 independent experiments are represented, with at least 267 cells/condition examined/condition. Scale bar = 5 μm.** g** U2OS and *PARP1/2Δ* cells were incubated with 5 µM olaparib and subjected to proximity ligation assays using the anti-HAPSTR1 and anti-G4 antibodies alone or in combination, as indicated. Mean values +/− SEM from 3 independent experiments are represented, with at least 310 cells scored/condition. All the statistical significance was assessed by Ordinary one-way ANOVA or Kruskal-Wallis non-parametric test. Clonogenic survival assays were assessed by two-way ANOVA (*p  ≤  0.05, **p  ≤  0.01, ***p  ≤  0.001, ****p  ≤  0.0001 and ns = not significant). Source data are provided as a Source Data file.

R-loops generated during transcription-replication conflicts are responsible for PARP inhibitor toxicity in *HAPSTR1* or *BRCA2*-deficient cells^8,9^. Combined with our findings that G4 stabilisation using PDS promotes elevated R-loop accumulation in *HAPSTR1* KO cells (Fig. 1a), this suggested a potential role for PARP1/PARP2 in the regulation of G4 structures. Consistent with this hypothesis, exposure of U2OS cells to the PARP inhibitor olaparib sensitised them to PDS (Fig. 2a). Furthermore, olaparib treatment led to an enhanced sensitivity to PDS in two independent *HAPSTR1* KO cell lines (*HAPSTR1*Δ.2 and *HAPSTR1*Δ.3; Fig. 2a)^8^, indicating that HAPSTR1 and PARP1/PARP2 act in parallel pathways to allow cells to tolerate G4 stabilisation by the G4 ligand PDS.

The synthetic lethal interaction between HAPSTR1 and PARP1/PARP2^8^ suggested a requirement for these proteins in the absence of exogenous stress. Therefore, we assessed the role of these proteins in G4 regulation when cells have not been challenged with PDS. As reported previously^33,34^ a modest but significant elevation of G4s was observed in replicating EdU- positive U2OS cells (Fig. 2b). Moreover, this increase was further elevated in EdU-positive *HAPSTR1* KO cells compared to parental U2OS, implicating HAPSTR1 in the suppression of G4 levels during unperturbed DNA replication. The pattern of elevated G4 levels in replicating *HAPSTR1* KO cells parallels the induction of DNA damage observed in the absence of PDS (Fig. 1d). Therefore, we considered whether DNA damage in these cells is a result of replication-G4 conflicts. Consistent with this hypothesis, we observe an increased proximity of the replication fork component PCNA with G4s, as judged by proximity ligation assay (PLA; Fig. 2c and Supplementary Fig. 3a). In addition, DNA fibre analysis combined with G4 co-staining revealed increased replication fork stalling at G4 sites in *HAPSTR1* KO cells (Supplementary Fig. 3b), further supporting a role for HAPSTR1 in suppressing G4 replication conflicts to maintain genome integrity during DNA replication.

Having established that elevated G4s in *HAPSTR1* KO cells occur during S-phase, we next investigated whether PARP1/PARP2 similarly suppress G4 levels. Treatment of cells with olaparib significantly increased nuclear G4 levels in U2OS cells (Fig. 2d and Supplementary Fig. 4a). A similar increase in G4 levels was observed in *PARP1/2* KO cells, specifically in cells undergoing DNA replication (Supplementary Fig. 4b), supporting the conclusion that similar to HAPSTR1, PARP1/2 suppress G4 accumulation during S-phase. Furthermore, G4 accumulation in *HAPSTR1* KO cells was further increased by olaparib treatment (Fig. 2d), suggesting that HAPSTR1 and PARP1/PARP2 operate in parallel pathways to suppress G4 formation either by restraining their formation or promoting their resolution.

Given that HAPSTR1^26^ and PARP1^35–37^ have been implicated in telomere maintenance, and that telomeres can form G4 structures in cells^38–40^, we asked whether the elevated G4 levels we observe are confined to telomeres or reflect a more general phenomenon. Co-staining of G4s in *HAPSTR1* KO cells with the telomere-specific marker TRF1 revealed increased G4 formation at both telomeric and non-telomeric regions that is further enhanced upon exposure to PARP inhibitors (Supplementary Fig. 5a). We also observed a comparable increase in G4 levels in ALT-negative *HAPSTR1* KO RPE1 cells, that like in U2OS cells, is further increased in response to PARP inhibitors (Supplementary Fig. 5b). G4s within telomeric sequences are more densely enriched than those in non-telomeric regions and may therefore be preferentially detected in our immunofluorescence experiments. Nevertheless, we still observe a substantial number of G4 foci that do not colocalise with TRF1. We therefore interpret these findings to indicate that G4 dysregulation is not restricted to telomeres and does not depend on ALT activity.

Given PARP1 is known to bind G4s in vitro and in cells^41–46^, we next tested whether HAPSTR1 disruption enhanced PARP1 association with G4s in cells. Proximity ligation assays using G4 and PARP1-specific antibodies revealed that PARP1 localises in close proximity to G4 structures in U2OS cells and that this is further enhanced in the absence of HAPSTR1 (Fig. 2e). Consistent with the elevated G4 levels observed in PDS-treated cells, the number of PARP1–G4 PLA signals increased following PDS treatment (Supplementary Fig. 5c), further supporting the hypothesis that loss of HAPSTR1 channels G4 regulation through a PARP1-dependent mechanism. However, PARP inhibition with olaparib reduced HAPSTR1-G4 proximity (Fig. 2f). This was unexpected, as HAPSTR1 and PARP1/PARP2 function in redundant or compensatory pathways, inhibition of PARP activity would be expected to enhance, rather than diminish, HAPSTR1 association with G4s. However, olaparib traps PARP1/PARP2 at DNA lesions^6^, which may increase their retention at G4 structures, thereby obstructing the recruitment of HAPSTR1. Consistent with this model, exposure of cells to veliparib, a PARP inhibitor with lower trapping activity than olaparib^47^, induced an increase in the proximity of HAPSTR1 with G4 structures (Fig. 2f). Similarly, enhanced HAPSTR1-G4 association was observed in *PARP1/2* double KO cells (Fig. 2g). Notably, *PARP1/2* knockout restored HAPSTR1-G4 proximity in cells treated with olaparib, further indicating that PARP1 trapping impedes HAPSTR1 access to G4 structures. Together, these findings support a model in which HAPSTR1 and PARP1/PARP2 function in parallel pathways to recognise and suppress G4 accumulation during DNA replication.

### HAPSTR1 and PARP1/PARP2 function in parallel pathways to suppress ssDNA gaps at G4 structures

**Fig. 3:**
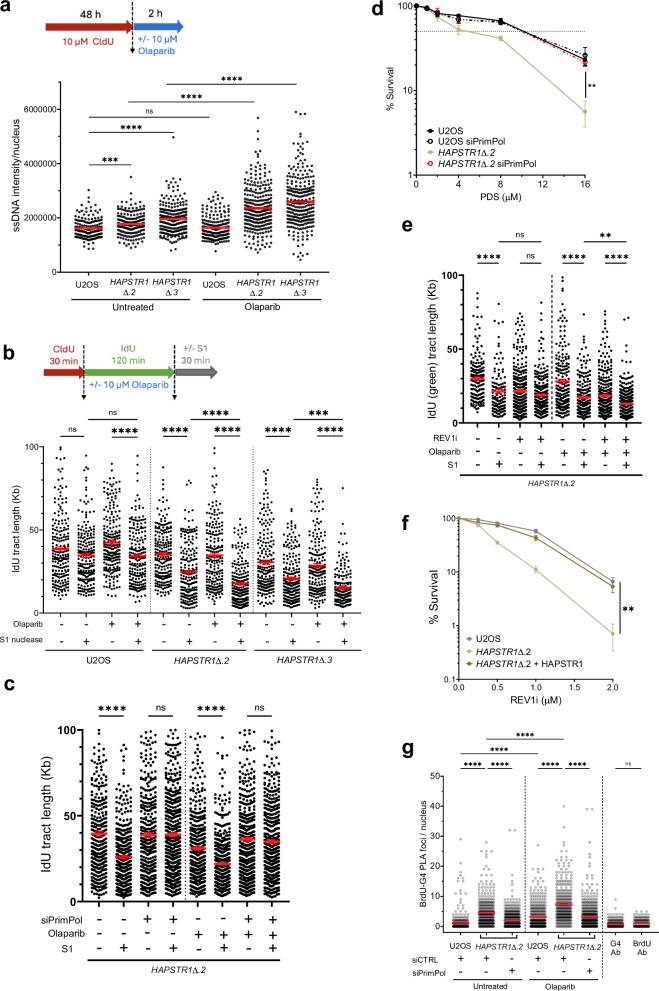
HAPSTR1 and PARP1/PARP2 suppress ssDNA gaps formation at G4 structures. **a** U2OS, *HAPSTR1Δ.2* or *HAPSTR1Δ.3* cells were treated as indicated (top) and ssDNA quantified by immunofluorescence using anti-CldU antibodies. Data represent mean values ± SEM of 3 biological independent experiments, with at least 450 cells scored/condition. **b** U2OS, *HAPSTR1Δ.2* or *HAPSTR1Δ.3* cells were treated as indicated (top). Data represent mean values ± SEM of 3 biological independent experiments, with at least 186 DNA fibres scored/condition. **c** *HAPSTR1Δ.2* cells transfected with either control or PRIMPOL siRNA were treated as in (b). Data represent mean values ± SEM of 3 biological independent experiments, with at least 317 DNA fibres scored/condition. **d** U2OS and *HAPSTR1Δ.2* cells were transfected with control or PRIMPOL siRNA as indicated and subjected to clonogenic survival assay following exposure to PDS. Data represent mean values ± SEM of 3 independent biological experiments. **e** *HAPSTR1Δ.2* cells were pulsed with CldU, and then IdU (−/+ 10 μM olaparib and REV1i), followed by S1 nuclease digestion as indicated. Data represent mean values ± SEM of 3 biological independent experiments, with at least 169 DNA fibres scored/condition. **f** Clonogenic survival assay of U2OS, *HAPSTR1Δ.2* and *HAPSTR1Δ.2* + HAPSTR1 following exposure to the REV1 inhibitor JH-RE-06. Data represent mean values ± SEM of 3 biological independent experiments. **g** U2OS cells or *HAPSTR1Δ.2* cells transfected with siControl or siPRIMPOL were labelled with CldU for 48 h and then subjected to a PLA using the indicated antibodies. Data represent mean values ± SEM of 3 independent biological experiments, with at least 405 cells scored/condition. For figures (**a**–**c**, **e** and **g**), data from 3 independent experiments were combined and the mean values ± SEM represented. Statistical significances were assessed as follows: a and b, One-way ANOVA with Brown-Forsythe and Welch ANOVA tests, and Dunnett’s multiply comparison tests on the mean of each experiment. c, e and g, One-way ANOVA with Kruskal-Wallis non-parametric test of the mean of each experiment. d and f, two-way ANOVA multiple comparison test. (*p  ≤  0.05, **p  ≤  0.01, ***p  ≤  0.001, ****p  ≤  0.0001 and ns = not significant). Source data are provided as a Source Data file.

PARP inhibitors induce accumulation of post-replicative ssDNA gaps in BRCA-deficient cells that correlate with PARP inhibitor sensitivity^7^. Therefore, we considered whether ssDNA gap formation is a characteristic of HAPSTR1 dysfunction. Using a previously reported BrdU/CIdU staining assay to detect ssDNA gap formation in cells^48^, we observed a significant increase in ssDNA gaps in *HAPSTR1* KO cells, which was further exacerbated by PARP inhibitor treatment (Fig. 3a and Supplementary Fig. 6a). To verify that ssDNA gaps are associated with ongoing DNA synthesis, we used an assay that employs S1 nuclease digestion to quantify the length of ssDNA gaps on nascent DNA strands^48^. DNA fibres from *HAPSTR1* KO cells were more readily digested by S1 nuclease, indicating the presence of ssDNA gaps (Fig. 3b and Supplementary Fig. 6b). S1 cleaved tract length was further decreased upon olaparib treatment in *HAPSTR1* KO cells, indicating that HAPSTR1 and PARP1/PARP2 work in separate pathways to suppress post-replicative ssDNA gaps.

Accumulation of ssDNA gaps in BRCA-deficient cells is initiated by repriming of replication forks using the DNA primase-polymerase PRIMPOL^49–51^ and subsequent repair by the translesion synthesis (TLS) polymerase complex REV1-Polζ^50,52^. Therefore, we tested whether the ssDNA gaps observed in *HAPSTR1* KO cells were the result of PRIMPOL or REV1-Polζ activities. The increase in ssDNA gaps observed in olaparib treated *HAPSTR1* KO cells were suppressed upon PRIMPOL depletion (Fig. 3c and Supplementary Fig. 6c), suggesting a requirement for PRIMPOL to generate these structures. In addition, PRIMPOL depletion reduced cellular sensitivity to PDS (Fig. 3d), indicating that the toxicity associated with G4 stabilisation arises, at least in part, through PRIMPOL-dependent events. In contrast to PRIMPOL, exposure of cells to the REV1-Polζ inhibitor JH-RE-06 exacerbated ssDNA gap formation in olaparib treated *HAPSTR1* KO cells (Fig. 3e and Supplementary Fig. 6d), including an overall decrease in DNA fibre length, suggesting replication fork degradation and a requirement for TLS activity for proper processing of these gaps. In addition, *HAPSTR1* KO cells were more sensitive to JH-RE-06 than parental U2OS cells, and this was rescued by expression of recombinant FLAG-HA-HAPSTR1 (Fig. 3f), further supporting the requirement for TLS in the processing ssDNA gaps in *HAPSTR1* KO cells.

Given the role of HAPSTR1 and PARP1/PARP2 in G4 regulation, we next investigated whether G4 structures contributed directly to the formation of ssDNA gaps. Cells were incubated with CldU for 48 hours to enable detection of exposed CldU epitopes on regions of ssDNA. PLAs were then employed to assess the spatial association between G4 structures and single-stranded DNA following PARP inhibition in U2OS or *HAPSTR1* KO cells. Both *HAPSTR1* KO cells or U2OS cells treated with PARP inhibitors exhibited increased CldU–G4 proximity (Fig. 3g), indicating that ssDNA regions frequently form at G4s under these conditions. Furthermore, PARP inhibition resulted in a six-fold increase in CldU–G4 proximity in *HAPSTR1* KO cells, consistent with HAPSTR1 and PARP1/PARP2 functioning in parallel pathways to prevent ssDNA formation at G4 sites. Importantly, the increased in PLA signal in *HAPSTR1* KO cells was suppressed by PRIMPOL depletion, indicating that the observed single-stranded regions correspond to PRIMPOL-dependent ssDNA gaps rather than ssDNA generated by other mechanisms. Overall, these data identify PARP1/PARP2 and HAPSTR1 function in distinct pathways to suppress G4 replication conflicts resulting in ssDNA gaps and PRIMPOL/TLS-dependent repair.

### HAPSTR1 and BRCA2 act in parallel pathways to promote PARP inhibitor resistance

Given the association between PARP inhibitor sensitivity, G4 formation, and ssDNA gap formation, we next examined whether this relationship extends to other synthetic lethal interactions involving PARP inhibition. Similar to *HAPSTR1* KO cells, G4 stabilising ligands generate replication-associated DNA damage that is toxic to BRCA2-deficient cells^18,19^. Therefore, we sought to determine whether BRCA2 is required to suppress G4 accumulation and if so, how this function intersects with HAPSTR1 and PARP inhibitor resistance.

**Fig. 4:**
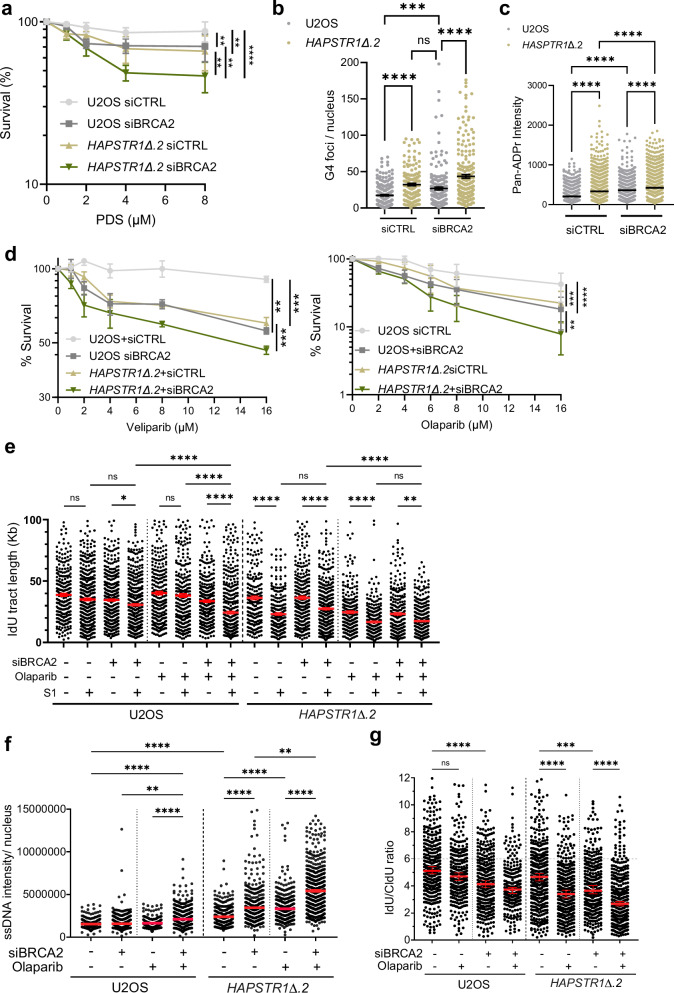
HAPSTR1 and BRCA2 function in parallel pathways to suppress G4 structures and promote PARP inhibitor resistance. **a** Survival assays of U2OS and *HAPSTR1Δ.2* cells transfected with the indicated siRNA and treated with PDS. Data represent mean values ± SEM of 3 biological independent experiments. **b** U2OS and *HAPSTR1Δ.2* cells were transfected with indicated siRNA and G4 foci assessed by immunofluorescence. Mean values ± SEM from 3 independent experiments, with at least 166 cells/condition. **c** U2OS and *HAPSTR1Δ.2* cells transfected with the indicated siRNA were assessed for pan-ADPr by immunofluorescence. Mean values ± SEM from 4 independent experiments are represented, with examination of at least 2130 cells/condition. **d** Survival assays of U2OS and *HAPSTR1Δ.2* cells transfected with indicated siRNA and treated with olaparib or veliparib. Data represent mean values ± SEM of 3 independent biological experiments. **e** *HAPSTR1Δ.2* cells transfected with the indicated siRNAs were pulsed with CldU, then IdU (-/+ olaparib), followed by S1 nuclease digestion as indicated. Data represent mean values ± SEM of 3 independent biological experiments, with at least 220 DNA fibres scored/condition. **f** U2OS and *HAPSTR1Δ.2* cells were transfected with the indicated siRNA and following incubation with CldU for 48 h and exposed to 10 µM olaparib. ssDNA was quantified anti-BrdU immunofluorescence. Data represent mean values ± SEM from three independent experiments, with at least 251 cells quantified/condition. **g** U2OS and *HAPSTR1*Δ.2 cells were transfected with BRCA2 siRNA, pulsed with CldU and then treated with IdU for 180 min −/+ the addition of 10 µM olaparib. Data represent mean values ± SEM of 3 independent biological experiments, with at least 256 DNA fibres scored/condition. Statistical significances were assessed as follows: a and d, two-way ANOVA multiple comparison test. b and f, One-way ANOVA with Brown-Forsythe and Welch ANOVA tests, and Dunnett’s multiply comparison tests on the mean of each experiment. **c** Ordinary one-way ANOVA with Tukey’s multiple comparison test. **e**, **g** One-way ANOVA with Brown-Forsythe and Welch ANOVA tests, and Dunnett’s multiply comparison tests on the mean of each experiment. (*p  ≤  0.05, **p  ≤  0.01, ***p  ≤  0.001, ****p  ≤  0.0001, ns = not significant). Source data are provided as a Source Data file.

Consistent with a role for BRCA2 in G4 homoeostasis, BRCA2 depletion led to a subtle but significant sensitisation of cells to the G4-stabilising ligand PDS to a similar extent as HAPSTR1 loss (Fig. 4a). Moreover, depletion of BRCA2 in *HAPSTR1* KO U2OS cells led to an additional increase in PDS sensitivity, indicating these two proteins function in parallel pathways to tolerate G4 stabilisation. BRCA2 depletion in U2OS cells resulted in elevated G4 structures, whilst depletion of BRCA2 in a *HAPSTR1* KO background led to an additional rise in G4 levels, supporting the conclusion that BRCA2 and HAPSTR1 act in parallel pathways to suppress G4 accumulation (Fig. 4b and Supplementary Fig. 7a). We also observed a comparable additive increase in G4 levels in *HAPSTR1* KO RPE1 cells upon depletion of BRCA2, indicating this phenotype is not restricted to U2OS cells and is independent of ALT status (Supplementary Fig. 7b). Given the role of BRCA2 in regulating G4s at telomeres^25,40,53^, we also examined whether the increased G4 accumulation was confined to these structures. However, co-staining of G4s with the telomere marker TRF1 revealed elevated G4s at both telomeric and non-telomeric regions upon BRCA2 depletion (Supplementary Fig. 7c), indicating a general increase in G4 levels. Intriguingly, the elevated G4 levels observed in *HAPSTR1* KO cells with BRCA2 depletion was restricted to non-telomeric regions, indicating that the additional G4 accumulation occurs outside of telomeres.

Having demonstrated that HAPSTR1 and BRCA2 function in parallel pathways to suppress G4 accumulation, we next considered whether disruption of these pathways in combination would further enhance the sensitivity of cells to PARP inhibitors. To this end, we evaluated whether loss of HAPSTR1 and BRCA2 enhances dependence on PARP1/PARP2 by measuring ADPr levels in cells following disruption of these genes alone, or in combination. *HAPSTR1* KO, or depletion of BRCA2 using siRNA, elevated ADPr levels in cells (Fig. 4c). Moreover, BRCA2 depletion in *HAPSTR1* KO cells resulted in a further increase in ADPr, indicating these genes act in parallel pathways to suppress G4 accumulation and PARP1/PARP2 activation. Consistent with a reliance on PARP1/PARP2 when BRCA2 and HAPSTR1 are dysfunctional, disruption of BRCA2 or HAPSTR1 sensitised cells to two independent PARP inhibitors and this was further increased when these genes are disrupted in combination (Fig. 4d). We also observed a sensitivity of *HAPSTR1* KO RPE1 cells to PARPi that is further enhanced upon depletion of BRCA2 (Supplementary Fig. 7d), indicating this phenotype is not restricted to U2OS cells. Consistent with previous reports^48,54^, and similar to *HAPSTR1* KO cells (Fig. 3b), we observe elevated ssDNA gap formation in BRCA2 depleted cells that is further increased upon exposure to PARP inhibitors (Fig. 4e and Supplementary Fig. 6e). However, no further increase in olaparib-induced ssDNA gap formation was apparent when HAPSTR1 and BRCA2 were disrupted in combination, suggesting that these proteins function in the same pathway to supress ssDNA gap formation. Given that G4s form at PRIMPOL-dependent ssDNA regions in *HAPSTR1* KO cells (Fig. 3c, g), this demonstrates a proportion of BRCA2-induced ssDNA gaps also form at G4s. However, BRCA2 depletion in *HAPSTR1* KO cells further increased G4 levels (Fig. 4b and Supplementary Fig. 7a, b), arguing that these genes function in parallel pathways in terms of suppressing nuclear G4 levels. Together, these observations suggest two populations of G4 structures: one in which BRCA2 and HAPSTR1 function cooperatively to suppress ssDNA gap formation, and a second population in which G4 regulation by HAPSTR1 and BRCA2 occurs independently.

The finding that olaparib-induced ssDNA gaps was not further increased when BRCA2 and HAPSTR1 are disrupted in combination was unexpected, given the increased olaparib sensitivity of these cells relative to either perturbation alone (Fig. 4d). However, the reduced fibre length in the absence of S1 nuclease in these cells suggested to us that loss of BRCA2 and HAPSTR1 in combination might affect replication fork stability. Consistent with this, a marked increase in olaparib-induced ssDNA was observed in *HAPSTR1* KO cells or following BRCA2 depletion, that was increased further when these genes are disrupted in combination (Fig. 4f). To test whether this is attributable to replication fork degradation, we performed DNA fibre assays to directly assess the stability of nascent DNA strands following PARP inhibition. Similar to ssDNA formation, while depletion of BRCA2 or PARP inhibition resulted in a modest but significant fork degradation in *HAPSTR1* KO cells, this was further exacerbated when siBRCA2 and PARP inhibitions were performed in combination (Fig. 4g). Taken together, these data indicate that HAPSTR1, BRCA2, and PARP1/PARP2 operate through distinct pathways to regulate G4 levels and that the toxicity of PARP inhibitors in the absence of BRCA2 and HAPSTR1 is associated with replication fork instability.

### Disruption of HUWE1-dependent HAPSTR1 turnover leads to PARP inhibitor resistance in BRCA2 disrupted cells

**Fig. 5:**
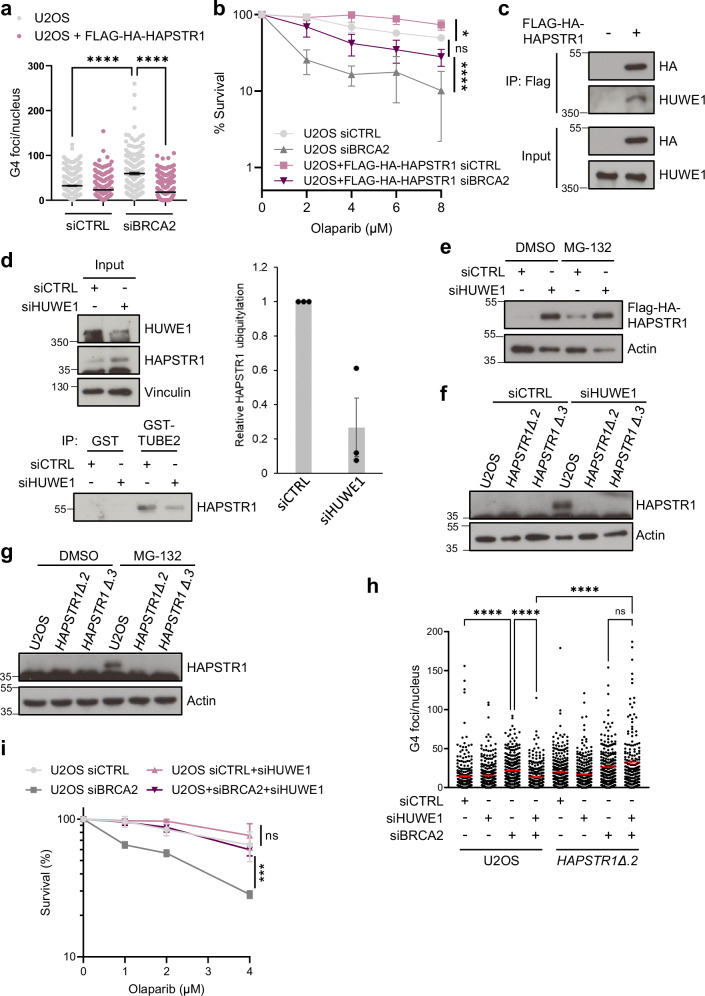
Disrupting HUWE1-dependent HAPSTR1 turnover leads to suppression of G4s and PARP inhibitor resistance. **a** U2OS, *HAPSTR1*Δ.2 cells or *HAPSTR1*Δ.2 cells expressing Flag-HA-HAPSTR1 (*HAPSTR1Δ.2* + HAPSTR1) were transfected with indicated siRNA and nuclear G4 foci assessed by immunofluorescence. Mean values ± SEM from 3 independent experiments, with at least 202 cells/condition, are represented. **b** Clonogenic survival assay of U2OS cells with or without Flag-HA-HAPSTR1 expression, transfected with the indicated siRNA and exposed to increasing concentrations of olaparib. Data represent mean values ± SEM of 3 independent biological experiments. **c** Whole cell extracts were prepared from cells, and Flag-HA-HAPSTR1 immunoprecipitated using an anti-Flag antibody. Western blotting on inputs or immunoprecipitates were performed using the indicated antibodies. Data is representative of 3 biological repeats. **d** Cells were transfected with the indicated siRNA, and following preparation of whole cell extracts, ubiquitylated proteins were pulled down using GST-tagged tandem ubiquitin binding entity (GST-TUBE2). HUWE1 and HAPSTR1 were detected in inputs (left panel) or pulldowns (lower panel) by western blotting. The panel on the right represents mean values of HAPSTR1 in pulldowns ± SEM relative to input samples from 3 independent experiments. **e**–**g** Western blot analysis using the indicated antibodies to assess the level of FLAG-HA-HAPSTR1 (**e**) or endogenous HAPSTR1 (**f**, **g**) in whole cell lysates prepared from cells transfected with the indicated siRNA and/or treated with 20 µM MG-132. Data are representative of 2 biological repeats. **h** U2OS and *HAPSTR1Δ.2* cells were transfected with the indicated siRNA, and nuclear G4 levels assessed by immunofluorescence. Mean values +/− SEM from 3 independent experiments, with at least 205 cells/condition, are represented. **i** Clonogenic survival assay with U2O cells transfected with the indicated siRNA and treated with increasing concentrations of olaparib. Data represent mean values ± SEM of 3 independent biological experiments. All the statistical significance was assessed by Ordinary one-way ANOVA or Kruskal-Wallis non-parametric test. Clonogenic survival assays were assessed by two-way ANOVA (*p  ≤  0.05, **p  ≤  0.01, ***p  ≤  0.001, ****p  ≤  0.0001 and ns = not significant). Source data are provided as a Source Data file.

Our data indicate that HAPSTR1 and BRCA2 function in parallel pathways to suppress G4 structures and PARP1/PARP2 activation during an unperturbed cell cycle. This led us to investigate whether increased HAPSTR1 expression could suppress the elevated G4 levels in BRCA2-deficient cells and whether this is able to confer resistance to PARP inhibitors. To address this, we expressed recombinant FLAG–HA–HAPSTR1 in U2OS cells retaining endogenous HAPSTR1, resulting in an approximate two-fold increase in total HAPSTR1 expression (Supplementary Fig. 8a, b). We then assessed G4 accumulation, ssDNA gap formation, and sensitivity to PARP inhibitors following BRCA2 depletion. Consistent with our previous results, BRCA2 depletion led to a significant increase in G4 accumulation in cells (Fig. 5a) and as expected^3,4^, PARP inhibitor sensitivity and ssDNA gap accumulation (Fig. 5b and Supplementary Fig. 8d). Interestingly, the BRCA2-dependent increase in G4 levels was completely suppressed by the expression of FLAG-HA-HAPSTR1, resulting in suppression of ssDNA gaps and resistance to PARP inhibitors (Fig. 5a, b and Supplementary Fig. 8c, d).

Next, we considered negative regulators of HAPSTR1 and how these might impact G4 homoeostasis and PARP inhibitor resistance. HAPSTR1 interacts with the E3 ubiquitin ligase HUWE1 to regulate the adaptive response to a variety of cellular stresses^27,28,55^. Co-immunoprecipitation of HUWE1 with HAPSTR1 in U2OS cells confirmed the interaction between these two proteins under our experimental conditions (Fig. 5c). We were able to purify HAPSTR1 using GST-tagged Tandem Ubiquitin Binding Entities (TUBEs) with a significant decrease in the ubiquitylated fraction of HAPSTR1 being apparent in TUBE pulldowns from HUWE1-depleted cells (Fig. 5d). Whilst this might indicate HAPSTR1 interacts with another protein that is modified by HUWE1, the identification of HAPSTR1 as a substrate for HUWE1 in vivo^27^ and in vitro^28^ suggests HUWE1-dependent ubiquitylation of HAPSTR1 similarly occurs under our experimental conditions. Consistent with this, HUWE1 depletion, or treatment with the proteasome inhibitor MG-132, led to reduced turnover and increased Flag-HA-HAPSTR1 levels in cells (Fig. 5e and Supplementary Fig. 8e). Similarly, U2OS cells displayed increased abundance of endogenous HAPSTR1 upon HUWE1 depletion (Fig. 5f), or MG-132 treatment (Fig. 5g). Together, these data indicate that HUWE1 targets HAPSTR1 for ubiquitin-dependent proteolysis in our experimental system.

Given these findings, we next investigated whether HUWE1 disruption modulates PARP inhibitor sensitivity in BRCA2-deficient cells through HAPSTR1-dependent regulation of G4 structures. Similar to the effects observed with overexpression of recombinant FLAG-HA-HAPSTR1, siRNA-mediated knockdown of HUWE1 suppressed the elevated G4 levels induced by BRCA2 depletion (Fig. 5h). Importantly, this suppression was reversed in *HAPSTR1* KO cells, indicating that HAPSTR1 expression is required for HUWE1-mediated reversal of G4 accumulation. Furthermore, the reduction in G4 structures following HUWE1 depletion correlated with a rescue of PARP inhibitor sensitivity in BRCA2-deficient cells (Fig. 5i). Taken together, these data underscore HUWE1 dysfunction as a critical determinant of PARP inhibitor resistance in BRCA2-deficient cells through upregulation of HAPSTR1 and suppression of G4 structures.

## Discussion

Our analyses reveal that G4 structures contribute towards genome instability and PARP inhibitor sensitivity in cells. Previously, we and others demonstrated that transcription replication conflicts and R-loop formation contribute to PARP inhibitor toxicity in HAPSTR1 or BRCA2-deficient cells^8,9^. Here, we report that stabilising G4s contributes to this phenotype by inducing replication-associated DNA damage and genome instability. R-loop induction stabilises G4 structures^22–25,31^. Conversely, G4 stabilisation by PDS can enhance the formation of R-loops in the vicinity of G4s^16^. Our findings suggest that G4 accumulation and R-loop formation become uncoupled in HAPSTR1-deficient cells, particularly during DNA replication in the absence of G4 ligands. Although *HAPSTR1* KO cells show increased G4s, R-loops are not elevated under basal conditions (Fig. 1a)^8^. Moreover, G4 formation and replication-associated DNA damage are not suppressed by DRB (Fig. 1d and Supplementary Fig. 2a, b, d). Although these observations and S9.6 specificity were not further validated by suppressing R-loops by RNaseH expression, they nevertheless indicate DNA damage without R-loop formation. These data might also indicate a requirement for HAPSTR1 in DSB repair, rather than DNA damage suppression through G4 regulation. However, our observations that *HAPSTR1* KO cells are not sensitive to DSB-inducing agents would argue against this^8^. Intriguingly, upon PDS treatment, a proportion of 53BP1 foci formation in *HAPSTR1* KO cells become transcription-dependent (Supplementary Fig. 2d), implicating R-loops in generating DSBs in response to G4 ligands. However, suppression of 53BP1 foci is incomplete, supporting a continued contribution of G4s to DNA damage under these conditions. Notably, γH2AX remains DRB-resistant under these conditions, suggesting γH2AX reflects a primary response to G4 accumulation and replication stress, whereas 53BP1 marks downstream damage, potentially through replication fork breakage triggered by transcription–replication conflicts. Consistent with previous reports^11,15–19^, these data support a model in which G4s act as replication barriers that induce DNA damage signalling to γH2AX. However, they also support a transcription-dependent structure, such as R-loops contributing to secondary damage, particularly in response to PDS.

HAPSTR1 was initially characterised as a co-dependency hub within global stress response networks^27^. Intriguingly, although HAPSTR1 is a known substrate for HUWE1-mediated ubiquitination and turnover^27,28,30^, the positive correlation between HAPSTR1 and HUWE1 observed in co-essentiality analyses suggests a functional partnership rather than a purely degradative relationship^27^. Critically, however, this association appears to diverge under conditions of genotoxic stress. Loss of HUWE1 reduces γH2AX formation, whilst HAPSTR1 depletion results in a marked increase, indicating that the two proteins exert opposing influences on DNA damage signalling (Fig. 1d)^27^. This differential behaviour is replicated when assessing nuclear G4 levels (Fig. 5h), arguing HUWE1 and HAPSTR1 operate in a DNA damage response (DDR) and G4 regulation role that is distinct from the more pleiotropic stress response role of HAPSTR1. The sensitivity of HAPSTR1-deficient cells to ATR inhibition^29^ further implicates this pathway in replication stress at G4 structures, which are well-established barriers to fork progression. Mechanistically, our data indicate that HAPSTR1 interacts either directly or indirectly with G4 DNA in a manner that is competitive with PARP binding, although the precise mechanisms of how HAPSTR1 restrains G4 formation remain unclear. Nevertheless, these observations support a model in which HAPSTR1 functions at the interface of replication and repair, coordinating the removal or destabilisation of G4 and R-loop structures to prevent replication fork collapse and genome instability.

A key finding of our work is that PARP1 interacts with G4s in cells and functions in a pathway parallel to HAPSTR1 to regulate these structures. Several lines of evidence support this finding. Firstly, whilst PARP1/PARP2 disruption or PARP inhibition increases G4 levels, we observe a further increase in these structures when this is done in *HAPSTR1* KO cells (Fig. 2d). Furthermore, the elevated levels of PARP1 association with G4s (Fig. 2e) and increased ADPr in *HAPSTR1* KO cells (Fig. 4c) is consistent with an increased reliance on a PARP1-regulated mechanism when G4s are deregulated, explaining the toxicity of PARP1/PARP2 disruption in these cells. Importantly, we observe increased HAPSTR1 association with G4s in *PARP1/2* KO cells, or using inhibitors that do not trap PARP1/PARP2 (Fig. 2f, g). We therefore conclude that PARP dysfunction, as opposed to PARP trapping, is a key determinant of regulating G4 levels. Intriguingly, we observe that olaparib traps PARP1/2 at G4s, thereby suppressing the elevated accumulation of HAPSTR1 at these structures that we observe in *PARP1/2* KO cells, or cells exposed to veliparib (Fig. 2f, g). Despite this dominant-negative effect of olaparib on HAPSTR1–G4 interactions, we still detect an increase in nuclear G4 levels and heightened PDS sensitivity under these conditions (Fig. 2a, d). These findings suggest the existence of two G4 populations: one that is suppressed in a redundant manner by HAPSTR1 and PARP1/2, whilst another that is suppressed by PARP1 alone. Whatever the nature of these two G4 populations, these data are consistent with HAPSTR1 and PARP1/2 regulating G4 levels through parallel pathways.

G4 structures are potent barriers to replication forks in vitro^56–60^ and in cells^48,61^. In addition, exposure of cells to G4 ligands can result in DNA breakage at replication forks^17,62^. Given that G4 structures form at telomeres^38–40^ and that PARP1/2 have been implicated in telomere maintenance and ALT^35–37^, it is tempting to speculate that the accumulation of G4s in *HAPSTR1* KO and PARP inhibitor treated cells represents a telomere-specific phenotype. However, we observe increased G4 formation that is further enhanced upon exposure to PARP inhibitors in ALT-negative cell lines, and this is not restricted to telomeres (Supplementary Fig. 5a, b). We therefore believe our data represent a more general elevation of G4s levels, rather than it being restricted to telomeres. In this regard, PARP1 is known to bind G4s at telomeric and non-telomeric regions^41–46^. Disruption of PARP1 function may therefore deregulate G4 homoeostasis genome-wide, leading to elevated G4 levels that subsequently give rise to the G4–replication conflicts observed in this study. In addition, replication–G4 conflicts are increased in *HAPSTR1* KO cells (Fig. 2c and Supplementary Fig. 3a, b), even in the absence of exogenous genotoxic stress, resulting in replication-associated DNA damage (Fig. 1d and Supplementary Fig. 2c, d) and PARP activation (Fig. 4c). Given the role of PARP1/2 in replication fork recovery, these findings suggest that the elevated levels of G4s in HAPSTR1-deficient cells impede replication fork progression, thereby creating a dependency on PARP1 to resolve G4 structures and/or repair stalled or damaged forks.

Whilst PARP inhibitor toxicity in BRCA-deficient cells is associated with elevated ssDNA gaps^7^, the nature of the endogenous lesion that induces these structures is unclear. Here, we provide evidence that ssDNA gaps are induced at G4s in response to PARP inhibitors. HAPSTR1 functions in a parallel pathway to PARP1/PARP2 to suppress ssDNA gap formation and this mirrors the induction or suppression of G4 structures (Figs. 2d, 3a, b). Importantly, our data directly demonstrates replication fork stalling (Fig. 2c and Supplementary Fig. 3b) and ssDNA gap formation (Fig. 3g) at G4 structures upon PARP inhibition, providing a direct link between G4-mediated replication blocks and ssDNA gap accumulation. Furthermore, our findings that G4 induction occurs independently of PARP trapping indicate that ssDNA gap formation is not a consequence of replication conflicts with trapped PARP1/PARP2, but directly with G4s. We observe that ssDNA gaps in *HAPSTR1* KO cells exposed to PARP inhibitors are suppressed by PRIMPOL depletion and require TLS for repair (Fig. 3c–f). Of note, PRIMPOL can reprime ahead of G4 structures^63^ and REV1 disruption results in mutations adjacent to G4 motifs^64^. Given PRIMPOL repriming is primarily associated with leading strand synthesis^65^, our data suggest the structures we observe reside predominantly on the leading strand. Whether the same or distinct processes are employed to process G4 conflicts on the lagging strand is unknown. Nevertheless, our data clearly indicate that G4 structures are induced upon PARP inhibitor treatment and that these result in replication stress and ssDNA gaps.

Importantly, our data clearly point to HAPSTR1 and G4 status, providing an indication of PARP inhibitor responsiveness in BRCA2-deficient cells. HAPSTR1 and BRCA2 function in parallel pathways to regulate G4 homoeostasis, as evidenced by the increased sensitivity to PDS and accumulation of G4 structures when these genes are disrupted in combination (Fig. 4a, b). These observations are reminiscent of the findings that G4 ligands are toxic to BRCA2-deficient cells through inducing replication-associated DNA damage^18,19^. Notably, loss of BRCA2 results in elevated G4s at both telomeric and non-telomeric regions (Supplementary Fig. 7c), suggesting a genome-wide increase in G4s as a consequence of a role of BRCA2 in suppressing G4 levels, and/or resolving G4-replication conflicts. However, several lines of evidence suggest distinct populations of G4s are deregulated in response to loss of BRCA2, HAPSTR1 or PARP1. As highlighted above, our data analysing HAPSTR1 recruitment to G4s following trapping of PARPs at G4s by olaparib (Fig. 2d, f, g) suggests that one population of G4s is redundantly suppressed by HAPSTR1 and PARP1/2, whereas a distinct population is suppressed by PARP1 alone. Moreover, whilst BRCA2 and HAPSTR1 are epistatic, suggesting they function in the same pathway to suppress ssDNA gap accumulation (Fig. 4e), cells deficient in both BRCA2 and HAPSTR1 display elevated levels of G4s, particularly at non-telomeric G4s (Fig. 4b and Supplementary Fig. 7b, c), and enhanced sensitivity to PARP inhibitors (Fig. 4d). Whether elevated G4 levels in BRCA2/HAPSTR1 deficient cells is causal or consequence of ssDNA accumulation is unclear. Nevertheless, our data suggest that distinct subsets of G4 structures are differentially regulated by BRCA2, HAPSTR1, and PARP1/2. In support of this, although nearly 500,000 potential G4-forming sequences are predicted in the human genome, only a subset form at any given time, and distinct G4 populations are evident across different cell types^14,66^. Moreover, while deregulated G4 accumulation is toxic, G4 structures also perform important physiological functions, including transcriptional regulation^15^. Notably, G4s have been reported to either activate or repress transcription depending on promoter context, further reinforcing the idea that G4 structures can exert context-dependent functions across the genome.

Our findings indicate that HAPSTR1, BRCA2 and PARP1/PARP2 regulatory mechanisms all act on G4 structures to maintain cell viability. Our observations that cellular ADPr and PARP inhibitor sensitivity are further increased when BRCA2 and HAPSTR1 are disrupted in combination (Fig. 4c, d) indicate an increased reliance on PARP1/PARP2 for maintaining genome integrity and cell survival when G4s are deregulated. The well-established role of BRCA2 in replication fork recovery, taken together with our observations that elevated G4s in *HAPSTR1* KO cells leads to replication fork stalling and DNA damage, suggests that elevated G4 structures cause replication fork stalling that is repaired by a PARP-dependent mechanism in the absence of BRCA2. However, consistent with reports of G4 regulation at telomeres^40,53^ our data also indicate BRCA2 disruption elevates nuclear G4 levels themselves. Recently, the identification that BRCA2 is required to generate RNA–G4 hybrids (G-loops), which are necessary to facilitate G4 dissolution^25^, provides a potential mechanism for this observation. Together with our data, these findings indicate that BRCA2 may play a dual role in G4 regulation, acting not only through replication fork protection but also by directly influencing G4 homoeostasis. Notably, at least a subset of ssDNA gaps induced by HAPSTR1 loss localise to G4 structures (Fig. 3g). Given that BRCA2 is epistatic with HAPSTR1 for ssDNA gap formation, this indicates that BRCA2 is also required to suppress ssDNA gaps at a subset of G4s. Despite this, combined loss of BRCA2 and HAPSTR1 resulted in additive sensitivity to PARP inhibition, indicating additional factors to ssDNA gaps determine PARP inhibitor toxicity, at least in this context. Our data point to G4-mediated replication fork instability, most likely arising from a defect in homologous recombination^67^, as a cause of PARP inhibitor toxicity in the absence of BRCA2 and HAPSTR1.

Together, our findings support a model in which deregulated G4 structures compromise replication fork integrity, creating a heightened dependence on PARP-mediated pathways to maintain replisome stability and cell viability. This model is further supported by our findings that expression levels of HAPSTR1 influence G4 levels and the responsiveness of cells to PARP inhibitors. Together with the proposed role of HAPSTR1 as a stress response protein, this may suggest that variation in HAPSTR1 expression levels between cell lines influences cellular responses under different conditions, although this is unlikely to explain the phenotypes observed in the different HAPSTR1-deficient cell lines used here. Our data nevertheless indicate that increasing HAPSTR1 expression, either through directly overexpressing HAPSTR1 (Fig. 5a, b) or disrupting its negative regulator HUWE1 (Fig. 5h, i), reverses G4 accumulation and PARP inhibitor sensitivity in BRCA2-deficient cells. Intriguingly, HUWE1 disruption can suppress the sensitivity of BRCA2-deficient cells to PARPi through elevating the expression of RAD51 to restore HR^68^. Our data point to an additional mechanism by which HUWE1 status impacts on PARPi resistance through regulating HAPSTR1 expression, G4 levels and PARP inhibitor resistance in BRCA2-deficient cells. Given that tumour responses to PARP inhibitors are variable, and that resistance can develop, our study may highlight G4s as targets to overcome PARP inhibitor resistance.

## Methods

### Cell culture

The *HAPSTR1*Δ.2 and *HAPSTR1*Δ.3 cell lines correspond to *c16orf72*Δ.2 and *c16orf72*Δ.3 cells, respectively and along with the G5Δ.*HAPSTR1* RPE1 KO cell line that have been described previously^8^. All cells were maintained in DMEM (Sigma Aldrich, Cat D6546-500ml) supplemented with 10% heat inactivated foetal bovine serum (FBS; Sigma-Aldrich, F9665), L-glutamine (Sigma-Aldrich, G7513) and penicillin-streptomycin (Sigma-Aldrich, P0781). To obtain single-cell suspensions, attached cells were treated with trypsin-EDTA solution (Sigma-Aldrich, T3924) for 5–10 min at 37 °C.

### siRNA mediated knockdown

All gene-targeting small interfering RNA (siRNA) used in this study were ON-TARGETplus human SMARTPool from Dharmacon. Cells were transfected with 50 nM siRNA twice at 24 h intervals using Dharmafect-1 (Dharmacon) and used the next day for performing experiments.

The following siRNA obtained from Dharmacon were used: non-targeting siRNA pool (Cat# D-001810-10-05), siRNAs targeting BRCA1 (Cat# L-003461), BRCA2 (Cat# L-003462), PRIMPOL (Cat# L-016804), and HUWE1 (Cat# L-007185).

### Clonogenic survival assay

1000 cells were seeded in a 6-well plate and incubated overnight at 37 °C and 5% carbon dioxide. The next day, if required, cells were treated with drugs for the stipulated duration of time and allowed to grow into colonies for 10 to 15 days. Cells were fixed with 100 % methanol for 10 min at −20 °C, and stained with crystal violet (BDH Laboratory, Cat# 351884 W) for 20 min at room temperature. After washing and drying the plates, colonies with more than 50 cells were counted, and relative cell survival was calculated as a percentage with respect to untreated cells.

### Immunofluorescence

8 × 10^4^ cells were seeded on coverslips, placed in 12-well plates, and incubated with media at 37 °C and 5% carbon dioxide overnight. After the drug treatments described in the individual experiments were conducted, cells were fixed using 100 % methanol for 10 minutes at −20 °C. Following 2 × 5 min washes with PBS, cover slips were incubated with blocking solution containing 5% BSA + 0.1% FBS in 0.5% triton PBS for 30 min followed by incubation overnight with primary antibody at 4 °C. The next day, cover slips were washed 3x for 10 min with PBS and incubated for an hour with secondary antibody at room temperature. After 3 × 10 min washes with PBS, coverslips were mounted on glass slides using VECTASHEILD mounting media with DAPI (Vector Laboratories, Cat# H-1200-10). All incubations were done in a humidified dark chamber. Images were taken using an IX71 Olympus confocal microscope using the 60x objective. All images were analysed using ImageJ v1.54k.

For immunofluorescence using the S9.6 antibody, given it is known to recognise ribosomal RNA in addition to DNA:RNA hybrids, its specificity was validated in our previous study^8^. Briefly, methanol-fixed and permeabilised cells were treated with RNase H (5 U; NEB, M0297S) for 120 min at 37 °C prior to S9.6 staining, which resulted in a loss of signal, confirming specificity for DNA:RNA hybrids under these conditions.

For immunofluorescence to detect Pan-ADPr, 3.0 × 10^5^ cells were seeded in a 6 well-plate containing coverslips and then transfected with siRNA as indicated in the figure. Cells were washed with PBS followed by pre-extraction with CSK buffer (10 mM PIPES pH 7.5, 100 mM NaCl, 3 mM MgCl_2_, 300 mM Sucrose) supplemented with 0.5 % Triton X-100, 40 µM PJ-34 and 1 µM ADP-HPD on ice for 5 min. Coverslips were washed once with cold PBS and fixed in 4% formaldehyde in PBS for 10 min at room temperature. Following 3 × 5 min washes with cold PBS, coverslips were incubated with blocking solution (10% FBS in PBS) for an hour at room temperature, which was followed by incubation with the primary antibody overnight at 4 °C. Following 3 × 10 min washes with PBS, coverslips were incubated with the secondary antibody for an hour at room temperature. Following 3 × 10 min washes with PBS, coverslips were incubated in 0.5 mg/ml DAPI in PBS for 1 h at room temperature and then washed 2 × 5 min in PBS. Coverslips were then left to air dry before mounting on coverslips using Mowiol. Imaging was conducted on the Olympus ScanR using a 40x objective, under non-saturating conditions using the ScanR Acquisition Software (Version 3.5.0). Images were analysed using the Olympus ScanR Image Analysis Software (Version 3.5.0).

### Proximity ligation assay

8 × 10^4^ cells were seeded per coverslip in the 24-well plates, and treated as indicated in the figure legends before fixing with 100% methanol for 15 min at −20 °C. Cells on coverslips were then subjected to MERCK Duolink Proximity Ligation Assays following the manufacturer’s instructions (reagents used: DUO92004, DUO92002 and DUO92007). For negative controls, the protocol was performed with one primary antibody and the omission of the second primary antibody. Fixed cells were incubated with the appropriate primary antibody combinations for 2 h at 37 °C and then with PLUS and MINUS PROXIMITY LIGATION ASSAYS probes for 70 min at 37 °C. Ligation and amplification steps were then performed for 30 and 100 min respectively, at 37 °C. All incubations were done in a humidified dark chamber. Coverslips were fixed onto glass slides using the VECTASHEILD mounting media with DAPI (Vector Laboratories, Cat# H-1200-10). Images were taken using an IX71 Olympus confocal microscope using a 60x objective. All the images were analysed using ImageJ v1.54k.

### Immunoprecipitation

Approximately 3 × 10^7^ cells were used for each experiment. When the cells reached 60–80% confluence, they were washed twice with ice-cold 1x PBS and subsequently resuspended in Triton X-100 Lysis Buffer (100 mM NaCl, 1% Triton X-100, 50 mM Tris-HCl pH 8.0 supplemented with 5 mM N-ethylmaleimide, 1 mM DTT, 1x protease inhibitor cocktail, 1 mM sodium fluoride, 10 mM β-glycerophosphate, 0.1 mM sodium orthovanadate, 125 U/ml benzonase, 5 mM magnesium chloride) by scraping. Cell suspensions were incubated at 4 °C for 30 min with constant agitation before cell debris was removed by spinning at top speed for 30 min. For immunoprecipitation of Flag-tagged proteins, anti-Flag M2 affinity gel (Sigma, A2220) was used, whereas immunoprecipitation of HUWE1 involved the pre-incubation of HUWE1 polyclonal antibody (Bethyl, Laboratories, A300-486A) with Protein G Dynabeads (Invitrogen, 10004D) at 4 °C for at least an hour. Cell lysate was added to the beads and mixed for at least 3 h at 4 °C. As a negative control, equal volume of lysis buffer was added to the beads instead of the cell lysate. The beads were then washed five times with lysis buffer (minus the benzonase and magnesium chloride) and eluted by boiling in 2x SDS Loading Buffer (100 mM Tris-HCl, pH 6.8, 4% SDS, 0.1% Bromophenol Blue, 20% glycerol, 0.2 M DTT) for 10 min.

### HAPSTR1 Ubiquitination

The level of ubiquitination of HAPSTR1 was determined using Tandem Ubiquitin Binding Entities (TUBEs) pulldown assay following the protocol recommended by the manufacturer, albeit with minor changes. Briefly, U2OS cells were plated onto 15 cm dishes and transfected with 25 nM of siHUWE1 or non-targeting siRNA (siNT) as described above. The transfected cells were split onto six 15 cm dishes one day after the second transfection and allowed to grow at 37 °C for 36–48 h. When the cells reached 60–80% confluence, they were first washed twice with ice-cold 1x PBS and then lysed in Cell Lysis Buffer (50 mM Tris-HCl, pH 7.5, 0.15 M NaCl, 1 mM EDTA, 1% NP-40, 10% glycerol) by scraping the cells in the buffer. The cell suspension was then incubated at 4 °C with constant agitation for at least 30 min and subsequently clarified by spinning the cell suspension at top speed for 30 min at 4 °C. The purified cell lysate was divided into two (each half is equivalent to ~ 2 × 10^7^ cells); 200 µg of GST-TUBE2 (LifeSensors, Inc.; UM102) was added to one half of the lysate, and equimolar GST protein only (Sigma-Aldrich, SRP5348) was added to the other half as a negative control. The lysate-TUBEs mixture was incubated at 4 °C for at least one hour. Meanwhile, glutathione magnetic agarose beads (Pierce, 78602) were washed three times in TBS-T (20 mM Tris-HCl, pH 8.0, 0.15 M NaCl, 0.1% Tween-20) and an equivalent of 100 µl beads slurry was added to each sample. The lysate-TUBEs and beads mixture was incubated at 4 °C for at least three hours before collected and washed at least five times with TBS-T. Bound proteins were eluted by boiling in 2x SDS Loading Buffer (100 mM Tris-HCl, pH 6.8, 4% SDS, 0.1% Bromophenol Blue, 20% glycerol, 0.2 M DTT) for 10 min and subjected to SDS-PAGE and Western blotting.

### Slot Blot

Genomic DNA was extracted from the corresponding cell pellets using the DNeasy Blood & Tissue Kit (Cat. No.: 69504, QIAgen) following the manufacturer’s protocol. 3 µg of DNA was loaded per condition on positively charged nylon transfer membrane (Cat. No.: RPN203B, GE Healthcare) using the slot blot microfiltration system. The membrane was then crosslinked twice with 0.12 J/cm^2^ of UV at 365 nm and blocked in 5% milk in PBS-T. This was followed by incubation with the primary antibodies diluted in 5% milk in PBS-T overnight at 4 °C. Following 3 × 10 min washes in PBS-T, the membrane was incubated in secondary antibody diluted in PBS-T. The membrane was then washed 3 × 5 min in PBS-T before Immobilon Western Chemiluminescent HRP substrate (Cat. No.: WBKLS0500, Merck Millipore) was added. Images of the blots were captured using the Li-Cor Odyssey Fc imaging system, and quantification of the signal was conducted using Fiji.

### Native ssDNA gap immunofluorescence staining

To pre-label the DNA, 1.5 × 10^5^ cells were seeded either directly, or after the indicated siRNA-mediated knockdown onto coverslips and incubated in 10 μM CldU for 48 h in six-well plates. Depending on the experiment, cells were left untreated, or exposed to 10 μM olaparib for 2 h. Also, when assessing the effect of PDS on ssDNA gap formation, 10 μM of PDS was added at the same time as CldU for 48 h. After treatment, cells were treated as described previously^48^. Briefly, cells were washed with PBS and pre-extracted using PBS-T (0.5% Triton X-100 in PBS) on ice. Cells were then fixed with 4 % Formaldehyde (FA) for 15 min at RT and permeabilised in PBS-T again. Permeabilised cells were incubated with primary antibody against CldU (Rat monoclonal anti-BrdU; Abcam Cat# ab6326; 1:500) at 4 °C overnight. Cells were washed with PBS-T 3 x for 5 min and incubated with the secondary antibodies (Alexa Fluor^TM^ 555 goat anti-rat IgG (H + l) at 1:500; Invitrogen Cat# A21434) for 1 h at RT. After 3×5 min washes with PBS, coverslips were mounted onto glass slides using VECTASHEILD mounting media with DAPI (Vector Laboratories, Cat# H-1200-10). Images were taken using an IX71 Olympus fluorescent microscope using a 60x objective. All the images were analysed using ImageJ v1.54k.

### S1 nuclease DNA fibre assay

Cells were pulse-labelled with 25 μM CldU for 30 min, washed twice with warm PBS, pulse-labelled with 250 μM IdU for 120 min, and then washed with PBS as previously described previously^50^. For experiments using PARP inhibitors, cells were treated with 10 μM of Olaparib at the same time as IdU for 2 h. After IdU incorporation, cells were washed with PBS and permeabilized with CSK100 buffer (100 mM NaCl, 10 mM MOPS pH 7.0, 3 mM MgCl_2_, 300 mM sucrose, and 0.5 % Triton-X-100 in water) for 5 min at RT, washed once with S1 buffer (30 mM NaAc pH 4.6, 10 mM ZnAc, 5 % glycerol, 50 mM NaCl in water) and treated or not with S1 nuclease (20 U/ml) in S1 buffer for 30 min at 37 °C. After S1 nuclease treatment, cells were collected in PBS with 0.1 % BSA, pelleted for 5 min at 4 °C and resuspended in 100–150 μl of PBS. For spreading DNA into fibres, 2.5 μl of cells was added to a glass slide, lysed and immunofluorescently stained. Images were taken using an IX71 Olympus fluorescent microscope using a 60 x objective. All the images were analysed using ImageJ v1.54k.

For DNA fibre assay with G4s, U2OS cells were incubated with 25 µM CldU for 20 minutes. After labelling had finished, cells were harvested, and DNA fibres spread onto microscope slides. Immunostaining of DNA for CldU was performed, followed by overnight incubation with anti-G4 antibody and the next day incubation with secondary antibody. CldU labelled replication fork terminating with and without G4 foci were quantified using ImageJ. The images were obtained using an IX71 Olympus confocal microscope using a 100x objective.

To perform the DNA replication fork degradation assay, U2OS cells were sequentially labelled with two different nucleotide analogues to mark actively replicating DNA: 25 µM CldU for 30 minu, followed by 250 µM IdU for 3 hours with the addition of 10 µM olaparib. The cells were then trypsinised, and the labelled DNA strands were spread on glass microscope slides. DNA fibres were stained as described above, and the images were analysed using ImageJ v1.54k. The degradation of the newly formed DNA strand was visualised as a shortening of the second analogue marker (IdU) relative to the first (CldU).

### qPCR

Cell pellets were washed with PBS, and RNA was extracted using the RNeasy Plus Mini Kit (Cat. No.: 74134, QIAgen) following the manufacturer’s protocol. The homogenisation of the lysates was done using QIAshredder (Cat. No.: 79654, QIAgen). Approximately 600 ng of RNA was used to construct the cDNA using the High-Capacity cDNA Reverse Transcription Kit (Cat. No.: 4368814, Applied Biosystems). The cDNA samples were diluted 10-fold in nuclease-free water and subjected to qPCR using the QuantiNova SYBR Green PCR Kit (Cat. No.: 208056, QIAgen) using 550 nM of the following primers (Life Technologies); ActB_Fwd: CATGTACGTTGCTATCCAGGC, ActB_Rev: CTCCTTAATGTCACGCACGAT, HAPSTR1_Fwd: GTTCCTCCACCACGAAACTCT and HAPSTR1_Rev: TGCACCACTAAGACCATGCAGA.

### Antibodies

#### Primary antibodies

Mouse monoclonal anti-BrdU (B44) at 1:25 for DNA fibres (BD Biosciences, Cat# 347580); Rat monoclonal anti-BrdU at 1:400 for DNA fibres and 1: 500 for ssDNA assay (Abcam Cat# ab6326, clone BU1 / 75 (ICR1)); Rabbit recombinant monoclonal anti-BrdU antibody at 1:100 for proximity ligation assays (Abcam Cat# ab308341, HL1111); Mouse monoclonal anti-DNA G-quadruplex (G4) at 1:100 (Merck Life Science Cat# MABE1126, clone 1H6); Mouse anti-Poly (ADP-ribose) polymerase 1 (PARP1) at 1:1000 antibody (Bio-Rad Cat# MCA1522G); ɣH2Ax at 1:1000 (Millipore, Cat#. 05-636, Clone JBW301); Anti-poly-ADP-ribose binding reagent fused to rabbit Fc tag at 1: 400 (Millipore, Cat# MABE1016); dsDNA Marker Antibody at 1:2000 (Santa Cruz Biotechnology, Cat# sc-58749); S9.6 at 1:100 (Millipore, Cat# MABE1095); HUWE1 antibody (BETHYL, Cat# A300-486A); Rabbit monoclonal (anti-c16orf72) HAPSTR1 antibody at 1:1000 from Nicholas Lakin Lab (Sharma et al., 2023); Rabbit anti-HA Tag Polyclonal Antibody at 1:1000 (Invitrogen Cat# 71-5500, SG77); β-Actin at 1:5000 (Santa Cruz Biotechnology, Cat# sc-47778); Vinculin at 1:1000 (Cell Signalling Technology Europe BV, Cat# 4650).

#### Secondary antibodies

Alexa FluorTM 488 goat anti-mouse IgG (H + l) at 1:500 (Invitrogen Cat# A11029); Alexa FluorTM 488 chicken anti-rat IgG (H + l) at 1:500 (Invitrogen Cat# A21470); Alexa FluorTM PLUS 488 donkey anti-rabbit IgG (H + l) at 1:500 (Invitrogen Cat# A32790), Alexa FluorTM 555 goat anti-rat IgG (H + l) at 1:500 (Invitrogen Cat# A21434); Goat anti-mouse Alexa FluorTM 555 pAb at 1:500 (Abcam, Cat# ab150114), Polyclonal Goat anti-Mouse immunoglobulins/HRP at 1:10,000 for western blot, 1:2000 for dsDNA and 1:500 for G4 (Agilent Dako, Cat# P0447); Polyclonal Goat anti-Rabbit immunoglobulins/HRP at 1:10,000 (Agilent Dako, Cat# P0448); Polyclonal Swine anti-Rabbit immunoglobulins/TRITC at 1:10,000 (Agilent Dako, Cat# R0156); Polyclonal Rabbit anti-Mouse immunoglobulins/FITC at 1:10,000 (Agilent Dako, Cat# F0313).

### Chemicals reagents

Olaparib (SelleckChem, Cat# AZD2281) and Veliparib (Stratech Scientific Ltd, ABT-888 Cat# A3002-APE), JH-RE-06 (REV1i) (Glixx Laboratories, Cat# GLXC-21219), Pyridostatin hydrochloride (PDS) (Sigma-Aldrich, SML2690). MG-132 (Stratech Scientific Ltd, Cat# A2585-APE), and all compounds were dissolved in dimethyl sulfoxide (DMSO, Sigma-Aldrich, Cat# D5879) or RNase-free double-distilled H_2_O as a solvent. DNA labelling, thymidine analogues such as IdU (5-Iodo-2′-deoxyuridine, Sigma-Aldrich, Cat# I7125), CldU (5-chloro-2′-deoxyuridine, Sigma-Aldrich, Cat# C6891), and EdU (5-ethynyl-2′-deoxyuridine, Thermo Fisher Scientific, Cat# A10044). Enzymatic digestion was performed using S1 nuclease (Thermo Fisher Scientific, Cat# 18001-016), while detergents like Triton X-100 (Sigma-Aldrich, Cat# T8787) and solvents such as acetone (Sigma-Aldrich, Cat# 34850) and methanol (Sigma-Aldrich, Cat# 34860) were used for cell lysis and fixation. Bovine Serum Albumin (BSA) Fraction V (Merck Life Science, Cat# 10735086001) was used for the blocking solution, FLUOROSHIELD (Merck Life Science, Cat# F6182) mounting media for DNA fibre assay. For DNA synthesis detection, Click-iT™ EdU Alexa Fluor™ 647 (Invitrogen, Cat# C10340) was used to visualise nascent DNA replication.

### Statistics

The relevant statistical tests are stated where applicable. In each case statistical significance was analysed: *p  <  0.05; **p  <  0.01; ***p  <  0.001; ****p  <  0.0001. Otherwise, analyses were classified as not significant (ns). Student’s t tests were two-tailed and unpaired. Precise n values and exact *p* values are included in the Source Data. All the statistical analysis was performed using GraphPad Prism 10.

### Reporting summary

Further information on research design is available in the Nature Portfolio Reporting Summary linked to this article.

## Supplementary information

Supplementary Information

Reporting Summary

Transparent Peer Review file

## Source data

Source Data

## Data Availability

All data generated or analysed during this study are included in this published article and the Supplementary Information. Source data are provided in this paper.
