# Supplementary material for: G-quadruplex homeostasis is a determinant of PARP inhibitor toxicity in BRCA2-deficient cells
Source: Nat Commun. 2026 Jul 3;17:8275. doi: 10.1038/s41467-026-75083-9 (PMC13470221; doi:10.1038/s41467-026-75083-9)
Supplement: Supplementary file 1 [file 41467_2026_75083_MOESM1_ESM.pdf]

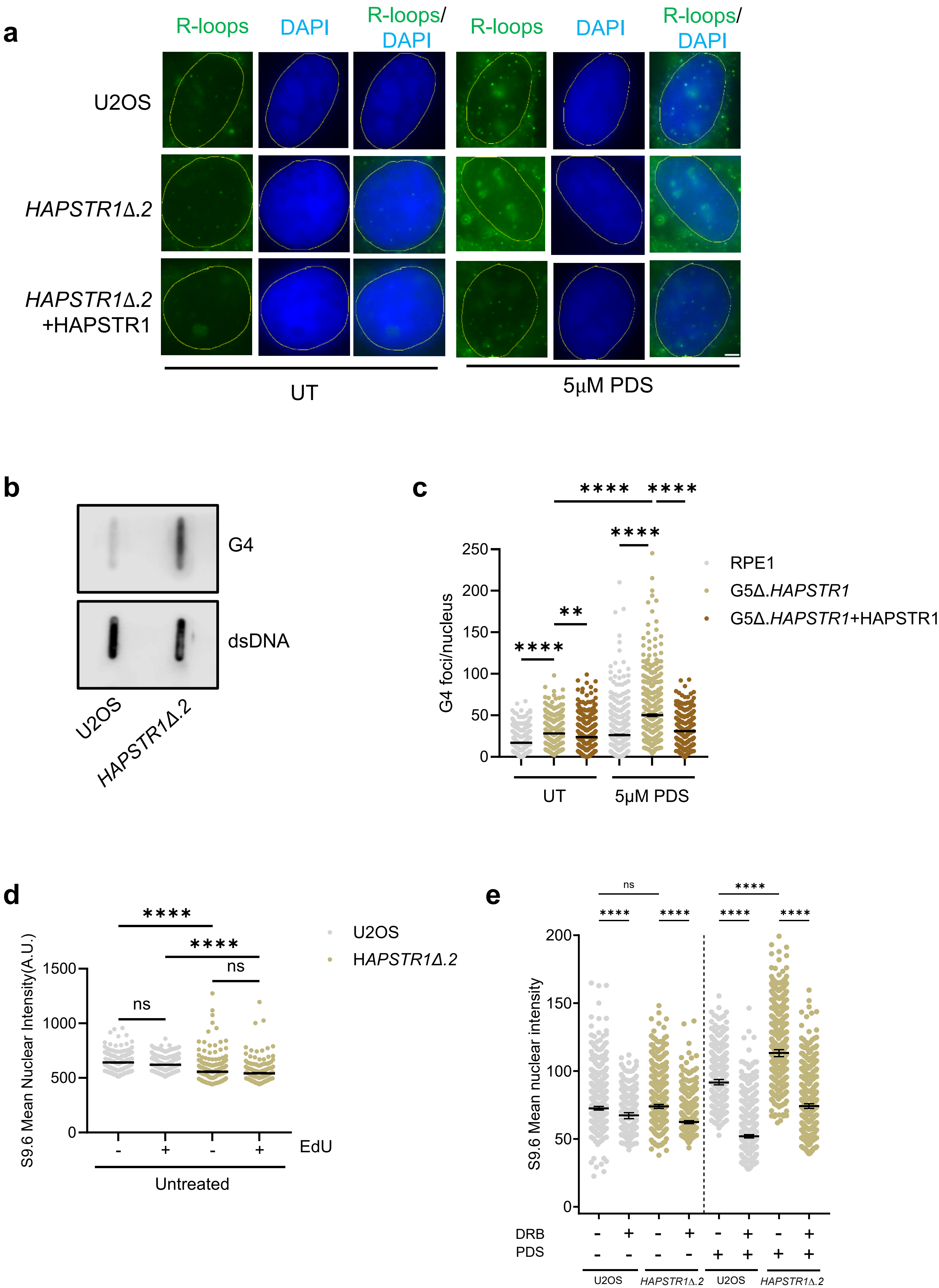
a
R-loops
DAPI
R-loops/ DAPI
U2OS
HAPSTR1Δ.2
HAPSTR1Δ.2 +HAPSTR1
UT
5μM PDS
b
G4
dsDNA
U2OS
HAPSTR1Δ.2
c
G4 foci/nucleus
RPE1
G5Δ.HAPSTR1
G5Δ.HAPSTR1+HAPSTR1
UT
5μM PDS
d
S9.6 Mean Nuclear Intensity(A.U.)
U2OS
HAPSTR1Δ.2
EdU
Untreated
e
S9.6 Mean nuclear intensity
DRB
PDS
U2OS
HAPSTR1Δ.2

Supplementary Figure 1: a Representative images from three independent biological replicates corresponding to the quantification in Figure 1a. Nuclear R-loops are shown in green, nuclei are counterstained with DAPI (blue), and yellow outlines mark the quantified nuclear regions. **b** Genomic DNA extracted from U2OS and *HAPSTR1* KO cells was subjected to slot blot and probed with anti-dsDNA and anti-G4 antibodies as indicated. Representative images from 2 independent experiments are illustrated. **c** Parental RPE1 or G5Δ.*HAPSTR1* with or without Flag-HA-HAPSTR1 expression were treated with 5µM PDS and nuclear G4 levels assessed by immuno-florescence. **d** U2OS and *HAPSTR1* KO cells were pulsed with EdU to label cells undergoing DNA replication. While R-loops were co-stained by immunofluorescence using the S9.6 antibody, followed by incubation with an fluorescent 488-conjugated secondary antibody, to avoid bleed through between channels, a 647 Click-iT EdU assay was performed to identify EdU-positive cells. Data represent the mean nuclear intensity of S9.6 staining ± SEM from three independent experiments, with at least 170 cells analyzed per treatment. The quantified values represent the overall nuclear mean intensity, which includes the grey value contributions from discrete R-loop foci (see Supplementary Figure S1a). **e** U2OS and *HAPSTR1* KO cells were left untreated or exposed to 100 µM DRB and 20 µM PDS for 4 and 3 hours, respectively. The cells were then stained by immunofluorescence using the S9.6 antibody, followed by incubation with a fluorescent secondary antibody conjugated to 488 dye and captured by immunofluorescence microscopy. Data show the median with 95% confidence interval (CI) of nuclear intensity of S9.6 staining from three independent experiments, with at least 420 cells analyzed per condition. Statistical significance was assessed by Kruskal Wallis non-parametric test. (*$p \leq 0.05$, **$p \leq 0.01$, ***$p \leq 0.001$, ****$p \leq 0.0001$ and ns = not significant).

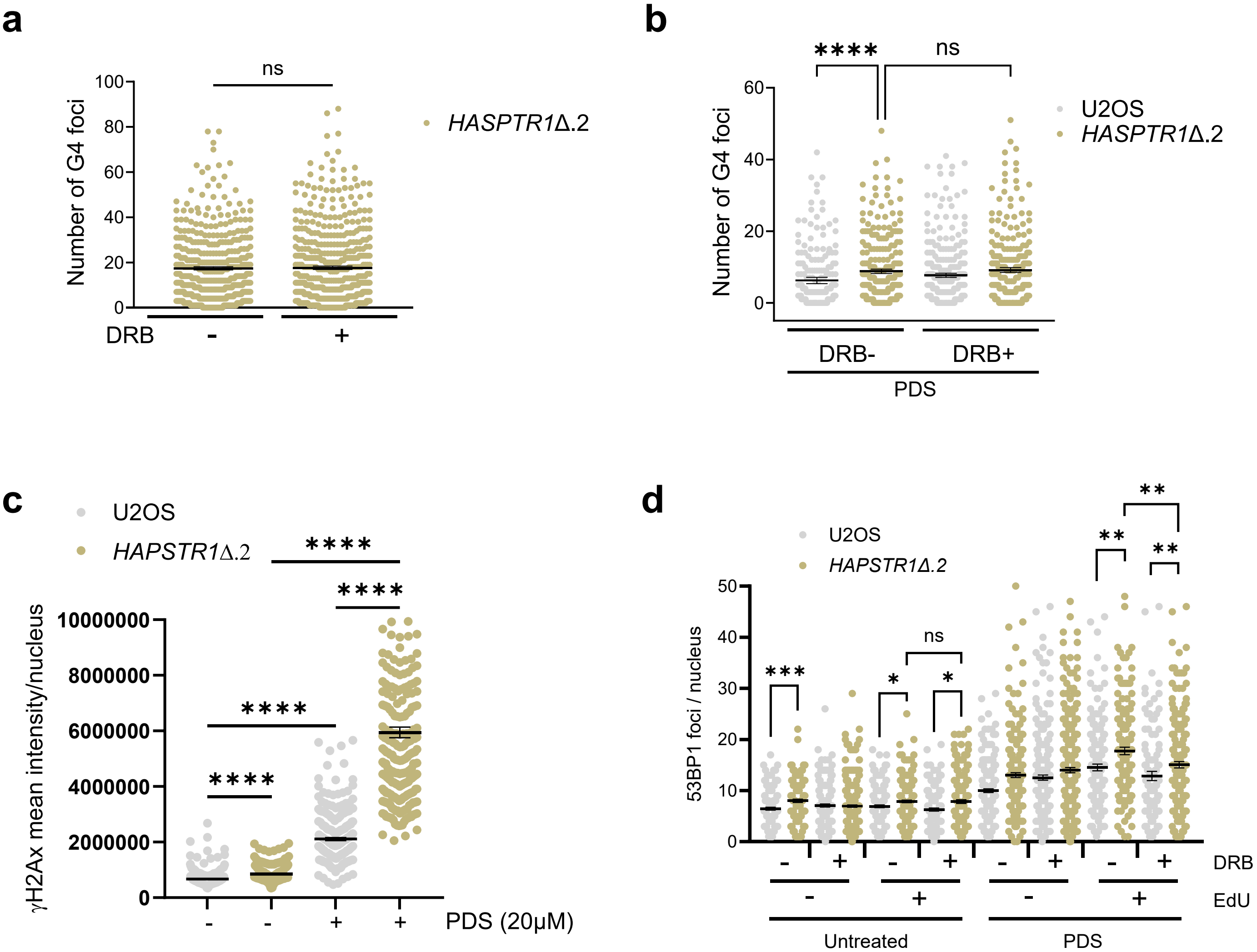

Supplementary Figure 2: a *HAPSTR1Δ.2* cells were treated with 100μM DRB for 4 hours and G4 levels assessed via immunofluorescence using the anti-G4 antibody. Mean values are from 3 independent experiments, with at least 413 cells examined per condition. **b** Parental U2OS and *HAPSTR1*Δ.2 were treated with 20μM PDS and/or 100μM DRB for 4 hours as indicated. G4 levels were assessed via immunofluorescence using an anti-G4 antibody. Mean values are from 2 independent experiments, with at least 219 cells examined per condition. **c** U2OS and *HAPSTR1* KO cells were left untreated or exposed to PDS for 72 hours as indicated and subjected to immunofluorescence using a γH2AX antibody. Data presented are the mean from at least 585 cells/condition of 3 independent experiments. Error bars are +/-SEM. Statistical significance was assessed by Kruskal Wallis non-parametric test. (*$p \leq 0.05$, **$p \leq 0.01$, ***$p \leq 0.001$, ****$p \leq 0.0001$ and ns = not significant). **d** U2OS and *HAPSTR1*Δ.2 cells were incubated -/+ 50μM PDS and -/+ 100μM DRB for 3 hours and 53BP1 foci were counted via immunofluorescence microscopy. Cells were also pulsed with EdU to mark cells undergoing replication at the time of treatment. Mean values +/- SEM from 3 independent experiments, with at least 238 cells examined per treatment condition are represented

a

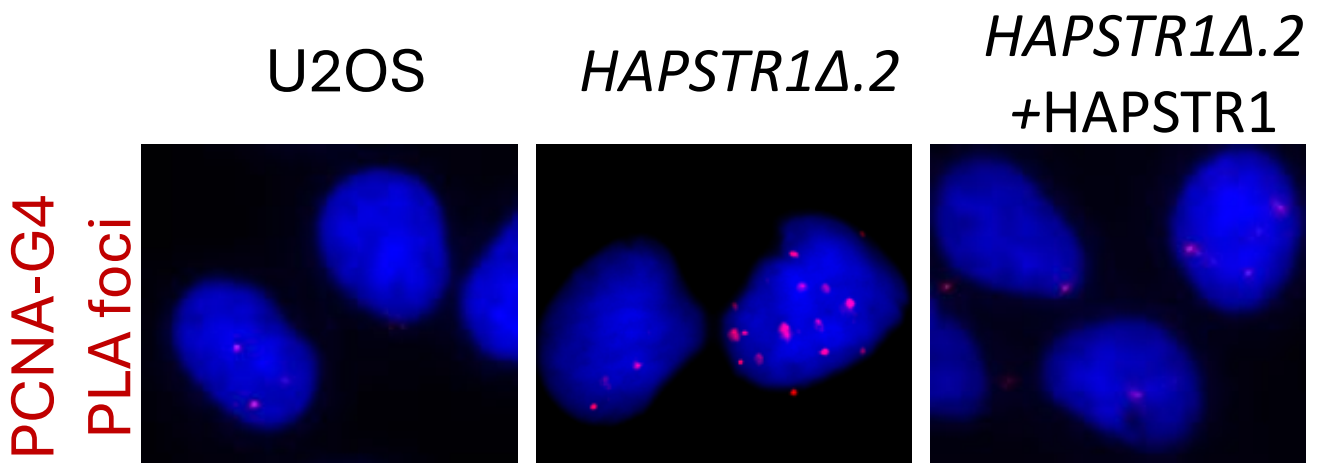

b

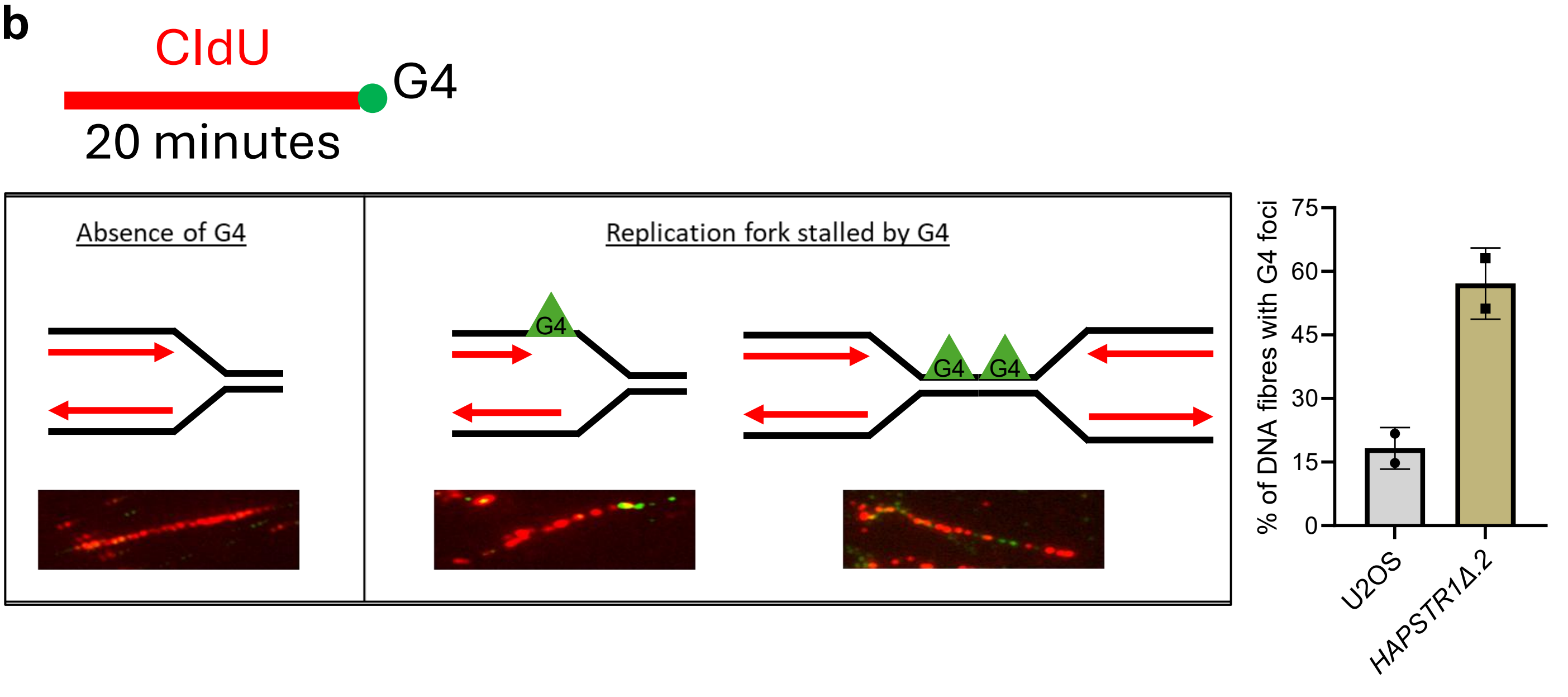

Supplementary Figure 3: Replication fork are stalled at G4 sites in absence of HAPSTR1. a Representative pictures for the proximity ligation assay (PLA) foci observed in U2OS, *HAPSTR1Δ.2* and *HAPSTR1Δ.2* cells complemented with HAPSTR1 expression using the anti G4 and PCNA antibody. **b** U2OS and *HAPSTR1* KO cells were subjected to DNA fibre assay following a 20 minutes pulse with CldU using anti-BrdU and anti-G4 antibodies. Representative pictures are representative of DNA fibres quantified as CldU stained DNA fibres (red) terminating with G4 staining (green). A total of at least 186 DNA fibres/condition were examined from 2 biological repeats. Data presented are the percentage of DNA fibres terminating with G4 staining.

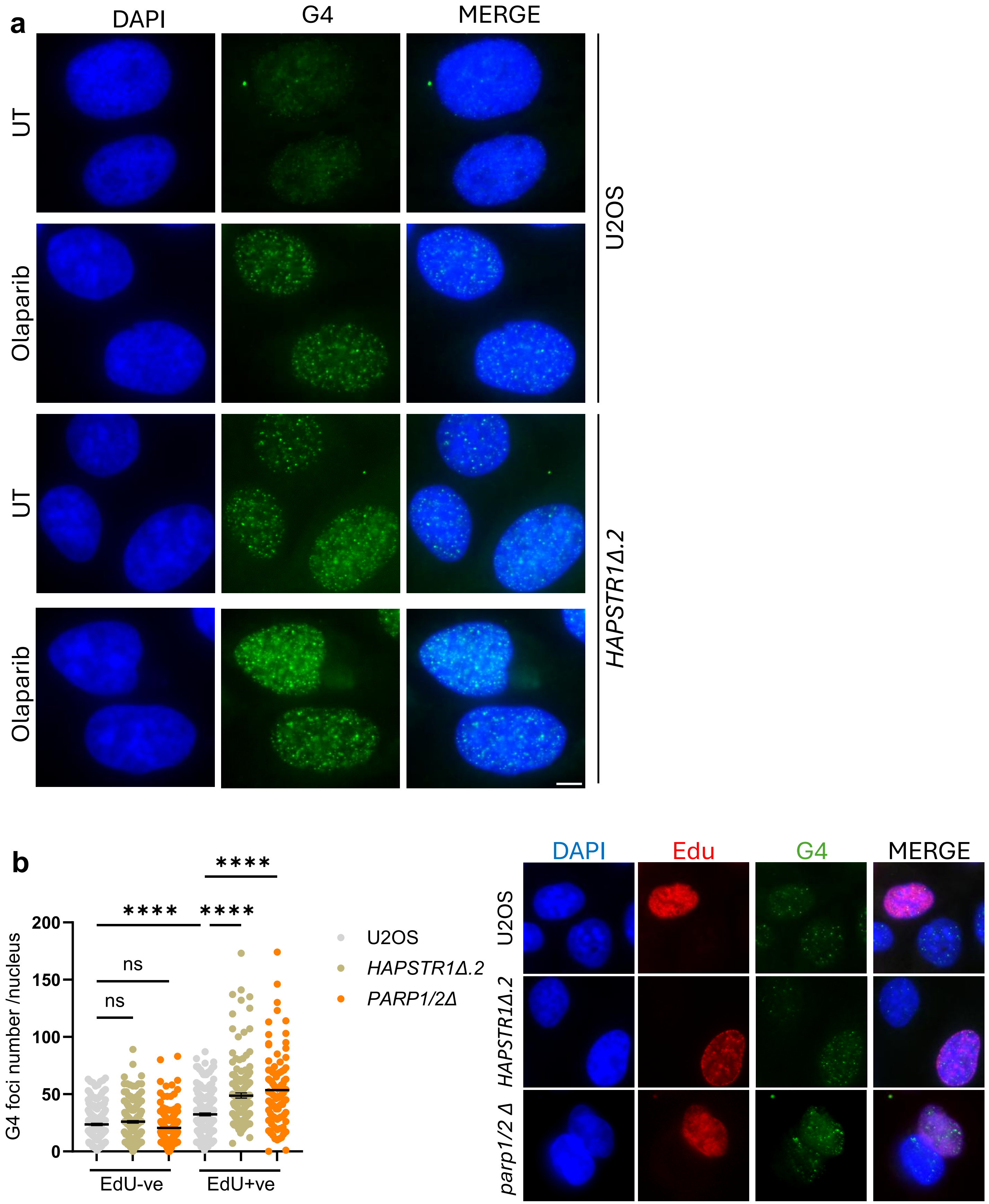

Supplementary Figure 4: Disruption of PARP1/PARP2 results in elevated G4 levels during DNA replication. a Representative immunofluorescence images used for G4 foci (green) quantification in nuclei (blue) presented in Figure 2d. Pictures are the representative of three biological repeats. **b** U2OS and *parp1/2Δ* cells were incubated with 10μM EdU to mark cells undergoing DNA replication prior to assessing nuclear G4 levels by immuno-florescence. Mean values +/- SEM from 3 independent experiments are represented, with at least 153 cells examined/condition. Representative images are provided (right panel)

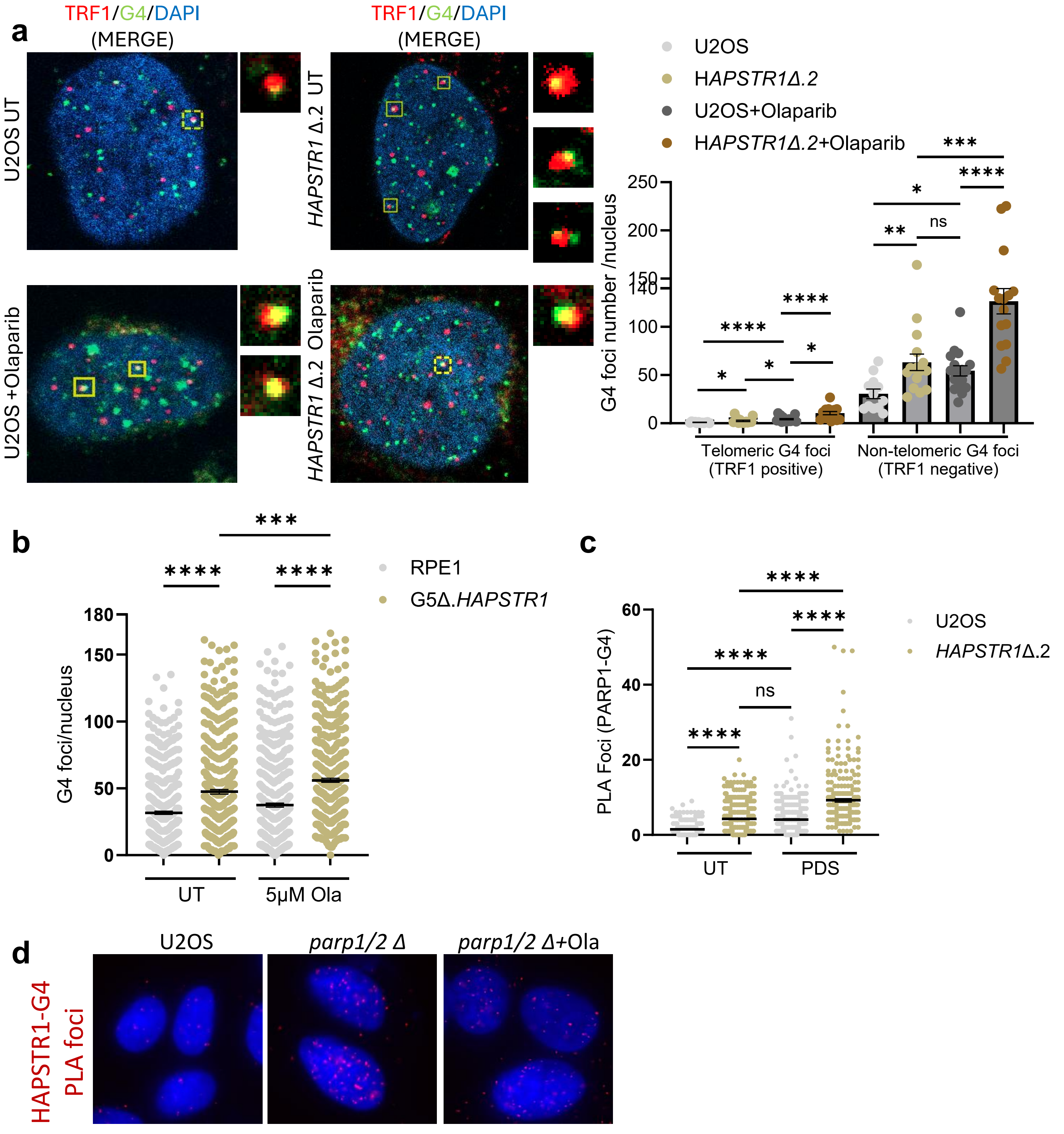

Supplementary Figure 5: Elevated nuclear G4 levels in HAPSTR1 KO cells exposed to PARP inhibitors are not restricted to telomeres and are independent of ALT status.
a U2OS and *HAPSTR1Δ.2* cells were treated with 5μM Olaparib for 3 hours prior to analysis of the nuclear G4 and TRF1 foci co-localisation by immunofluorescence. Co-localisation analysis was done using the ImageJ JACoP plugin. The images are the representative of the three biological repeats. **b** RPE1 control and *HAPSTR1Δ.2 (G5Δ.HAPSTR1)* cells were treated with 5μM Olaparib for 5 hours and subjected to immunofluorescence using the anti-G4 antibody and stained for nucleus with DAPI. The data presented is the quantification of three biological repeats from at least 426 cells examined/condition. **c** U2OS and *HAPSTR1*Δ.2 cells were treated with 5μM PDS for 24 hours and subjected to proximity ligation assays using the anti-PARP1 and anti-G4 antibodies, co-stained with DAPI to visualise and analyse nuclear PLA foci using the ImageJ. The data presented is the quantification of three biological repeats with at least 270 cells examined/ condition. **d** Representative images of HAPSTR1-G4 PLA foci (red) in nucleus stained with DAPI (blue) for the data presented in Figure 2g**.**

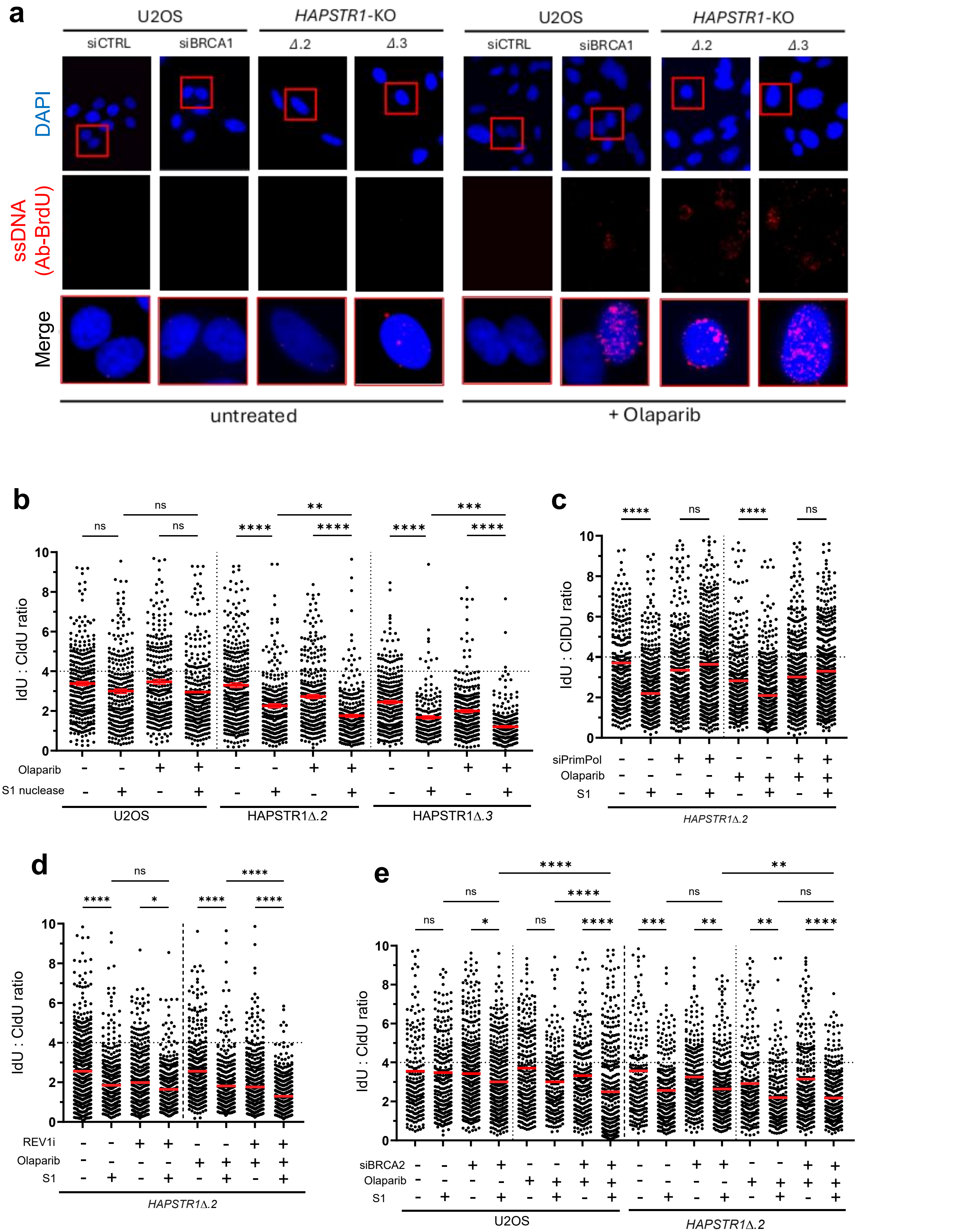

Supplementary Figure 6: HAPSTR1 loss leads to ssDNA gaps. Representative images of data analysed in Figure 3a. **a** Cells were co-stained for DNA (DAPI; blue) and ssDNA gaps (anti-BrdU antibody; red). The red boxes (top panel) are enlarged and displayed in the bottom two panels for ssDNA alone or merged with DAPI staining as indicated. **b-e** Data presented in Figure 3b (b), Figure 3c (c), Figure 3e (d) and Figuren 4e (e) expressed as IdU/CldU fibre length ratios. Data represent mean values ± SEM of 3 biological independent experiments, with the same number of DNA fibres scored as stated in the main figures.

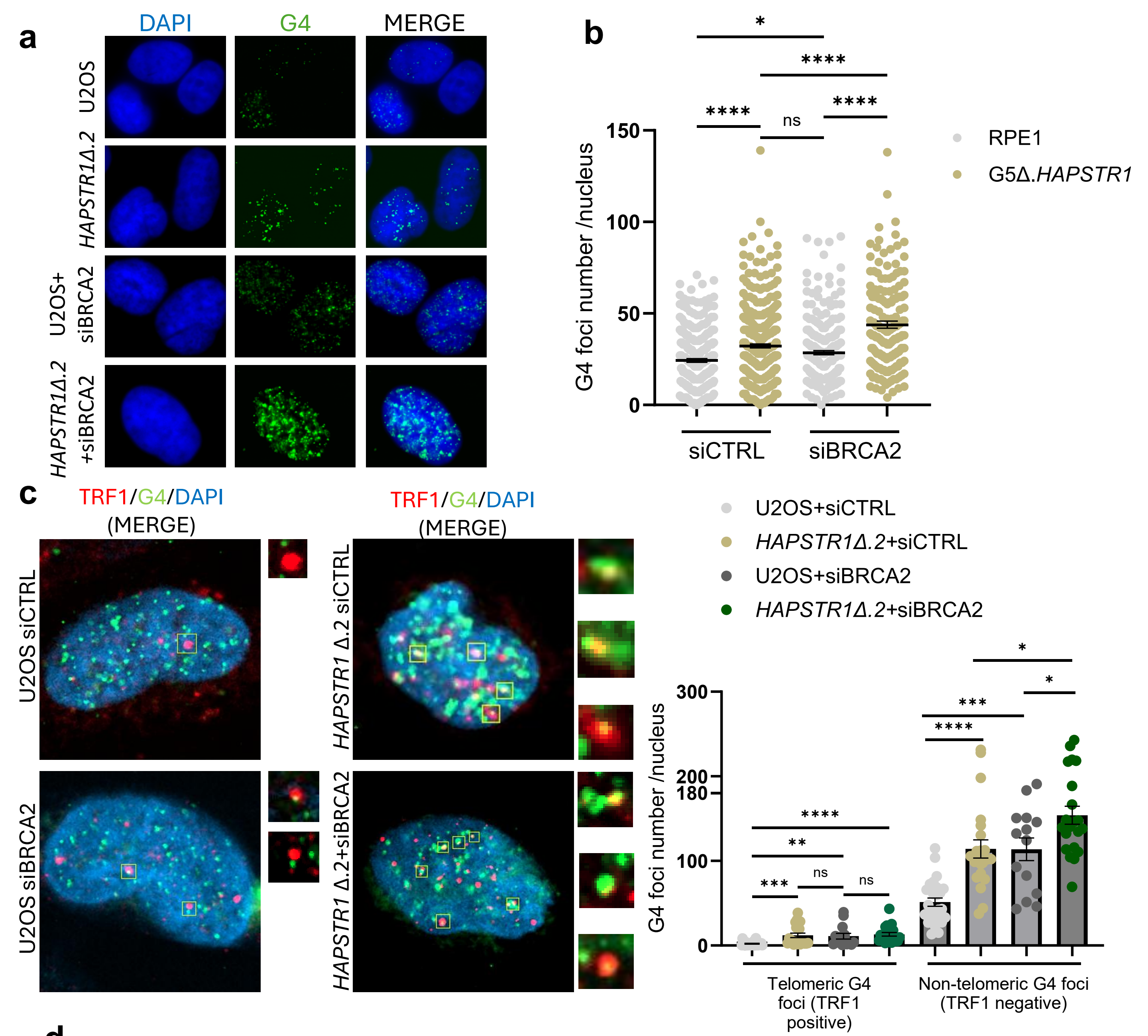

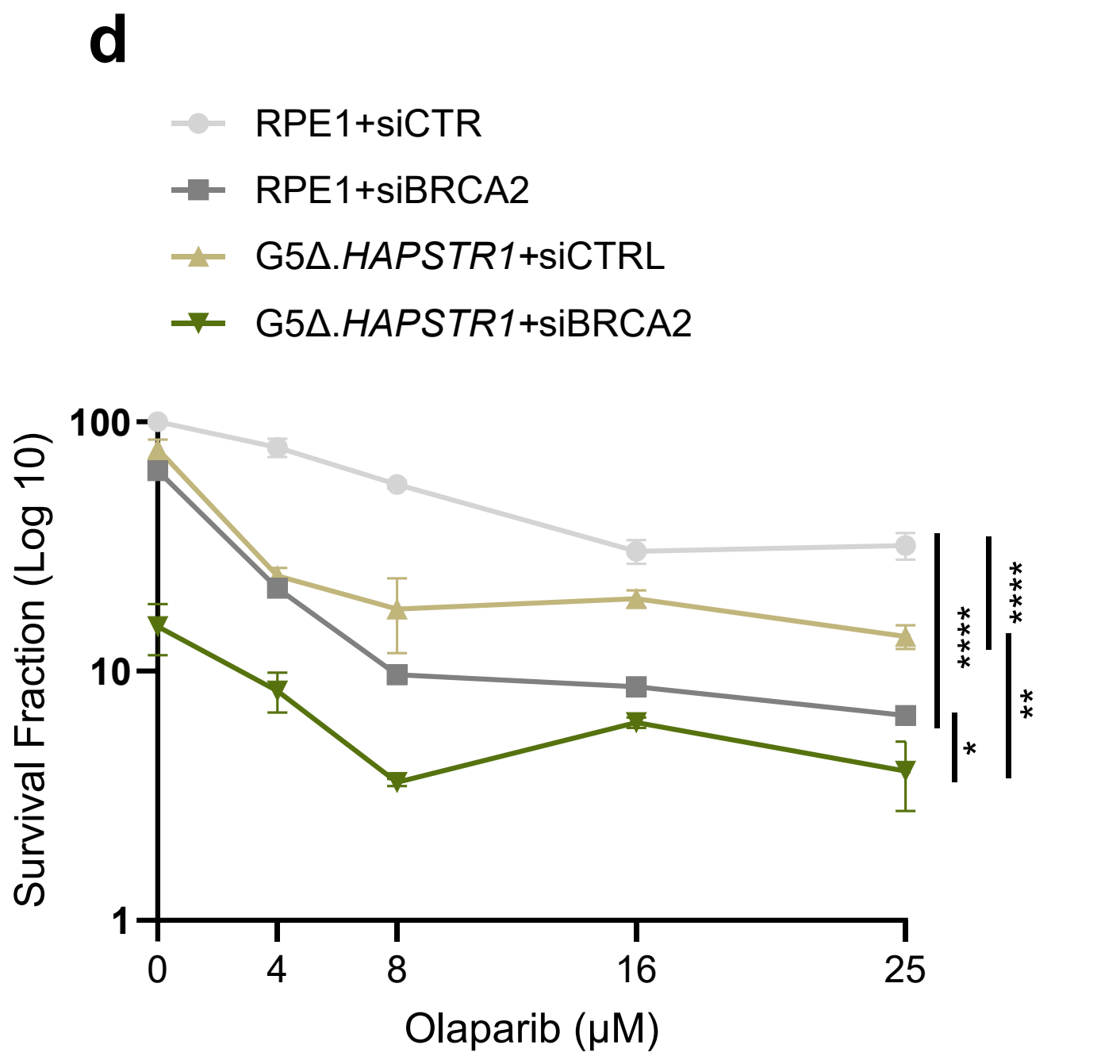

Supplementary Figure 7: Elevated nuclear G4 levels in HAPSTR1 KO and BRCA2 depleted cells are not restricted to telomeres and are independent of ALT status.

a Representative pictures of three biological repeats of the data presented in Figure 4a. **b** RPE1 control and *HAPSTR1Δ.2 (G5Δ.HAPSTR1)* cells were transfected with the indicated siRNA and analysed for G4 levels for at least 174 cells/condition from three biological repeats. **c** U2OS and *HAPSTR1Δ.2* transfected with indicated siRNA prior to analysis of the nuclear G4 and TRF1 foci co-localisation by immunofluorescence. Co-localisation analysis was done using the ImageJ JACoP plugin. Data represent mean values ± SEM of 3 independent experiments. The images are the representative of the three biological repeats. **d** Clonogenic survival assay of parental RPE1 and *HAPSTR1Δ.2 (G5Δ.HAPSTR1)* cells with indicated increasing concentration of Olaparib. Data represent mean values ± SEM of 4 independent experiments

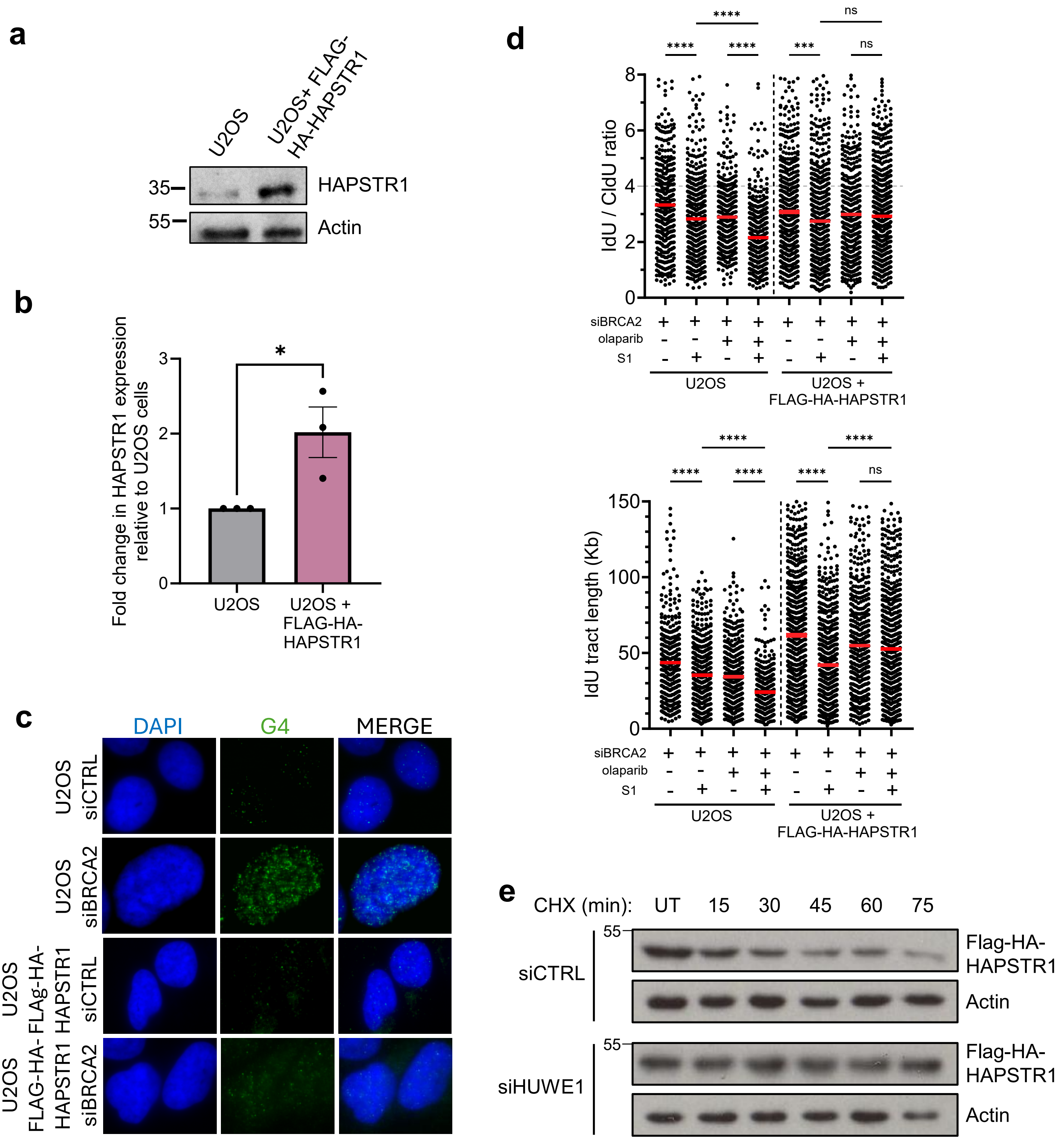

Supplementary Figure 8: HAPSTR1, HUWE1 and BRCA2 expression corelate with G4 levels.
a Whole cell extracts from U2OS cells with and without expression of Flag-HA-HAPSTR1 were subjected to western blotting with the indicated antibodies. **b** qPCR of HAPSTR1 in U2OS cells with and without expression of Flag-HA-HAPSTR1. The primers used in qPCR recognise both endogenous HAPSTR1 and FLAG-HA-HAPSTR1. As such, the bar plot represents the fold change in overall HAPSTR1 expression relative to U2OS cells from 3 biological repeats. ActB was used as a loading control. Error bars represent mean ± SEM. **c** U2OS and U2OS+FLAG-HA-HAPSTR1 cells transfected with the indicated siRNAs were analysed for G4 foci (green). Images are representative of three independent biological replicates; quantification is shown in Figure 5a. **d** U2OS cells expressing FLAG-HA-HAPSTR1 were pulsed with CldU (30 min) then next with IdU (120 min) (with or without 10 μM olaparib) followed by S1 nuclease digestion. Data represent IdU length (kb) (bottom panel) and IdU/CldU fibre length ratios (top panel). Mean values ± SEM of 3 biological independent experiments, with at least 399 DNA fibres scored/condition. **e** Western blot analysis using the indicated antibodies to assess the level of HA-tagged HAPSTR1 in cells transfected with siHUWE1 or non-targeting siRNA (siCTRL) and treated with 50 µg/ml of cycloheximide (CHX) for 0 to 75 min. Depletion of Flag-HA-HAPSTR1 following CHX treatment indicates turnover of proteins.
