# Supplementary material for: G-quadruplex homeostasis is a determinant of PARP inhibitor toxicity in BRCA2-deficient cells
Source: Nat Commun. 2026 Jul 3;17:8275. doi: 10.1038/s41467-026-75083-9 (PMC13470221; doi:10.1038/s41467-026-75083-9)
Supplement: Supplementary file 2 [file 41467_2026_75083_MOESM2_ESM.pdf]

nature portfolio

Corresponding author(s): Nicholas D. Lakin

Last updated by author(s): Jun 5, 2026

Reporting Summary

Nature Portfolio wishes to improve the reproducibility of the work that we publish. This form provides structure for consistency and transparency in reporting. For further information on Nature Portfolio policies, see our Editorial Policies and the Editorial Policy Checklist.

Statistics

For all statistical analyses, confirm that the following items are present in the figure legend, table legend, main text, or Methods section.

n/a	Confirmed	
☐	☒	The exact sample size (*n*) for each experimental group/condition, given as a discrete number and unit of measurement
☐	☒	A statement on whether measurements were taken from distinct samples or whether the same sample was measured repeatedly
☐	☒	The statistical test(s) used AND whether they are one- or two-sided<br>*Only common tests should be described solely by name; describe more complex techniques in the Methods section.*
☒	☐	A description of all covariates tested
☒	☐	A description of any assumptions or corrections, such as tests of normality and adjustment for multiple comparisons
☐	☒	A full description of the statistical parameters including central tendency (e.g. means) or other basic estimates (e.g. regression coefficient) AND variation (e.g. standard deviation) or associated estimates of uncertainty (e.g. confidence intervals)
☐	☒	For null hypothesis testing, the test statistic (e.g. *F*, *t*, *r*) with confidence intervals, effect sizes, degrees of freedom and *P* value noted<br>*Give P values as exact values whenever suitable.*
☒	☐	For Bayesian analysis, information on the choice of priors and Markov chain Monte Carlo settings
☒	☐	For hierarchical and complex designs, identification of the appropriate level for tests and full reporting of outcomes
☒	☐	Estimates of effect sizes (e.g. Cohen's *d*, Pearson's *r*), indicating how they were calculated

Our web collection on statistics for biologists contains articles on many of the points above.

Software and code

Policy information about availability of computer code

Data collection	IX71 Olympus confocal microscope using 60x and 100x objectives, Alpha Innotech Gel Imaging system, Liqor
Data analysis	ImageJ 1.54k, Microsoft Excel 2016, Prims Graphpad 10

For manuscripts utilizing custom algorithms or software that are central to the research but not yet described in published literature, software must be made available to editors and reviewers. We strongly encourage code deposition in a community repository (e.g. GitHub). See the Nature Portfolio guidelines for submitting code & software for further information.

Data

Policy information about availability of data

All manuscripts must include a data availability statement. This statement should provide the following information, where applicable:

- Accession codes, unique identifiers, or web links for publicly available datasets
- A description of any restrictions on data availability
- For clinical datasets or third party data, please ensure that the statement adheres to our policy

All data generated or analysed during this study are included in this published article and the Supplementary Information. Source data are provided with this paper.

Research involving human participants, their data, or biological material

Policy information about studies with human participants or human data. See also policy information about sex, gender (identity/presentation), and sexual orientation and race, ethnicity and racism.

Reporting on sex and gender	Not Applicable
Reporting on race, ethnicity, or other socially relevant groupings	Not Applicable
Population characteristics	Not Applicable
Recruitment	Not Applicable
Ethics oversight	Not Applicable

Note that full information on the approval of the study protocol must also be provided in the manuscript.

Field-specific reporting

Please select the one below that is the best fit for your research. If you are not sure, read the appropriate sections before making your selection.

☒ Life sciences ☐ Behavioural & social sciences ☐ Ecological, evolutionary & environmental sciences

For a reference copy of the document with all sections, see nature.com/documents/nr-reporting-summary-flat.pdf

Life sciences study design

All studies must disclose on these points even when the disclosure is negative.

Sample size	As is standard practice for the field, all the experiments were concluded by independent biological repeats as specified in the figure legends
Data exclusions	No data was excluded from the analysis
Replication	Data is derived from 3-5 independent biological experiments as specified in Figure legends. All repeats were successful and where possible data used to perform statistical analysis.
Randomization	Samples were allocated into the group depending on cell treatments as describe in the manuscript. Due to the relatively small group numbers randomization was not employed during these experiments
Blinding	Where possible (e.g. immunoflourescence), samples were scored blind. In certain experiments (e.g. western blotting) it was not possible to anonymise the data.

Reporting for specific materials, systems and methods

We require information from authors about some types of materials, experimental systems and methods used in many studies. Here, indicate whether each material, system or method listed is relevant to your study. If you are not sure if a list item applies to your research, read the appropriate section before selecting a response.

Materials & experimental systems

n/a	Involved in the study	
☐	☒	Antibodies
☐	☒	Eukaryotic cell lines
☒	☐	Palaeontology and archaeology
☒	☐	Animals and other organisms
☒	☐	Clinical data
☒	☐	Dual use research of concern
☒	☐	Plants

Methods

n/a	Involved in the study	
☒	☐	ChIP-seq
☒	☐	Flow cytometry
☒	☐	MRI-based neuroimaging

Antibodies

Antibodies used	Primary Antibodies<br>Mouse monoclonal anti-BrdU (B44) at 1:25 for DNA fibres (BD Biosciences, Cat# 347580);

Rat monoclonal anti-BrdU at 1:400 for DNA fibres and 1: 500 for ssDNA assay (Abcam Cat# ab6326, clone BU1 / 75 (ICR1));
Rabbit recombinant monoclonal anti-BrdU antibody at 1:100 for PLA (Abcam Cat# ab308341, HL1111);
Mouse monoclonal anti-DNA G-quadruplex (G4) at 1:100 (Merck Life Science Cat# MABE1126, clone 1H6);
Mouse anti-Poly (ADP-ribose) polymerase 1 (PARP1) at 1:1000 antibody (Bio-Rad Cat# MCA1522G);
ɣH2Ax at 1:1000 (Millipore, Cat#. 05-636, Clone JBW301);
Anti-poly-ADP-ribose binding reagent fused to rabbit Fc tag at 1: 400 (Millipore, Cat# MABE1016);
dsDNA Marker Antibody at 1:2000 (, Santa Cruz Biotechnology, Cat# sc-58749);
S9.6 at 1:100 (Millipore, Cat# MABE1095);
HUWE1 antibody (BETHYL, Cat# A300-486A);
Rabbit monoclonal (anti-c16orf72) HAPSTR1 antibody at 1:1000 from Nicholas Lakin Lab (Sharma et al., 2023);
Rabbit anti-HA Tag Polyclonal Antibody at 1:1000 (Invitrogen Cat# 71-5500, SG77);
β-Actin at 1:5000 (Santa Cruz Biotechnology, Cat# sc-47778);
Vinculin at 1:1000 (Cell Signalling Technology Europe BV, Cat# 4650).
The polyclonal antibody against c16orf72/HAPSTR1 was raised in rabbit using a 16-residue peptide and was generated by Eurogenetics.
Secondary Antibodies
Alexa FluorTM 488 goat anti-mouse IgG (H+l) at 1:500 (Invitrogen Cat# A11029);
Alexa FluorTM 488 chicken anti-rat IgG (H+l) at 1:500 (Invitrogen Cat# A21470);
Alexa FluorTM PLUS 488 donkey anti-rabbit IgG (H+l) at 1:500 (Invitrogen Cat# A32790),
Alexa FluorTM 555 goat anti-rat IgG (H+l) at 1:500 (Invitrogen Cat# A21434);
Goat anti-mouse Alexa FluorTM 555 pAb at 1:500 (Abcam, Cat# ab150114),
Polyclonal Goat anti-Mouse immunoglobulins/HRP at 1:10,000 for western blot, 1:2000 for dsDNA and 1:500 for G4 (Agilent Dako, Cat# P0447);
Polyclonal Goat anti-Rabbit immunoglobulins/HRP at 1:10,000 (Agilent Dako, Cat# P0448);
Polyclonal Swine anti-Rabbit immunoglobulins/TRITC at 1:10,000 (Agilent Dako, Cat# R0156);
Polyclonal Rabbit anti-Mouse immunoglobulins/FITC at 1:10,000 (Agilent Dako, Cat# F0313)
Rabbit monoclonal PCNA at 1:1000 (D3H8P XP, Cell signalling)
Rabbit polyclonal Anti-TERF1 at 1:1000 (HPA048379, Sigma Aldrich)

Validation

BD Biosciences, Cat# 347580 is validated by BD Biosciences in Gratzner HG. Monoclonal antibody to 5-bromo and 5-iododeoxyuridine: A new reagent for detection of DNA replication. Science. 1982;218:474.
Abcam Cat# ab6326, clone BU1 / 75 (ICR1): https://www.abcam.com/en-us/products/primary-antibodies/brdu-antibody-bu1-75-icr1-proliferation-marker-ab6326
Merck Life Science Cat# MABE1126, clone 1H6: https://www.merckmillipore.com/GB/en/product/Anti-DNA-G-quadruplex-G4-Antibody-clone-1H6,MM_NF-MABE1126
Bio-Rad Cat# MCA1522G: https://www.bio-rad-antibodies.com/monoclonal/human-poly-adp-ribose-polymerase-1-antibody-a6-4-12-mca1522.html
Millipore, Cat#. 05-636, Clone JBW301: https://www.merckmillipore.com/GB/en/product/Anti-phospho-Histone-H2A.X-Ser139-Antibody-clone-JBW301,MM_NF-05-636
Millipore, Cat# MABE1016: https://www.merckmillipore.com/GB/en/product/Anti-pan-ADP-ribose-binding-reagent,MM_NF-MABE1016
Santa Cruz Biotechnology, Cat# sc-58749: https://www.scbt.com/p/ds-dna-marker-antibody-hyb331-01
Millipore, Cat# MABE1095: https://www.merckmillipore.com/GB/en/product/Anti-DNA-RNA-Hybrid-Antibody-clone-S9.6,MM_NF-MABE1095
BETHYL, Cat# A300-486A: https://www.fortislife.com/products/primary-antibodies/rabbit-anti-lasu1-ureb1-antibody/BETHYL-A300-486
Invitrogen Cat# 71-5500, SG77: https://www.thermofisher.com/antibody/product/HA-Tag-Antibody-clone-SG77-Polyclonal/71-5500
Santa Cruz Biotechnology, Cat# sc-47778: https://www.scbt.com/p/beta-actin-antibody-c4
Cell Signalling Technology Europe BV, Cat# 4650: https://www.cellsignal.com/products/primary-antibodies/vinculin-antibody/4650
Invitrogen Cat# A11029: https://www.thermofisher.com/antibody/product/Goat-anti-Mouse-IgG-H-L-Highly-Cross-Adsorbed-Secondary-Antibody-Polyclonal/A-11029
Invitrogen Cat# A21470: https://www.thermofisher.com/antibody/product/Chicken-anti-Rat-IgG-H-L-Cross-Adsorbed-Secondary-Antibody-Polyclonal/A-21470
Invitrogen Cat# A32790: https://www.thermofisher.com/antibody/product/Donkey-anti-Rabbit-IgG-H-L-Highly-Cross-Adsorbed-Secondary-Antibody-Polyclonal/A32790
Invitrogen Cat# A21434: https://www.thermofisher.com/antibody/product/Goat-anti-Rat-IgG-H-L-Cross-Adsorbed-Secondary-Antibody-Polyclonal/A-21434
Abcam, Cat# ab150114: https://www.abcam.com/en-us/products/secondary-antibodies/goat-mouse-igg-h-l-alexa-fluor-555-ab150114
Agilent Dako, Cat# P0447: https://www.agilent.com/store/productDetail.jsp?catalogId=P044801-2
Agilent Dako, Cat# R0156: https://www.citeab.com/antibodies/3288355-r0156-rabbit-immunoglobulins (Note: supplier website doesn't show this product now)
Agilent Dako, Cat# F0313: https://www.agilent.com/store/productDetail.jsp?catalogId=F031302-2
The C16orf72/HAPSTR1 antibody raised against 16-residue peptide and generated by Eurogenetics was validated by western blot in Sharma et al, (2023), Nature Commun. 14, 7784 in supplementary figure 1b.

Eukaryotic cell lines

Policy information about cell lines and Sex and Gender in Research

Cell line source(s)	All cell lines have been described previously (Ronson et al, (2018), Nature Commun. 9,746) and in Sharma et al, (2023), Nature Commun. 14, 7784. Nomenclature for the cell line from c16orf72Δ .2 cells to HAPSTR1Δ .2 and cell line c16orf72Δ .3 have been changed o HAPSTR1Δ .3
Authentication	All cell lines have been described and authenticated previously (Ronson et al, (2018), Nature Commun. 9,746) and in Sharma et al, (2023), Nature Commun. 14, 7784
Mycoplasma contamination	All cell lines tested negative for mycoplasma infection
Commonly misidentified lines (See ICLAC register)	No commonly misidentified lines were used in this study

Plants

Seed stocks	Not Applicable
Novel plant genotypes	Not Applicable
Authentication	Not Applicable
