# Supplementary material for: G-quadruplex homeostasis is a determinant of PARP inhibitor toxicity in BRCA2-deficient cells
Source: Nat Commun. 2026 Jul 3;17:8275. doi: 10.1038/s41467-026-75083-9 (PMC13470221; doi:10.1038/s41467-026-75083-9)
Supplement: Supplementary file 3 [file 41467_2026_75083_MOESM3_ESM.pdf]

nature portfolio

Peer Review File

G-quadruplex homeostasis is a determinant of PARP inhibitor toxicity in BRCA2-deficient cells

Corresponding Author: Professor Nicholas Lakin

This file contains all reviewer reports in order by version, followed by all author rebuttals in order by version.

Attachments originally included by the reviewers as part of their assessment can be found at the end of this file.

Version 0:

Reviewer comments:

Reviewer #1

(Remarks to the Author)
This study investigates how PARP inhibitors exert their cytotoxicity, particularly in BRCA2-deficient cells and what determines cellular responsiveness to those clinically relevant anticancer drugs. Using U2OS culture cells, the authors uncover a partly redundant role of HAPSTR1, PARP1/PARP2, and BRCA2 in suppression of genomic G-quadruplex structures, and find that de-regulation of the G4 suppression mechanisms associates with PARP inhibitor cytotoxicity. DNA fiber assays and proximity-ligation assays suggest that de-regulation of the redundant G4 suppression mechanisms correlates with DNA replication stalling at the G4 structures and subsequent ssDNA gap accumulation that is mediated by PRIMPOL, which ultimately leads to genomic instability and cell growth defects. These observations are further supported by overexpression of HAPSTR1 or upregulation of the protein through disruption of its negative regulator HUWE1 that leads to suppression of G4 structures in BRCA2-deficient cells and confers cellular resistance to PARP inhibitors.
Overall, the experiments are well executed, the data are of high interest, and the manuscript is written with clarity. This study provides novel insights into 1) the mechanism of ssDNA gap formation during DNA replication, 2) how G4 structures contribute to genome instability, and 3) the functions of key genome maintenance factors, including the tumor suppressor BRCA2, in G4 regulation. However, the some of the experiments that lead to the conclusions that HAPSTR1, PARP1/PARP2, and BRCA2 contribute to G4 resolution and prevent ssDNA accumulation should be improved and the significance of these parallel pathways need to be made more clear. Therefore, I would recommend this manuscript for publication once the authors can address the specific questions and the detailed comments below.

Major points:
1. Lines 116-118: Because PDS treatment in HAPSTR1 KO cells also induces accumulation of R-loops (Fig. 1a), the authors should clarify whether this PDS sensitivity results from G4 accumulation rather than R-loop accumulation by, for example, transiently expressing RNaseH. This would also strengthen their conclusions in the later part regarding the link between G4-associated ssDNA gaps (Fig. 3f) and cell survival and improve an overall readability of this manuscript.
In addiiton, I could not find the information for how (much) the authors expressed FLAG-HA-HAPSTR1 in the HAPSTR1 KO cells in the manuscript. The expression levels of FALG-HA-HAPSTR1 compared to the endogenous one in WT U2OS cells should be indicated for interpretation of the data.

2. Lines 133-135: Could the authors provide a potential mechanism by which HAPSTR1 suppresses G4 structures and subsequent R-loops? There is a general lack in explanation what type of protein HAPSTR1 is and how it could act. Could the authors at least introduce the type of protein HAPSTR1 is in the introduction, and hypothesize on its role in G4/R-loop suppression in the discussion? For the same reason, could the authors provide a model(s) on how PARP1/2 and BRCA2 promote G4 resolution? Particularly, does the BRCA2 role in G4 suppression associate with its HR mediator function? In other words, does BRCA1 knockdown or RAD51 inhibition similarly leads to G4 accumulation?

3. Lines 146-156: I am not convinced with DNA damage induction in PDS-treated HAPSTR1 KO cells. First, although gH2AX is used as a DSB maker in this study, the phosphorylation of H2AX is mediated by ATM or ATR, thereby can be induced upon replication stress independently of DNA damage induction. To examine this more directly, a proper DNA double-strand break (DSB) marker such as 53BP1 foci should be used in HAPSTR1 KO cells in the presence/absence of PDS.

Second, because PDS-treated HAPSTR1 KO cells also upregulate R-loops that can stall DNA replication (PMID: 34494544), the causal of elevated gH2AX levels is unclear from the current data. To clarify this point, please assess DNA damage induction in PDS-treated HAPSTR1 KO cells following transient expression of RNaseH to reduce R-loop levels.

4. Lines 160-168: The enhanced sensitivity to PDS in olaparib-terated HAPSTR KO cells can be explained by at least two additional mechanisms, which are not mentioned in the manuscript. First, this sensitivity can be caused by accumulation of two independent genotoxic structures, PDS-stablized G4 structures and transcription-coupled R-loops that can be enhanced in HAPSTR KO cells according to the authors' previous report (PMID: 37591890). Second, because PDS is a potent DSB inducer, the sensitivity to PDS on top of olaparib treatment can be simiply exlained by a potential DSB repair defect in HAPSTR KO cells. Thus, the mechanism underlying the sensitivity should be better clarified in the manuscript. For example, could the authors investigate whether a transcription inhibitor alleviates the PDS sensitivity and whether HAPSTR1 is involved in repair of DNA double strand breaks (by examining sensitivity to IR, cisplatin, and camptothecin)?

5. Lines 176-181: A role for HAPSTR1 in suppressing G4-replication conflicts is poorly substantiated and should be reinfored based on the following reasons: First, the resolution of the DNA fibre analysis combined with G4 co-staining (Supplementary Figure 2) is too low to conclude replication fork stalling at G4 sites in HAPSTR1 KO cells. Could the authors verify this using an alternative method with better resolution such as a PLA assay between G4 and DNA replication machinery or single-molecule localization microscopy as reported previously (PMID: 33953191)? A rescue condition is required in these experiments. Second, as mentioned in point 3 above, 53BP1 is more suitable as a DSB marker (Figure 2c).

6. Lines 205-207: 'these findings support a model in which HAPSTR1 and PARP1/PARP2 function in parallel pathways to recognize and suppress G4 accumulation during DNA replication.'

Although PARP1 binding to G4s and it's role for G4 suppression in a distinct HAPSTR1-dependent mechanism are convincing, whether the PARP1-mediated G4 suppression occurs during DNA replication and whether PARP2 is involved in the mechanism are not addressed in the manscript. The authros should at least investigate whether the PARP1-mediated mechanism acts in S phase. Also for better readability, could the authors add discussion about whether G4 resolution by PARP1/PARP2 and HAPSTR1 occurs befire or after G4-replicaiton collision?

7. Lines 228-230: An unclear but important point in this study is whether cell toxicity arises from ssDNA accumulation or G4 accumulation. To address this, could the authors examine whther PRIMPOL depletion alleviate cell survival upon G4 accumulation, such as in HAPSTR1 KO cells treated with PDS?

8. Lines 278-285: Given the BRCA2 role in protecting replication forks, ssDNA signals could result from over-resection of replication forks, raising a possibility that ssDNA accumulation in BRCA2 KD cells is not directly linked to dysregulated G4s. To substantiate the link between ssDNA and stablized G4 strctures, it would be better to characterize the ssDNA accumulation in BRCA2 knockdown cells. Could the authors investigate whether the increased ssDNA gaps in HAPSTR1 KO cells following BRCA2 depletion still associate with G4s by PLA between G4 and ssDNA?
In addition, their interpretation that 'HAPSTR1, BRCA2, and PARP1/PARP2 operate through distinct pathways to regulate G4 levels and that deregulation of these processes underpins PARP inhibitor toxicity to cells' is not fully supported by their experiments. At least the authors should verify the elevated G4 levels in HAPSTR1 KO cells following BRCA2 depletion in the presence/absence of a PARP inhibitor.

9. Lines 295-299: To what extent G4 structures contribute to ssDNA gap formation is unclear. To address this crucial point, could the authors please analyze ssDNA gaps in HAPSTR1-overexpressing cells following BRCA2 depletion and/or PARP inhibitor treatment?

10. Same point: The direct link between olaparib sensitivity in BRCA2 depleted cells and G4 accumulation is not sufficiently demonstrated. If G4 accumulation is the major causal of olaparib sensitivity in BRCA2-depleted cells, HAPSTR1 overexpression would alleviate PDS-sensitivity in BRCA2-depleted U2OS cells (for example shown in Figure 4b). If the overexpression similarly would similarly rescue a sensitivity towards other genotoxic reagents unrelated to G4 accumulation, such as cisplatin and camptothecin, it would suggest the rescue stems simply from compensating DSB repair capacity in the BRCA2-depleted cells. Could the authors investigate this point to clarify what inhibits cell growth in olaparib treated BRCA2 depleted cells?

Minor points:
1. Line 80: HLTF is not a DNA helicase, it is a DNA translocase that has not been directly shown to resolve G4 structures.

2. Line 108: 'no significant increase in R-loop accumulation was apparent in untreated HAPSTR1 KO cells relative to parental U2OS cells (Figure 1a).' This is counterintuitive especially after reading the statement in line 101 that 'HAPSTR1 acts in a pathway parallel to PARP1/PARP2 to supress R-loop accumulation and PARP inhibitor toxicity'.

Does this result suggest that HAPSTR1 and PARP1/2 suppress R-loops in a completely redundant manner or that HAPSTR1 has a backup role when PARP1/2 is defective? Or does this just suggest that HAPSTR1 is involved in G4-induced R-loop formation?

3. Also Figure 1a: A recent study showed that S9.6 shows non-specific binding towards dsRNA molecules in cells (PMID: 34232287). Signal specificity of S9.6 needs to be confirmed by showing it goes back to cakground levels upon RNaseH

treatment or by utilizing a different approach such as GFP-dRNH1 characterized in recent studies (PMID: 34232287, PMID: 36544021).

4. Line 104: ‘Therefore, we considered whether HAPSTR1 participates in G4 regulation and if disruption of this mechanism is the cause of R-loop accumulation in HAPSTR1 KO cells.’ This would be hard to follow for the readers outside the field because no relationship between G4- and R-loop structures is introduced. The introduction should include a more detailed summary of interaction of both structures. Likewise, please provide more extensive introduction for the HAPSTR1 protein for better readability.

5. Lines 173-176: Given the recent studies showing tight coupling of G4 and R-loop structures (PMID: 30591567, PMID: 34792172, PMID: 40504899), one can expect that the G4 upregulation in EdU-positive HAPSTR1 KO cells is accompanied by R-loop accumulation. Have the authors measured R-loop signals in the condition (Figure 2b)? This is important because the authors conclude involvement of HAPSTR1 in G4 resolution based on the unchanged R-loop singals in HAPSTR KO cells observed in Figure 1a.

6. What is the difference between Figure 1d and 2c?

7. Line 187: ‘HAPSTR1 and PARP1/PARP2 operate in parallel pathways to restrain G4 formation’. One can argue that these two pathways also resolve G4 structures but not restrain G4 formation. This sentence can be misread and needs clarification.

8. Lines 198-205: Olaparib tethers PARP1 to G4s thereby blocks HAPSTR1 binding to the G4s. Although these observations suggest dominant-negative action of Olaparib for inhibition of G4 suppression, Figure 2a and 2d challenge this possibility. Please provide explanation for this.

9. Lines 228-230: The rescue experiments for PRIMPOL-depleted cells are currently missing. Could the authors conduct the rescue this phenotype by expressing WT or a catalytically dead mutant, or tone down the statement on this PRIMPOL function?

10. Line 337: ‘In the case of elevated R-loops in HAPSTR1-deficient cells we conclude that R-loop accumulation is downstream of G4s based on the following observations’. Can the author speculate the mechanism how this downstream R-loop accumulation is mediated?

11. Line 356: ‘G4 structures are potent barriers to replication forks in vitro73, 74, 75 and in cells76.’ Direct in vitro evidence has been reported only recently (PMID: 34559566, PMID: 34494544, PMID: 33953191).

12. In Figure 5d, the pull-down experiment does not show HAMSTR1 ubiquitylation because it is possible that HAPSTR1 is co-precipitated by binding to another ubiquitylated protein. Could the authors validate polyubiquitination of HAPSTR1 by a reciprocal pull-down experiment, such as immunoprecipitation of HAPSTR1 followed by ubiquitin blot?

Reviewer #2

(Remarks to the Author)
I co-reviewed this manuscript with one of the reviewers who provided the listed reports. This is part of the Nature Communications initiative to facilitate training in peer review and to provide appropriate recognition for Early Career Researchers who co-review manuscripts.

Reviewer #3

(Remarks to the Author)
Comments on "G-quadruplex homeostasis is a key determinant of PARP inhibitor toxicity in BRCA2-deficient cells"

In this manuscript, the authors report that depletion of HAPSTR1 sensitizes cells to PARP inhibitors, focusing on the deregulation of G-quadruplex (G4) structures as a key determinant of PARPi toxicity, particularly in BRCA2-deficient cells. The authors posit that HAPSTR1, a substrate of the E3 ligase HUWE1 involved in pleiotropic stress responses, acts as a suppressor of G4 accumulation during DNA replication. They further argue that loss of HAPSTR1 in U2OS ALT cells results in increased replication stress, single-stranded DNA gaps, and heightened sensitivity to PARPi. Additionally, the authors propose that HAPSTR1 and BRCA2 function in parallel, independently, yet synergistically, influencing cellular responses to PARPi. They also suggest that HUWE1-dependent degradation of HAPSTR1 modulates G4 homeostasis and contributes to PARPi resistance in BRCA2-deficient contexts.
While the work has the potential impact in clinical application, the manuscript currently appears mostly descriptive and preliminary in its current form. It lacks specific mechanism, likely due to pleiotropic effects following HAPSTR1 depletion, which are not sufficiently dissected. Several interpretations in the manuscript are overextended or inconsistent with previous literature. Multiple facets discussed require clarification and additional mechanistic validation before this work can be considered. Below are the major and minor concerns.

Major Comments:

The primary concern is the overly descriptive nature of the study, which touches upon many aspects of DNA damage, replication stress, and synthetic lethality without extending beyond existing knowledge. The repeated use of terms such as "deregulation of G4," "suppression of G4," or "tolerance of G4" lacks clarification, particularly regarding the basis of G4 regulation.

The differentiation between types of G4 structures-telomeric versus promoter G4s is crucial. It has been shown that telomeric G4s, which form during lagging strand synthesis and interconvert to remodel stalled forks, are dynamically regulated, with BRCA2 playing a key role in their control (Lee et al., Nature Commun. 2023, PMID: 38587189). The authors, however, refer to "G4 deregulation" without defining what constitutes "regulation" in this context. Clarification is essential for interpreting their findings.

The experimental system appears to investigate G4 formation in an asynchronous population of U2OS ALT cells, using the G4 stabilizer PDS without synchronization in S phase. Given that G4s serve physiological roles in transcription regulation and telomere maintenance-functions that are cell cycle-dependent-the authors should differentiate between replication-associated G4 structures and those involved in transcription. Treating G4s as merely "bad" structures is an oversimplification. The manuscript would benefit from a discussion on the physiological versus pathological roles of G4, including appropriate citations and mechanistic insights.

The role of HAPSTR1 in G4 homeostasis remains unclear. Although associations between HAPSTR1 and G4 structures are shown via antibody and PLA assays, the mechanistic basis-how HAPSTR1 directly influences G4 formation, stability, or resolution-is not addressed and should be elaborated.

The current title suggests a mechanistic focus on G4 homeostasis; however, the manuscript instead offers a broad overview of HAPSTR1 functions with only superficial discussion of G4 regulation. To justify the title, a clear mechanistic link must be established. At present, the data are insufficient to support a direct role for HAPSTR1 in G4 homeostasis.

Prior literature demonstrates that BRCA2 interacts directly with telomeric G4 structures to maintain replication (Lee et al., Nat Commun. 2022), and that BRCA2 deficiency prompts G4 accumulation and R-loop formation via disrupted G4 dynamics (Lee et al., Nucleic Acids Res. 2024). Further, Autjors have published previously that HAPSTR1 has been implicated in R-loop resolution via pathways involving BRCA1/Senataxin (Sharma et al., Nat Commun. 2023), which appear distinct from G4 regulation. Given these findings, it is unclear whether G4 accumulation in HAPSTR1-deficient cells is a direct consequence of G4 deregulation or an indirect effect secondary to unresolved R-loops or other secondary effects. Additional evidence is needed to elucidate the mechanistic interplay between HAPSTR1, G4 structures, and R-loops, and how these influence PARP inhibitor responses in BRCA2-null contexts.

Specific Comments:

Authors show that transfection of FLAG-HA-HAPSTR1 overcomes the effects of HASPTR1, particularly on G4 deregulation, in U2OS cells. This transfection will overexpress FLAG-HAPSTR1. The expression level, and the depletion level, is not shown in any figure or supplementary figures. Authors need to show the expression level: whether it is 1:1 or overexpression is needed.

The cell line used in this study, U2OS is ALT cancer cell line where telomere G4s are likely to be deregulated1 and depend on Break-induced Replication (BIR)2, 3, in addition to or more than HR4. Particularly, when BRCA2 is absent, cells adopt BIR for ALT5, 6. Authors are investigating on the effect of HAPSTR1 ablation in toxicity towards PARPi, also suggests the involvement of TLS. This reviewer asks whether there is cell line dependency, especially when it is reported that HAPSTR1 has pleiotrophiv effects depending on cell lines. More cell lines should be involved in the assay at least for some of the key experiments.

In page 5 and in Figure 1e in micronuclei assessment. Micronuclei are produced after chromosome mis-segregation. In the figure, U2OS cells semmed to form micronuclei more frequently than expected from many other labs, even in control. How does PDS cause the profuction of micronuclei and what is the percentage of micronuclei formation vs G4-positive micronuclei?

In page 5, the authors write that HAPSTR1 and PARP1/2 act in parallel pathways to facilitate cellular tolerance of G4 accumulation? This reviewer does not agree whether cells “tolerate” G4. Again, according to the study shown by Lee et al (Nature Comm. 2023), the dynamic interconversion of G4 underlies telomere replication homeostasis. It should be clear in the experiment whether it is the perturbation of the dynamic interconversion or other mechanims that may result in G4 accumulation. I have to reiterate that telomere G4 and G4 in transcription have distinct functions in distinct cell cycle phases. Authors really should discriminate the two crucial functions when referring to G4 regulation. And, properly cite the reports.

Page 6, authors and suggest that loss of HAPSTR1 would result in an increased dependence of cells on PARP1/2-mediated G4 regulation. This is one of the example of overinterpretation.

The references used for PARP1 binding to G4 need to be revised.

The study in HAPSTR1 and PARP1/2 in suppressing ssDNA gaps at G4 structures is puzzling. These studies in its own should be investigated rigorously and may be separated in an independent manuscript. In this manuscript, authors have continuously mixed replication and transcription effects by G4, by not synchronizing cells and mixing references. The study in ssDNA gaps is related to replication stage. In this line, it is quite puzzling that authors suggest, with weak evidence, the involvement of PRIMPOL and TLS in the repair of ssDNA gaps in HAPSTR1 KO cells. BRCA2 functions at the lagging strand telomere synthesis where G4 forms, but not in leading strand7, where PRIMPOl works. Therefore, the authors should investigate further on the mechanism of how these discrepancies can be resolved.

In page 9, the authors write that BRCA2-dependent increase in G4 levels was completely suppressed by the expression of HAPSTR1. Please provide the mechanism how. This statement is inconsistent with what was described as parallel pathways between BRCA and HAPSTR1 in PARPi sensitivity.

For all graph data measured from immunofluorescence (Figures 1a, b, d, e, Fig2. a-d, g, Fig. 3, 4, fig. 5a, h) please provide convinving image data, at least the representative staining). This is important since antibody to G4 and R-loop are all with background noises. Overall intensity and measuring specific foci would generate quite big differences. Overall, the data in graph do not seem convincing.

The G4 foci per nucleus do not seem convincing, as the untreated U2OS cells already have many (Fig. 1C), and that G4 in replication and for transcription are not differentiated throughout the scoring.
The survival data u Fig 4b seem to suggest that U2OS cells are quite resistant to siBRCA2 and/orsiBRCA2+HAPSTR1 depletion. Increasing concentration of PDS do not significantly affect the toxicity. Please explain.
Fig. 5a, What is the mechanism underlying the overcome of G4 foci per nucleus by HAPSTR1 overexpression, which was increased by depleting BRCA2?
The level of expression by transfection and depletion of HAPSTR1 should be provided by WB.
Figure 1b and 1c: The authors claim that G4 accumulation causes R-loop accumulation. However, in untreated HAPSTR1-deficient cells, G4 levels are elevated, whereas R-loop levels are not increased (Figure 1a). This discrepancy challenges the model that G4s drive R-loop formation under basal conditions. Please provide a clearer mechanistic explanation or hypothesis for why R-loops are not elevated despite G4 accumulation.
The description of HAPSTR1Δ.3 of Figure 2a is required in the manuscript.
The authors need to clarify the γH2AX intensity in HAPSTR1Δ.2 since HAPSTR1 depletion increased γH2AX in previous work (Sharma et al., Nat Commun, 2023). whereas there is no significant difference in Figure 1d and Figure 2c. In addition, the immunostaining of gamma-H2Ax should be analyzed by number of foci and the intensities, not the overall nuclear intensity.
Figure 2c seems to reconstitute the same results of Figure 1d (untreated).
The representative PLA image in Figure 2e (U2OS cells) does not reflect the quantified data (~30 foci) shown in the accompanying graph. The image should be replaced or revised to better represent the quantification.
Figure 1d: The authors state that DNA damage (γH2AX) is only elevated in S-phase cells (EdU+). However, in the graph, the EdU-negative cells in HAPSTR1-depleted conditions appear to show a modest increase in γH2AX after PDS treatment. Given this, I suggest increasing the number of biological replicates or providing statistical power analysis to support the claim that the difference is non-significant (ns).
Figure 2 (especially 2d and 2f): The authors propose that PARP1/2 and HAPSTR1 function in compensatory pathways to resolve G4s. The proximity ligation assay (PLA) in Figure 2f supports this, as PARP inhibition reduces HAPSTR1-G4 proximity (likely due to PARP1 trapping), while PARP1/2 KO restores it. However, in Figure 2d, G4 accumulation in HAPSTR1 KO cells treated with olaparib appears additive rather than compensatory, raising questions about the extent of functional redundancy. To solidify the claim of compensation, I strongly recommend including functional rescue experiments, such as G4 reporter assays or replication fork progression analysis, to determine whether one pathway (HAPSTR1 or PARP1/2) can substitute for the loss of the other in suppressing G4-related replication stress.
Typographical errors in Figure 1a “HAPSTR11Δ.2” and Figure 1c "HASPTR1" should be corrected to “HAPSTR1Δ.2” and "HAPSTR1" each.

References

1. Lee, J.J. et al. Disruption of G-quadruplex dynamicity by BRCA2 abrogation instigates phase separation and break-induced replication at telomeres. Nucleic Acids Res (2024).
2. In, S., Renck Nunes, P., Valador Fernandes, R. & Lingner, J. TERRA R-loops trigger a switch in telomere maintenance towards break-induced replication and PRIMPOL-dependent repair. EMBO J (2025).
3. Zhang, T. et al. Break-induced replication orchestrates resection-dependent template switching. Nature 619, 201-208 (2023).
4. Cho, N.W., Dilley, R.L., Lampson, M.A. & Greenberg, R.A. Interchromosomal homology searches drive directional ALT telomere movement and synapsis. Cell 159, 108-121 (2014).
5. Lee, J.J. et al. Disruption of G-quadruplex dynamicity by BRCA2 abrogation instigates phase separation and break-induced replication at telomeres. Nucleic Acids Res 52, 5756-5773 (2024).
6. Kwon, M.S. et al. Brca2 abrogation engages with the alternative lengthening of telomeres via break-induced replication. FEBS J 286, 1841-1858 (2019).
7. Min, J. et al. The breast cancer susceptibility gene BRCA2 is required for the maintenance of telomere homeostasis. J Biol Chem 287, 5091-5101 (2012).
8. Dilley, R.L. et al. Break-induced telomere synthesis underlies alternative telomere maintenance. Nature 539, 54-58 (2016).

Reviewer #4

(Remarks to the Author)
I co-reviewed this manuscript with one of the reviewers who provided the listed reports. This is part of the Nature Communications initiative to facilitate training in peer review and to provide appropriate recognition for Early Career Researchers who co-review manuscripts.

Reviewer #5

(Remarks to the Author)
I co-reviewed this manuscript with one of the reviewers who provided the listed reports. This is part of the Nature Communications initiative to facilitate training in peer review and to provide appropriate recognition for Early Career Researchers who co-review manuscripts.

Reviewer #6

(Remarks to the Author)
In this manuscript, Sharma et al identify a role for G4-quadruplexes in promoting PARP inhibitor sensitivity. The authors show that the stress response protein HAPSTR1 is important to suppress G4 structures. In its absence, PARP1 binds these structures resulting in its activation. Inhibition of PARP1 in HAPSTR1-knockout cells potentiates DNA damage accumulation and causes loss of viability, and this is exacerbated in BRCA2-deficient cells. The work provides a new mechanism regulating PARP inhibitor sensitivity in BRCA2-deficient cells. Overall, the manuscript is a strong contender for publication in Nature Communications, however a number of issues would first need to be resolved.

1. The first two figures paint a somewhat murky picture of the connection between G4 quadruplexes and R-loops. The authors state at the bottom of page 4 that their data “demonstrate that the elevated G4 structures observed in HAPSTR1 KO cells are … a cause of R-loop formation”. However, just a few lines above that, they state that “although G4 levels were significantly increased in untreated HAPSTR1 KO cells, R-loop levels were independent of HAPSTR1 status (figure 1c).” These statements appear to be conflicting. If G4 structures cause R-loops, would one not expect increased R-loops in HAPSTR1 knockout cells since these cells have more G4 structures? This is an important issue to be resolved, since the authors go on to re-state this claim on page 5: “..our findings that R-loop accumulation in HAPSTR1 KO cells is a consequence of G4 deregulation (figure 1a-c)”.

This issue is farther complicated by the fact that, in figure 2b the authors report a different result than in figure 1c: In the untreated (-EdU) samples there is no difference between G4 foci in control vs HAPSTR1 KO cells. In contrast, figure 1c, which appears to be an identical experiment, shows a statistically significant increase, which is described by the authors as mentioned above. Which result is true? How many times were these experiments performed independently, and is the increase reproducible or not?

2. If the G4 structures are indeed the cause of increased R-loops in HAPSTR1 KO cells, it would be important to investigate if the increased DNA damage observed in these cells (gH2AX foci, micronuclei) is caused by increased R-loops, or represent a more direct impact of G4 structures, without going through an R-loop intermediate. To address this, the authors could test if the DNA damage in PDS-treated HAPSTR1 knockout cells is suppressed by the transcription inhibitor (DRB) which blocks R-loop formation.

3. The micrographs in figure 1f are not convincing. The micronuclei shown are at the edge of the image and partially cut out. Wider field images need to be shown, to be able to judge the data.

4. Since the PARP1-G4 PLA experiment in figure 2e appears to be the first demonstration that PARP1 is recruited to G4 structures in cells, additional controls should be performed to confirm the specificity of the assay. For example, does treatment with the G4 stabilizer PDS increase the PARP1-G4 PLA foci?

5. The demonstration of ssDNA gaps in figures 3 and 4 is not convincing. This is another critical point that needs to be addressed. It is only through an S1 nuclease assay that ssDNA gap accumulation can be unequivocally shown. Without S1 nuclease treatment, regardless of labeling schemes, one cannot conclude that ssDNA gaps are measured. While gaps in HAPSTR1 KO cells are convincingly shown using S1 nuclease treatment in figure 3b, subsequent figure panels (3c, 3d, 3f, 4d) claim to be investigating ssDNA gaps without employing S1 nuclease assays. All these experiments need to be performed using S1 treatment, otherwise the authors cannot make the ssDNA claims presented in the manuscript. Moreover, it is customary to show the results of S1 nuclease fiber assays as IdU/CldU ratios, to normalize for differences in fork speed.

6. Figure 3g is misleadingly presented as ssDNA-G4 PLA. In fact, the authors treat cells with BrdU and perform a BrdU-G4 PLA. This needs to be clearly acknowledged, as other interpretations of these results can be possible. Next, the authors need to present proper single antibody controls for this experiment, as they showed for other PLA experiments (eg in figures 2e, 2g). Finally, to gain confidence that this assay indeed detects ssDNA gaps at G4 structures, additional controls are needed. For example, depletion of PrimPol can be performed to suppress ssDNA gap formation.

7. The G4 accumulation in BRCA2 and HAPSTR1 knockout cells seems additive rather than synergistic, arguing against a synthetic lethality phenotype. It would be informative to measure DNA damage (gH2AX foci) in these cells, with and without olaparib treatment.

8. Is the phenotype restricted to BRCA2, or is BRCA1 involved in a similar manner? Does BRCA1 depletion increase G4 and olaparib sensitivity of HAPSTR1 KO cells? Does HAPSTR1 overexpression rescue the olaparib sensitivity of BRCA1-deficient cells?
9. The result that HUWE1 depletion rescues the olaparib sensitivity of BRCA2-deficient cells was previously published (PMID: 33257658). This should be acknowledged.

10. I found the description of the data presentation in the figure legends somewhat confusing. For PLA and fiber experiments in Figure 3 (and other figures) the authors state that “Data represent the SEM from 3 independent experiments”. I presume they mean that “Data represent the mean+/- SEM” as they indicate for other figures (eg figure 2g). Even so, this is confusing because the data they show is not actually the means of the three experiments, but rather, if I understand correctly, they combine all individual measurements from the three experiments in one large dataset and they then calculate the mean and SEM for the whole dataset. This should be made clear. Moreover, if indeed three independent replicates were performed for these experiments, it would be informative to plot them separately (or to plot the mean values of each individual experiment

in a separate graph) in order to be able to assess the reproducibility of the result. This would be particularly important for results with small differences which may or may not be reproducible (see point 1 above).

Version 1:

Reviewer comments:

Reviewer #1

(Remarks to the Author)
The authors have performed a series of experiments that address many of my concerns. I have only one major remaining issue that should be addressed, as well as a few smaller points that can be addressed through textual changes. I would recommend this manuscript to be published on the condition they can address these points.

The major issue relates to the first point in my review were I asked the authors to show whether the PDS sensitivity (and the observed DNA damage) in HAPSTR1 deficient cells could also result from the accumulation of R-loops rather than G4's. The authors explained that technical limitations prevented them from using RNAseH expression to investigate this which we understand. Instead, they performed short DRB treatment to show independence of transcription. However, this data cannot rule out the possibility that R-loops are the determinant of the toxicity/damage. First, it is not shown that the short DRB treatment reduces R-loops. It needs to be demonstrated that R-loops are no longer elevated in PDS treated HAPSTR1 KO cells upon DRB treatment. This is a crucial control and especially important since it has been shown that R-loops can also form independently of ongoing transcription. This could be done by repeating the experiment in Figure 1a in the presence of DRB, and/or by performing G4-R-loop PLA assays in HAPSTR1 KO cells treated with PDS without or without DRB. This control for the DRB treatment is crucial to show G4 and not R-loop dependence and without it the authors cannot state that the elevated replication-associated DNA damage or PDS sensitivity in these cells is not attributable to R-loops.

In addition, there are some issues that can be addressed textually:
1) While the addition of the 53BP1 focus data is informative and important and follow a similar trend to gammaH2AX foci, EdU-negative HAPSTR1 KO cells exhibit enhanced 53BP1 foci (without accumulation of G4s, Figure 2b). This is surprising and should be explained.
2) The direct link between genome instability and G4 structures has not been demonstrated. For this, the authors should map DNA damage to G4 sites in the genome, however, this has not been done. Phrases like in the summary: 'genome instability at G4 sites' should be rephrased.
3) With respect to testing other DNA damaging agents in HAPSTR1 KO cells, it is good to know that this has been performed in previous work of the authors. It would help to briefly describe this in the introduction. However, the statement that these cells are proficient in HR cannot be concluded from the observation of intact HU-induced RAD51 foci because these are a marker of replication fork protection rather than for HR. If this is the evidence, this statement needs to be rephased.
4) The authors should mention in the method section that they are aware of the non-specific staining of the S9.6 antibody and refer to the manuscript in which they performed the controls for this.
5) The manuscript became rather lengthy after revision and lacks clarity here and there, especially in the discussion section. In addition, there are several sentences in the discussion that need rephrasing:
P13
1. "we report that stabilising G4s contributes to this phenotype to suppress replication-associated DNA damage and genome instability."

Unclear, stabilising G4s does not suppress replication-associated DNA damage.

2. "However, our observations that HAPSTR1 KO cells are not sensitive to DSB-inducing agents and are HR proficient would argue against this."

See Point 3 above.

3. "Overall, these data support a model in which G4s act as replication barriers that induce DNA damage signalling to γH2AX, with R-loops contributing secondary damage when present."

The link between G4s, replication stress, and DNA damage has been extensively characterised by the previous studies. Could the authors provide references? Whether R-loops contribute to secondary damage when present is not fully supported in this manuscript. This could simply result from stabilised G4s that cannot be unwound by DNA helicases. Please rephrase.

P.14
4. "Whether HAPSTR1 prevents G4 formation, directly remodels these structures, or recruits additional processing factors for their resolution remains unclear."

These possibilities are not mutually exclusive. "How HAPSTR1 restrains G4 formation remains unclear" would be better.

P.15

5. “We observe that ssDNA gaps in HAPSTR1 KO cells exposed to PARP inhibitors are induced by PRIMPOL-dependent repriming of replication forks that require TLS for repair”

This phenotype is not rescued, and therefore this sentence should be rephrased.

P.16.
6. “Notably, loss of BRCA2 results in elevated G4s at both telomeric and non-telomeric regions (Supplementary Figure S7c), suggesting a genome-wide increase in G4s that cause replication obstacles and DNA damage.”

This sentence suggests a role of BRCA2 before replication forks encounter G4s, which is unclear from the data in this manuscript. This sentence should be rephrased or substantiated by data and/or previous studies.

7. “Notably, ssDNA gaps induced by HAPSTR1 loss localize to G4 structures (Figure 3g).”

Figure 3g does not suggest that most ssDNA gaps are formed at G4 structures. ssDNA could be formed in the absence of G4s. Needs rephrasing.
Related to this point, whether elevated G4 levels in BRCA2 KD/HAPSTR1 KO cells is causal or consequence of ssDNA accumulation is unclear. This should be addressed textually.

8. “Our data point to increased replication fork instability, …..”

Change to : “G4-mediated replication fork instability, ….”

Reviewer #2

(Remarks to the Author)
I co-reviewed this manuscript with one of the reviewers who provided the listed reports. This is part of the Nature Communications initiative to facilitate training in peer review and to provide appropriate recognition for Early Career Researchers who co-review manuscripts.

Reviewer #3

(Remarks to the Author)
Review of “G-quadruplex homeostasis is a determinant of PARP inhibitor toxicity in BRCA2-deficient cells”

I appreciate the authors’ efforts to address the concerns raised during the previous round of review. The manuscript has improved through the inclusion of revised datasets and additional experiments. Nevertheless, several issues remain insufficiently clarified and could likely be addressed through feasible additional experiments or clearer presentation of the data.

1. Possible contribution of R-loops upon PDS treatment

Treatment with the G4 stabilizer PDS is central to this study. Because PDS treatment can also induce R-loop accumulation, particularly at replicating telomeres, it is important to clarify whether R-loops contribute to the observed phenotypes.

This could be assessed by transient expression of RNase H1. The authors state that PDS treatment cannot be prolonged for 72 hours as in the case of RNase H1 overexpression. Nevertheless, optimization of PDS treatment together with transient RNase H1 expression should at least be attempted. This would allow estimation of how much of the S9.6 signal represents bona fide R-loops and would help determine whether the consequences of G4 elevation are indeed independent of R-loops, as suggested.

In Supplementary Fig. S1d, the authors report an increase in S9.6 mean nuclear intensity. It is unclear how this value was quantified. Does it represent overall nuclear intensity? Because EdU labeling was included in this experiment to distinguish replication-associated signals, the quantification method should be clearly described. If overall nuclear intensity was measured, this metric may not accurately reflect discrete R-loop foci. The Methods and figure legends currently lack sufficient information.

2. Level of HAPSTR1 expression

The level of FLAG-HAPSTR1 expression across the experimental conditions should be more clearly documented. Although overexpression is shown in Fig. S8 and by RT-PCR, the data appear to indicate expression levels exceeding threefold, whereas the text suggests approximately twofold.

Because the study consistently compares U2OS, HAPSTR1Δ2, and HAPSTR1Δ2 + HAPSTR1 cells, western blot analysis including all three cell types should be provided in the supplementary information. In Fig. S8, the comparison appears limited to U2OS versus overexpression cells, without inclusion of the HAPSTR1Δ2 line.

3. Micronuclei analysis and antibody specificity

Antibodies detecting G4 structures and R-loops are known to exhibit background signals. In Fig. 1f, the authors show that the percentage of G4-positive micronuclei increases following PDS treatment. It would be informative to first present the overall level of micronuclei induction and then quantify the fraction of micronuclei that contain G4 signals.

In particular, the extent to which PDS treatment itself induces micronuclei, and whether this induction differs depending on the presence or absence of HAPSTR1, would provide a clearer interpretation of the data.

4. Telomeric versus non-telomeric G4 and R-loops

I remain unconvinced that the current data sufficiently demonstrate that the effects of HAPSTR1 depletion are independent of telomeres.

In Supplementary Figs. S4–S5 and Fig. S7, G4 immunostaining clearly increases throughout the nucleus, consistent with the authors' claim that DNA damage is not restricted to telomeres. However, telomeric foci can be defined and quantified (even though U2OS cells may show increased numbers due to aneuploidy). Therefore, the analyses in Fig. S1a and Fig. S7 would benefit from explicitly reporting the frequency of TRF1-positive telomeric G4 foci, with non-telomeric G4 foci analyzed separately.

Such analysis would more precisely define the contribution of telomeric versus non-telomeric events, which is important given the unique vulnerability of telomeric DNA to replication stress and DNA damage.

In the revised manuscript, the authors include RPE cells and argue that the role of HAPSTR1 in G4 homeostasis is independent of ALT status. I agree that the effect is not restricted to telomeres. However, the previous concern was not simply whether telomeres differ from other genomic regions, but whether HAPSTR1 might differentially influence replication fork stalling caused by G4 stabilization versus G4-related transcriptional processes.

The authors state in Supplementary Fig. S5 and in the text that G4 elevation in HAPSTR1 knockout cells treated with PARP inhibitors is not restricted to telomeres and is independent of ALT status. In my view, the data presented do not fully support the latter conclusion. Given the genome-wide effects of the G4 ligand demonstrated in the revised manuscript, it would be sufficient to state that the effects are not restricted to telomeres. The current data do not conclusively establish that the phenomenon is independent of ALT status.

Reviewer #4

(Remarks to the Author)
I co-reviewed this manuscript with one of the reviewers who provided the listed reports. This is part of the Nature Communications initiative to facilitate training in peer review and to provide appropriate recognition for Early Career Researchers who co-review manuscripts.

Reviewer #5

(Remarks to the Author)
I co-reviewed this manuscript with one of the reviewers who provided the listed reports. This is part of the Nature Communications initiative to facilitate training in peer review and to provide appropriate recognition for Early Career Researchers who co-review manuscripts.

Reviewer #6

(Remarks to the Author)
The authors made significantly efforts to address the reviewers' comments experimentally. The revised manuscript is greatly improved and satisfactorily addresses my concerns.

Version 2:

Reviewer comments:

Reviewer #1

(Remarks to the Author)
We are satisfied with the extra experiment that was performed based on our suggestion and also happy with the textual changes that were made to the manuscript . We now recommend publication and congratulate the authors with their interesting findings.

Reviewer #2

(Remarks to the Author)
I co-reviewed this manuscript with one of the reviewers who provided the listed reports. This is part of the Nature

Communications initiative to facilitate training in peer review and to provide appropriate recognition for Early Career Researchers who co-review manuscripts.

Reviewer #3

(Remarks to the Author)
Comments on the Revised Manuscript

Overall, the authors have made efforts to revise the manuscript in response to the reviewers' comments, and several aspects of the study have improved with the inclusion of additional data. I appreciate these efforts. However, in my assessment, a number of key concerns remain insufficiently addressed. Notably, several of these issues were shared with other reviewers, underscoring their importance for the interpretation of the study.

While the authors have responded extensively, in several instances the responses do not fully engage with the underlying concerns, which limits the extent to which the revisions resolve the central issues.

1. Assessment of R-loops using S9.6 antibody

A major concern relates to the use of the S9.6 antibody to detect R-loops, given its well-documented limitations in specificity and background. As also emphasized by other reviewers, more direct validation of R-loop signals is critical for supporting the study's conclusions.

I previously suggested the use of RNase H1 expression as a more direct approach to assess DNA:RNA hybrids. The authors instead rely on transcriptional inhibition (DRB) as an indirect strategy. While I understand the practical considerations outlined in the rebuttal, this approach does not fully resolve the concern, particularly given that R-loops can arise independently of ongoing transcription, as also noted by Reviewer 1.

Importantly, the revised manuscript still does not provide direct evidence demonstrating the specificity of the S9.6 signal (e.g., reduction upon RNase H1 expression), nor does it convincingly establish the extent to which R-loops contribute to the observed phenotypes. Therefore, this remains a significant limitation in the interpretation of the data.

2. Quantification of HAPSTR1 expression levels

I appreciate the authors' effort to quantify HAPSTR1 expression using qPCR. However, my original concern was not limited to the measurement method itself, but rather to the comparability of expression levels across experimental systems.

As also reflected in the reviewers' broader concerns regarding experimental interpretation, the study relies on comparisons between multiple cell lines and conditions. It remains unclear whether HAPSTR1 expression levels are quantitatively matched across these contexts. Without such normalization, it is difficult to determine whether the observed phenotypes reflect controlled biological differences or variability in expression levels.

Clarification of this point would strengthen the reliability of the conclusions.

3. Analysis of telomeric versus non-telomeric G4 signals (Figure S5a)

I previously suggested separating the analyses of telomeric and non-telomeric G4 signals. The rationale is that telomeric foci, although fewer, are discrete and quantifiable, whereas non-telomeric G4 signals are more diffuse and may not be directly comparable within the same analytical framework.

As also discussed by Reviewer 3 in relation to telomeric versus non-telomeric contributions, a more refined analysis would help clarify the biological interpretation. Combining these signals in a single graph may underestimate telomeric contributions while overrepresenting non-telomeric signals.

In the current revision, this issue does not appear to have been fully addressed. Instead, the authors emphasize that G4 elevation occurs in both ALT-positive and ALT-negative cells, concluding independence from ALT status. While this is an interesting observation, it does not directly resolve the concern regarding the quantitative treatment of telomeric versus non-telomeric G4s.

A more carefully separated analysis would be particularly valuable, given the potential importance of telomeric G4 regulation in this system.

Reviewer #4

(Remarks to the Author)
I co-reviewed this manuscript with one of the reviewers who provided the listed reports. This is part of the Nature

Communications initiative to facilitate training in peer review and to provide appropriate recognition for Early Career Researchers who co-review manuscripts.

Reviewer #5

(Remarks to the Author)
I co-reviewed this manuscript with one of the reviewers who provided the listed reports. This is part of the Nature Communications initiative to facilitate training in peer review and to provide appropriate recognition for Early Career Researchers who co-review manuscripts.

Open Access This Peer Review File is licensed under a Creative Commons Attribution 4.0 International License, which permits use, sharing, adaptation, distribution and reproduction in any medium or format, as long as you give appropriate credit to the original author(s) and the source, provide a link to the Creative Commons license, and indicate if changes were made.
In cases where reviewers are anonymous, credit should be given to 'Anonymous Referee' and the source.
The images or other third party material in this Peer Review File are included in the article's Creative Commons license, unless indicated otherwise in a credit line to the material. If material is not included in the article's Creative Commons license and your intended use is not permitted by statutory regulation or exceeds the permitted use, you will need to obtain permission directly from the copyright holder.
To view a copy of this license, visit https://creativecommons.org/licenses/by/4.0/

RESPONSE TO REVIEWERS

Although six reviews were submitted for this manuscript, three were co-reviews accompanying other reviewers' reports. Therefore, we only provide responses to the three reviewers who provided substantive comments, namely reviewers 1, 3, and 6.

REVIEWER 1

We thank this reviewer for their positive comments and considering that the experiments are well executed, the data are of high interest, and the manuscript is written with clarity. We are also grateful for the clear, polite and constructive nature of the comments. We provide our responses below.

Major comments

1. *Because PDS treatment in HAPSTR1 KO cells also induces accumulation of R-loops (Fig. 1a), the authors should clarify whether this PDS sensitivity results from G4 accumulation rather than R-loop accumulation by, for example, transiently expressing RNaseH.*

To achieve PDS sensitivity in our system, cells must be exposed to PDS for 72 hours. However, sustained expression of RNaseH over this period is toxic to cells. Similarly, while treatment with DRB could be used to assess the dependence of PDS toxicity on R-loops, exposure to this agent beyond 3–6 hours also results in significant cytotoxicity. These technical limitations make this experimental approach unfeasible. Nevertheless, the shorter DRB treatment window is suitable for determining whether PDS-induced DNA damage is transcription-dependent (see below). As these experiments specifically assess replication-associated DNA damage that underlies PDS sensitivity, we hope this reviewer agrees that this approach adequately addresses their point within the constraints of the system.

I could not find the information for how (much) the authors expressed FLAG-HA-HAPSTR1 in the HAPSTR1 KO cells in the manuscript. The expression levels of FALG-HA-HAPSTR1 compared to the endogenous one in WT U2OS cells should be indicated for interpretation of the data.

In the original manuscript, we included a western blot demonstrating HAPSTR1 expression levels in U2OS cells with or without recombinant FLAG-HA-HAPSTR1 expression (Supplementary Figure S4 in the original manuscript, now Supplementary Figure S8a). This confusion may have arisen from our labelling the FLAG-HA-HAPSTR1–expressing cells as "U2OS + HAPSTR1," which we have now corrected. We have also incorporated quantitative PCR analysis of mRNA levels in these cell lines, which shows an average 2-fold increase in total HAPSTR1 mRNA expression relative to parental U2OS cells (Supplementary Figure S8b).

2. *Lines 133-135: Could the authors provide a potential mechanism by which HAPSTR1 suppresses G4 structures and subsequent R-loops? There is a general lack in explanation what type of protein HAPSTR1 is and how it could act. Could the authors at least introduce the type of protein HAPSTR1 is in the introduction, and hypothesize on its role in G4/R-loop suppression in the discussion?*

As requested, we have included an introduction to HAPSTR1 in the introduction (page 3-4). We have also hypothesised regarding its role in G4/R-loop suppression in the discussion (page 13-14).

For the same reason, could the authors provide a model(s) on how PARP1/2 and BRCA2 promote G4 resolution? Particularly, does the BRCA2 role in G4 suppression associate with its HR mediator function? In other words, does BRCA1 knockdown or RAD51 inhibition similarly leads to G4 accumulation?

We have now significantly revised the discussion, including a section outlining how we could envisage PARP1/2 and BRCA2 promoting G4 resolution (page 14-17).

With regards to a broader role of HR in suppressing G4 structures, our preliminary data indicate a similar increase in nuclear G4 foci and sensitivity to PDS upon BRCA1 depletion. Unfortunately, given the scope of the experiments described here, in addition to the extent of revisions requested, we have been unable to investigate this further in the context of HAPSTR1 or PARP1/2 deficient backgrounds. Therefore, while we acknowledge that such experiments would be informative, we hope the reviewer understands we prefer to focus on BRCA2 and that a detailed investigation of other HR genes is beyond the scope of the current study.

3. *I am not convinced with DNA damage induction in PDS-treated HAPSTR1 KO cells. First, although gH2AX is used as a DSB maker in this study, the phosphorylation of H2AX is mediated by ATM or ATR, thereby can be induced upon replication stress independently of DNA damage induction. To examine this more directly, a proper DNA double-strand break (DSB) marker such as 53BP1 foci should be used in HAPSTR1 KO cells in the presence/absence of PDS.*

We completely agree that γH2AX is not a marker of DSBs but DNA damage more generally, including replication stress signalled by ATR. Given the link between G4s and replication stress, we specifically assessed γH2AX induction in the original manuscript and intentionally avoided using terminology that implied it is a marker of DSBs. Given the link to DNA replication, we would prefer to retain γH2AX as the primary indicator of DNA damage in these experiments. Nonetheless, we recognize the value of directly assessing DSBs via 53BP1 foci and have included these additional analyses in the revised manuscript (see below).

Second, because PDS-treated HAPSTR1 KO cells also upregulate R-loops that can stall DNA replication (PMID: 34494544), the causal of elevated gH2AX levels is unclear from the current data. To clarify this point, please assess DNA damage induction in PDS-treated HAPSTR1 KO cells following transient expression of RNaseH to reduce R-loop levels.

This question can be addressed either by expressing RNaseH or by inhibiting transcription using DRB. Given the need to perform these experiments in several genetic backgrounds, in addition to the use of DRB in other studies that assess PARP inhibitor toxicity (e.g., PMID: 38509368), we employed DRB in these experiments. The results show that DNA damage (γH2AX) in EdU-positive *HAPSTR1* KO cells is unaffected by DRB treatment (revised Figure 1d), indicating that the elevated replication-associated DNA damage in these cells is not attributable to R-loops. DSB formation, as indicated by 53BP1 foci, was slightly sensitive to DRB (Supplementary Figure S2d). We interpret these findings as evidence for distinct pools of DNA damage in PDS-treated cells, with early replication-associated γH2AX signalling occurring independently of transcription, whilst subsequent replication fork breakage is transcription-dependent. This has been discussed in the manuscript (Results, pages 5-6; Discussion, page13-14).

4. *The enhanced sensitivity to PDS in olaparib-terated HAPSTR KO cells can be explained by at least two additional mechanisms, which are not mentioned in the manuscript. First, this sensitivity can be caused by accumulation of two independent genotoxic structures, PDS-stablized G4 structures and transcription-coupled R-loops that can be enhanced in HAPSTR KO cells according to the authors' previous report (PMID: 37591890). Second, because PDS is a potent DSB inducer, the sensitivity to PDS on top of olaparib treatment can be simply explained by a potential DSB repair defect in HAPSTR KO cells. The mechanism underlying the sensitivity should be better clarified in the manuscript.*

For example, could the authors investigate whether a transcription inhibitor alleviates the PDS sensitivity…….

As described above, DRB or RNaseH expression is not compatible with clonogenic survival assays to assess PDS sensitivity. However, we have assessed the dependence of DNA damage induction in *HAPSTR1* KO cells on transcription and discuss these data in terms of whether G4s or R-loops are the causative DNA lesion in the revised manuscript (Results, pages 5-6; Discussion, page13-14).

……. *and whether HAPSTR1 is involved in repair of DNA double strand breaks (by examining sensitivity to IR, cisplatin, and camptothecin)?*

During our initial characterisation of *HAPSTR1* KO cells (PMID: 37591890) we assessed their sensitivity to a range of DNA-damaging agents. HAPSTR1-deficient cells were not sensitive to the radiomimetic phleomycin, and although we did not specifically test cisplatin, they were insensitive to mitomycin C (which similarly induces DNA inter-strand crosslinks). In addition, these cells remain HR-proficient, as indicated by intact HU-induced Rad51 foci formation. Taken together, these findings argue against a defect in DSB repair, and we now note this point in the discussion (page 13).

5. *A role for HAPSTR1 in suppressing G4-replication conflicts is poorly substantiated and should be reinforced based on the following reasons: First, the resolution of the DNA fibre analysis combined with G4 co-staining (Supplementary Figure 2) is too low to conclude replication fork stalling at G4 sites in HAPSTR1 KO cells. Could the authors verify this using an alternative method with better resolution such as a PLA assay between G4 and DNA replication machinery or single-molecule localization microscopy as reported previously (PMID: 33953191)? A rescue condition is required in these experiments. Second, as mentioned in point 3 above, 53BP1 is more suitable as a DSB marker (Figure 2c).*

We now include PLA data that clearly illustrates an increased proximity of the replication fork component PCNA with G4s in HAPSTR1 KO cells that is rescued by expression of recombinant HAPSTR1 (Figure 2c; page 7). This dataset replaces the original Figure 2c, as the previous figure primarily represented an independent repeat of a subset of the data shown in Figure 1d (see minor point 6 below).

As described above, we have now included data analysis 53BP1 as a DSB marker.

6. *Although PARP1 binding to G4s and it's role for G4 suppression in a distinct HAPSTR1-dependent mechanism are convincing, whether the PARP1-mediated G4 suppression occurs during DNA replication and whether PARP2 is involved in the mechanism are not addressed in the manuscript. The authors should at least investigate whether the PARP1-mediated mechanism acts in S phase.*

In our experience PARP1 and PARP2 exhibit partially redundant roles during DNA replication. Given that olaparib inhibits both PARP1 and PARP2, we therefore examined G4 levels in PARP1/2 knockout cells. As shown in Supplementary Figure S4b (page 7), similar to *HAPSTR1* KO cells, disruption of PARP1 and PARP2 led to increased G4 accumulation specifically in replicating cells.

Also for better readability, could the authors add discussion about whether G4 resolution by PARP1/PARP2 and HAPSTR1 occurs before or after G4-replicaiton collision?

We have discussed the potential roles of HAPSTR1 and PARP1/PARP2 in G4 resolution and whether this occurs before or after replication fork collision in the Discussion.

7. _An unclear but important point in this study is whether cell toxicity arises from ssDNA accumulation or G4 accumulation. To address this, could the authors examine whether PRIMPOL depletion alleviate cell survival upon G4 accumulation, such as in HAPSTR1 KO cells treated with PDS?_

We have now included these data in the revised manuscript (Figure 3d, page 9).

8. _Given the BRCA2 role in protecting replication forks, ssDNA signals could result from over-resection of replication forks, raising a possibility that ssDNA accumulation in BRCA2 KD cells is not directly linked to dysregulated G4s. To substantiate the link between ssDNA and stabilized G4 structures, it would be better to characterize the ssDNA accumulation in BRCA2 knockdown cells. Could the authors investigate whether the increased ssDNA gaps in HAPSTR1 KO cells following BRCA2 depletion still associate with G4s by PLA between G4 and ssDNA?_

In addition, their interpretation that 'HAPSTR1, BRCA2, and PARP1/PARP2 operate through distinct pathways to regulate G4 levels and that deregulation of these processes underpins PARP inhibitor toxicity to cells' is not fully supported by their experiments. At least the authors should verify the elevated G4 levels in HAPSTR1 KO cells following BRCA2 depletion in the presence/absence of a PARP inhibitor.

We thank the reviewer for raising the important point regarding ssDNA gap formation *versus* over-resected replication forks. We agree that the ssDNA assays used in the original manuscript could not distinguish between these structures. To address this limitation, we have now employed DNA fibre–based approaches that selectively measure either replication-associated ssDNA gaps (Figures 3b, c, e and 4e), or replication fork degradation (Figure 4g).

Collectively, these analyses support our initial conclusion that BRCA2 or HAPSTR1 suppress ssDNA gap accumulation, particularly following PARP inhibition. However, the revised data also revealed that combined disruption of HAPSTR1 and BRCA2 does not further increase ssDNA gap formation relative to either perturbation alone, indicating that these factors act within the same pathway to limit ssDNA gap accumulation (Figure 4e). This result was unexpected, particularly in light of the enhanced sensitivity to PARP inhibition when HAPSTR1 and BRCA2 are disrupted in combination (Figure 4d). Therefore, we shifted our focus to replication fork integrity and found that olaparib treatment caused pronounced replication fork instability in cells when BRCA2 and HAPSTR1 are disrupted in combination (Figure 4f and g). Together, these data indicate that HAPSTR1, BRCA2, and PARP1/PARP2 operate through distinct pathways to regulate G4 levels and that the toxicity of PARP inhibitors in the absence of both BRCA2 and HAPSTR1 is driven by replication fork instability rather than ssDNA gap formation.

With regard to the reviewer's specific suggestions, these findings introduce technical limitations for the proposed analyses. In particular, simultaneous disruption of HAPSTR1, BRCA2 and PARP1/2 results in extensive ssDNA accumulation due to replication fork degradation, making it difficult to distinguish *bona fide* ssDNA gaps from ssDNA generated by replication fork degradation. For this reason, the suggested PLA analysis of ssDNA-G4s in a BRCA2/HAPSTR1/PARP defective background would be difficult to interpret in terms of ssDNA gaps. Moreover, extensive fork degradation at G4s in these cells complicates reliable quantification of G4 structures themselves, as loss of fork integrity could lead to depletion of detectable G4 signals.

Nevertheless, we believe our data support the conclusion that ssDNA gaps accumulate at G4 structures in HAPSTR1-deficient cells following BRCA2 depletion, at least at a subset of

G4s. Specifically, our analyses demonstrate that loss of HAPSTR1 leads to the formation of PrimPol-dependent ssDNA gaps (Figure 3c), and that these localise to G4 structures (Figure 3g). Given that BRCA2 and HAPSTR1 function within the same pathway to suppress ssDNA gaps (Figure 4e), it follows that BRCA2 also contributes to the suppression of ssDNA gap formation at G4s. It is important to note, however, that BRCA2 depletion in *HAPSTR1* KO cells is additive in terms of elevated G4 levels. Together, these data suggest the existence of two distinct populations of G4 structures: one in which BRCA2 and HAPSTR1 function cooperatively to suppress ssDNA gap formation, and a second population in which G4 regulation by HAPSTR1 and BRCA2 occurs independently. These findings are described in the revised Results section (page 10-11) and their broader implications are considered in the Discussion.

9. *To what extent G4 structures contribute to ssDNA gap formation is unclear. To address this crucial point, could the authors please analyze ssDNA gaps in HAPSTR1-overexpressing cells following BRCA2 depletion and/or PARP inhibitor treatment?*

Consistent with the resistance to PARP inhibitors observed in BRCA2-depleted U2OS cells expressing FLAG–HA–HAPSTR1, we also detected a suppression of olaparib-induced S1 nuclease sensitive ssDNA gaps (Supplementary Figure S8d; page 12).

10. *If G4 accumulation is the major causal of olaparib sensitivity in BRCA2-depleted cells, HAPSTR1 overexpression would alleviate PDS-sensitivity in BRCA2-depleted U2OS cells (for example shown in Figure 4b). If the overexpression similarly would similarly rescue a sensitivity towards other genotoxic reagents unrelated to G4 accumulation, such as cisplatin and camptothecin, it would suggest the rescue stems simply from compensating DSB repair capacity in the BRCA2-depleted cells. Could the authors investigate this point to clarify what inhibits cell growth in olaparib treated BRCA2 depleted cells?*

As noted previously in our response to point 4, our initial characterisation of *HAPSTR1* KO cells demonstrated that HAPSTR1 does not function in DSB repair (PMID: 37591890). This argues against the possibility that the observed rescue of PARP inhibitor sensitivity reflects a general compensation of DSB repair capacity in BRCA2-depleted cells.

With respect to assessing PDS sensitivity, we agree that, in principle, this experiment could be informative. However, we believe that interpretation of such experiments would be challenging for several reasons. Firstly, PDS induces artificially high levels of G4 structures, which may overwhelm any compensatory effects arising from modest overexpression of a single factor such as HAPSTR1. Moreover, our data indicate that G4-associated stress arising from genetic disruption and that induced by G4-ligands are mechanistically distinct. Specifically, loss of HAPSTR1 leads to increased G4 accumulation in the absence of detectable R-loop formation and induces replication-associated γH2AX independently of transcription (Figure 1a, d). In contrast, PDS treatment induces R-loop formation and double-strand breaks, as indicated by 53BP1 foci, in a manner that is at least partially dependent on transcription (Figure 1a and Supplementary Figure S2d). Given these fundamental differences, failure of HAPSTR1 overexpression to rescue PDS sensitivity would be difficult to interpret and would not necessarily discount this as a mechanism of driving PARP inhibitor sensitivity in BRCA2-depleted cells. In light of these considerations, and given the breadth of additional data supporting a link between G4 accumulation and PARP inhibitor sensitivity, we hope the reviewer will agree that these experiments are not essential for the conclusions of the current study.

Minor points

1. *Line 80: HLTF is not a DNA helicase, it is a DNA translocase that has not been directly shown to resolve G4 structures.*

This has been corrected (page 3).

2. *<u>Line 108: 'no significant increase in R-loop accumulation was apparent in untreated HAPSTR1 KO cells relative to parental U2OS cells (Figure 1a).' This is counterintuitive especially after reading the statement in line 101 that 'HAPSTR1 acts in a pathway parallel to PARP1/PARP2 to supress R-loop accumulation and PARP inhibitor toxicity'. Does this result suggest that HAPSTR1 and PARP1/2 suppress R-loops in a completely redundant manner or that HAPSTR1 has a backup role when PARP1/2 is defective? Or does this just suggest that HAPSTR1 is involved in G4-induced R-loop formation?</u>*

We appreciate the reviewer's comment and have clarified this point in the text (page 4). The statement in line 108 was intended to emphasize that our findings are consistent with our previous report (PMID: 37591890), which showed that R-loop accumulation in *HAPSTR1*-deficient cells occurs only under HU-induced replication stress or following PARP inhibition, with no change in untreated cells. This distinction is important, as we observe elevated G4 levels and DNA damage in *HAPSTR1* KO cells even in the absence of genotoxic agents (Figures 1c, d and 2b), supporting the idea that R-loops and DNA damage arise as a consequence of G4 formation rather than the reverse. This hypothesis is further supported by the reviewer's suggested experiments assessing whether DNA damage is not transcription-dependent (see point 3). Collectively, these findings reinforce the model that HAPSTR1 and PARP1/2 function in parallel pathways to suppress G4 accumulation and maintain genome stability in the absence of exogenous stress, and we have discussed this hypothesis in detail in the Discussion section.

3. *<u>A recent study showed that S9.6 shows non-specific binding towards dsRNA molecules in cells (PMID: 34232287). Signal specificity of S9.6 needs to be confirmed by showing it goes back to background levels upon RNaseH treatment or by utilizing a different approach such as GFP-dRNH1.</u>*

We understand these concerns and established robust conditions for S9.6 staining in our previous study using U2OS and *HAPSTR1* KO cells, including demonstrating signal specificity by suppressing signals using RNaseH (PMID: 37591890).

4. '*<u>Therefore, we considered whether HAPSTR1 participates in G4 regulation and if disruption of this mechanism is the cause of R-loop accumulation in HAPSTR1 KO cells.' This would be hard to follow for the readers outside the field because no relationship between G4- and R-loop structures is introduced. The introduction should include a more detailed summary of interaction of both structures. Likewise, please provide more extensive introduction for the HAPSTR1 protein for better readability.</u>*

We have now expanded the introduction to better explain the relationship between G4s and R-loops, as well as to provide additional background on HAPSTR1 (pages 3-4).

5. *<u>Given the recent studies showing tight coupling of G4 and R-loop structures (PMID: 30591567, PMID: 34792172, PMID: 40504899), one can expect that the G4 upregulation in EdU-positive HAPSTR1 KO cells is accompanied by R-loop accumulation. Have the authors measured R-loop signals in the condition (Figure 2b)?</u>*

We now include these data and refer to them in the text (Supplementary Figure S1d; page 5).

6. *<u>What is the difference between Figure 1d and 2c?</u>*
The reviewer is correct in noting that Figure 2c reiterates a subcomponent of Figure 1d. However, these represented two independent experimental sets, each performed in triplicate, and therefore constituted distinct datasets. We originally included these data to

improve the narrative of this section by focusing on G4 levels in the absence of exogenous agents. However, we now recognize that this has led to confusion. Accordingly, we have removed these data and revised the text to reflect this change. In their place, we have incorporated an experiment suggested by this reviewer to assess replication fork stalling at G4 sites in *HAPSTR1* KO cells (Major point 5).

7. *Line 187: 'HAPSTR1 and PARP1/PARP2 operate in parallel pathways to restrain G4 formation'. One can argue that these two pathways also resolve G4 structures but not restrain G4 formation. This sentence can be misread and needs clarification.*

To clarify, we have amended this sentence to '…….suggesting that HAPSTR1 and PARP1/PARP2 operate in parallel pathways to suppress G4 formation either by restraining their formation, or promoting their resolution' (page 7, para 2).

8. *Lines 198-205: Olaparib tethers PARP1 to G4s thereby blocks HAPSTR1 binding to the G4s. Although these observations suggest dominant-negative action of Olaparib for inhibition of G4 suppression, Figure 2a and 2d challenge this possibility. Please provide explanation for this.*

These data suggest the presence of two G4 populations: one that is suppressed in a redundant manner by HAPSTR1 and PARP1/2, and another that is suppressed by PARP1 alone. This model explains why we observe an increase in nuclear G4 levels following both olaparib treatment and HAPSTR1 disruption, yet do not detect increased HAPSTR1 at G4s in response to PARP trapping by olaparib. Importantly, this model is still consistent with our conclusion that HAPSTR1 and PARP1/2 suppress G4 levels through parallel pathways. We have now clarified this point in the discussion (page 14-15).

9. *Lines 228-230: The rescue experiments for PRIMPOL-depleted cells are currently missing. Could the authors conduct the rescue this phenotype by expressing WT or a catalytically dead mutant, or tone down the statement on this PRIMPOL function?*

We understand that without the PRIMPOL catalytic dead controls, we cannot conclude that PRIMPOL repriming activity is required to generate ssDNA gaps in our experiments. As suggested, we have therefore toned down this statement to '…..suggesting a requirement for PRIMPOL to generate these structures' (page 9).

10. *Line 337: 'In the case of elevated R-loops in HAPSTR1-deficient cells we conclude that R-loop accumulation is downstream of G4s based on the following observations'. Can the author speculate the mechanism how this downstream R-loop accumulation is mediated?*

In light of the new data presented in Figure 1a, d and Supplementary Figure S2d, we have removed this section of the discussion and replaced this with a section discussing the link between transcription, R-loops and G4s in the absence of HAPSTR1 (page 13-14).

11. *Line 356: 'G4 structures are potent barriers to replication forks* in vitro[73, 74, 75] *and in cells76.' Direct in vitro evidence has been reported only recently (PMID: 34559566, PMID: 34494544, PMID: 33953191).*

We thank this reviewer for highlighting these reports and have included them in the revised manuscript.

12. *In Figure 5d, the pull-down experiment does not show HAPSTR1 ubiquitylation because it is possible that HAPSTR1 is co-precipitated by binding to another ubiquitylated protein. Could the authors validate polyubiquitination of HAPSTR1 by a reciprocal pull-down experiment, such as immunoprecipitation of HAPSTR1 followed by ubiquitin blot?*

We acknowledge that the TUBE pulldown of HAPSTR1 could potentially occur indirectly through another protein that interacts with HAPSTR1. However, previous studies have demonstrated that HAPSTR1 is a substrate of HUWE1 *in vivo* and *in vitro* (PMID: 37167062; PMID: 35776542). Together with our observations that HAPSTR1 undergoes HUWE1-dependent proteasomal degradation, we believe these findings support our conclusion that HAPSTR1 is indeed a substrate of HUWE1. Nonetheless, we recognize this possibility and have incorporated the appropriate caveats into the text (page 12).

REVIEWER 3
Major Comments
The primary concern is the overly descriptive nature of the study, which touches upon many aspects of DNA damage, replication stress, and synthetic lethality without extending beyond existing knowledge. The repeated use of terms such as "deregulation of G4," "suppression of G4," or "tolerance of G4" lacks clarification, particularly regarding the basis of G4 regulation.
With respect, we disagree with this assessment, in addition to the more general comment that the manuscript lacked specific mechanisms. We acknowledge that this study does not define the precise biochemical activities by which HAPSTR1 or BRCA2 resolve and/or restrain R-loop formation. However, this was not the primary objective of the work. Rather, the focus was to define the endogenous DNA lesions that arise in the absence of HAPSTR1 and/or BRCA2, the pathways governing their resolution, and how these processes become deregulated upon PARP inhibition to cause replication-associated DNA damage and cell death. Consistent with this broader mechanistic framework, all other reviewer highlighted the importance of this work. Reviewer 6 noted that it "*provides a new mechanism regulating PARP inhibitor sensitivity in BRCA2-deficient cells*" while Reviewer 1 commented that it "*provides novel insights into (1) ssDNA gap formation during DNA replication, (2) the contribution of G4 structures to genome instability, and (3) the functions of key genome maintenance factors, including the tumour suppressor BRCA2, in G4 regulation.*"

Nevertheless, we appreciate the reviewers' comments and address all specific concerns in detail below. We agree that the term "*deregulation of G4s*" could be considered vague and have therefore removed this phrasing from the revised manuscript, including the Summary. However, where appropriate and in the correct context, we have retained the terms "*suppression of G4s*" and "*tolerance of G4s*" as these are standard terms in the field. We have also included substantial new data that significantly expands our analysis of replication-associated phenotypes in HAPSTR1- and BRCA2-deficient backgrounds using appropriate assays. As suggested by the reviewer, we also assessed whether elevated G4 levels in these contexts are specific to telomeres and extended our analyses to additional ALT-negative cell lines. Furthermore, we strengthened our investigation of the relationship between G4s and R-loop formation to clarify the causative lesions underlying replication-associated DNA damage.

Collectively, these new analyses directly link G4s to R-loop formation and replication-associated DNA damage, replication fork stalling, PrimPol-dependent repriming, ssDNA gap formation, and under conditions of combined pathway disruption, replication fork degradation. Together, we believe these additions move the manuscript beyond correlative observations toward establishing mechanistic causality.

The differentiation between types of G4 structures-telomeric versus promoter G4s is crucial. It has been shown that telomeric G4s, which form during lagging strand synthesis and interconvert to remodel stalled forks, are dynamically regulated, with BRCA2 playing a key role in their control (Lee et al., Nature Commun. 2023, PMID: 38587189). The authors, however, refer to "G4 deregulation" without defining what constitutes "regulation" in this context. Clarification is essential for interpreting their findings.

We have now performed extensive analyses to determine whether the elevated G4 levels observed in our experiments are restricted to telomeres or reflect a more general genome-wide deregulation. These data clearly demonstrate that the increase is not limited to telomeres but also occurs at non-telomeric regions (Supplementary Figures S5a and S7c). In response to the reviewer's comment below, we additionally show that elevated G4 levels are present in an ALT-negative *HAPSTR1* KO cell line (RPE1), and that these levels are further increased either by PARP inhibition or by BRCA2 depletion (Supplementary Figures S5b and S7b). Taken together, we believe these findings support a model in which G4 formation is broadly deregulated, rather than being a telomere-specific event.

The experimental system appears to investigate G4 formation in an asynchronous population of U2OS ALT cells, using the G4 stabilizer PDS without synchronization in S phase. Given that G4s serve physiological roles in transcription regulation and telomere maintenance-functions that are cell cycle-dependent-the authors should differentiate between replication-associated G4 structures and those involved in transcription. Treating G4s as merely "bad" structures is an oversimplification. The manuscript would benefit from a discussion on the physiological versus pathological roles of G4, including appropriate citations and mechanistic insights.

We were surprised by this comment, as our original manuscript already included several experiments specifically designed to assess G4 formation during DNA replication. Whilst cell synchronisation (e.g. double-thymidine block) might seem an attractive approach, we avoided using these procedures as prolonged nucleotide depletion and/or replication fork stalling activates the S-phase checkpoint, induces replication stress, and generates DNA damage that complicate the interpretation of results. Similarly, FACS-based sorting can introduce additional cellular stress. For these reasons, we labelled replicating cells actively undergoing replication without perturbing cell-cycle progression by pulsing cells with EdU. This approach is now widely considered the least invasive and most reliable method for identifying S-phase cells.

Nevertheless, we fully agree that distinguishing replication-associated G4 events from other sources of G4 formation is important. In response, we have incorporated a significant amount of new experimental data that directly addresses this point:

- Figures 1, 2 and Supplementary Figure S2 include additional analyses of G4 formation and DNA damage specifically in EdU-positive replicating cells, in addition to replication fork stalling at G4s by PLA (Figure 2c).
- Figure 3 includes new DNA fibre assays quantifying the presence of ssDNA gaps at G4s.
- Figure 4 includes additional DNA fibre-based analyses assessing replication fork instability and nascent strand degradation, providing mechanistic evidence for how stabilized G4s impede fork progression.

Together, these experiments clearly demonstrate that the G4 structures observed in our system, and the downstream damage we quantify, arise during DNA replication and are processed through canonical replication-stress pathways.

In response to the reviewer's request to address the physiological versus pathological roles of G4 structures, the Introduction and the Discussion has now been expanded to consider these aspects in greater depth.

The role of HAPSTR1 in G4 homeostasis remains unclear. Although associations between HAPSTR1 and G4 structures are shown via antibody and PLA assays, the mechanistic

basis-how HAPSTR1 directly influences G4 formation, stability, or resolution-is not addressed and should be elaborated.

We acknowledge the reviewer's point that the precise biochemical activities through which HAPSTR1 regulates G4 structures remain to be fully defined. However, we reiterate that the central objective of this study is to elucidate the basis of how particular DNA lesions drive PARP-inhibitor toxicity in specific genetic contexts. Whilst worthwhile, we hope the reviewer will appreciate that a detailed biochemical dissection of the HAPSTR1-associated activities involved in regulating G4 structures lies beyond the scope of the present study.

Instead, in the present manuscript we focus on the downstream consequences of HAPSTR1 loss. We clearly show that the deregulation of G4 levels in the combined absence of HAPSTR1, PARP activity, and BRCA2 leads to an additive accumulation of G4s, which in turn form replication-fork barriers. Our mechanistic analyses centre on these downstream events, demonstrating that elevated G4 structures promote G4–replication conflicts and trigger PrimPol-dependent repriming to generate ssDNA gaps. Furthermore, we provide evidence that co-depletion of BRCA2 and HAPSTR1 induces PARP-dependent replication-fork degradation, ultimately resulting in cell toxicity.

Nevertheless, we appreciate the reviewer's request for more discussion on how HAPSTR1 may influence G4s and have expanded our discussion to elaborate on potential mechanisms and include a more detailed discussion elaborating on how the HAPSTR1-HUWE1 axis may regulate G4s (page 13-14).

The current title suggests a mechanistic focus on G4 homeostasis; however, the manuscript instead offers a broad overview of HAPSTR1 functions with only superficial discussion of G4 regulation. To justify the title, a clear mechanistic link must be established. At present, the data are insufficient to support a direct role for HAPSTR1 in G4 homeostasis.

As highlighted above, the primary focus of this manuscript is to define the DNA lesions whose deregulation in response to PARP inhibition results in toxicity in specific genetic backgrounds. Our intent with the title is to capture this overarching theme, rather than to imply a detailed mechanistic model of G4 regulation. While we appreciate the reviewer's concern, we believe the title accurately reflects the scope of the study and as such are reluctant to change it.

Prior literature demonstrates that BRCA2 interacts directly with telomeric G4 structures to maintain replication (Lee et al., Nat Commun. 2022), and that BRCA2 deficiency prompts G4 accumulation and R-loop formation via disrupted G4 dynamics (Lee et al., Nucleic Acids Res. 2024). Further, Autjors have published previously that HAPSTR1 has been implicated in R-loop resolution via pathways involving BRCA1/Senataxin (Sharma et al., Nat Commun. 2023), which appear distinct from G4 regulation. Given these findings, it is unclear whether G4 accumulation in HAPSTR1-deficient cells is a direct consequence of G4 deregulation or an indirect effect secondary to unresolved R-loops or other secondary effects. Additional evidence is needed to elucidate the mechanistic interplay between HAPSTR1, G4 structures, and R-loops, and how these influence PARP inhibitor responses in BRCA2-null contexts.

Please see the response above with regards the comment on BRCA2 and telomere G4 structures.

Regarding the comment on whether G4 accumulation in HAPSTR1-deficient cells arises directly from G4 deregulation or indirectly through unresolved R-loops, we clearly show in the original manuscript that G4 formation in *HAPSTR1* KO cells is independent of

transcription (Supplementary Figure S2a, b in the revised manuscript). Nevertheless, we appreciate the importance of distinguishing between G4- and R-loop–dependent mechanisms and have now incorporated additional experiments to address this point more directly. Specifically, we used the transcription inhibitor DRB to test whether the replication-associated DNA damage observed in *HAPSTR1* KO cells is driven by G4 stabilization or by R-loops. These experiments show that DNA damage in EdU-positive replicating *HAPSTR1* KO cells is unchanged upon DRB treatment (revised Figure 1d), indicating that the elevated replication-associated γH2AX signal in these cells is not attributable to R-loops. Interestingly, we find that double-strand break (DSB) formation, measured by 53BP1 foci, is sensitive to DRB, at least to some extent (Supplementary Fig. S2d).

We interpret these results as evidence for distinct pools of DNA damage in PDS-treated HAPSTR1 KO cells: an early, replication-associated γH2AX response that occurs independently of transcription, and a later step involving replication-fork breakage that appears transcription-dependent. These points are now discussed in detail in the revised manuscript (Results, page 5–6; Discussion, page 13-14).

Specific Comments
Authors show that transfection of FLAG-HA-HAPSTR1 overcomes the effects of HASPTR1, particularly on G4 deregulation, in U2OS cells. This transfection will overexpress FLAG-HAPSTR1. The expression level, and the depletion level, is not shown in any figure or supplementary figures. Authors need to show the expression level: whether it is 1:1 or overexpression is needed.

In the original manuscript, we included a western blot demonstrating HAPSTR1 expression levels in U2OS cells with or without recombinant FLAG-HA-HAPSTR1 expression (Supplementary Figure S4 in the original manuscript, now Supplementary Figure S8a). This confusion may have arisen from our labelling of the FLAG-HA-HAPSTR1–expressing cells as “U2OS + HAPSTR1,” which we have now corrected. We have also incorporated quantitative PCR analysis of mRNA levels in these cell lines, which shows an average 2-fold increase in total HAPSTR1 mRNA expression relative to parental U2OS cells.

The cell line used in this study, U2OS is ALT cancer cell line where telomere G4s are likely to be deregulated1 and depend on Break-induced Replication (BIR)2, 3, in addition to or more than HR4. Particularly, when BRCA2 is absent, cells adopt BIR for ALT5, 6. Authors are investigating on the effect of HAPSTR1 ablation in toxicity towards PARPi, also suggests the involvement of TLS. This reviewer asks whether there is cell line dependency, especially when it is reported that HAPSTR1 has pleiotrophiv effects depending on cell lines. More cell lines should be involved in the assay at least for some of the key experiments.

We include new data that replicate key observations in an ALT-negative cell line (RPE1; Supplementary Figures S1c, 5b and S7b, d). Unfortunately, RPE1 cells are particularly resistant to PDS, and we were therefore unable to assess PDS sensitivity in *HAPSTR1* KO cells in this background. However, we have evaluated G4 accumulation in these cells and reproduced all major findings including elevated G4 levels in *HAPSTR1* KO cells that is further increased upon PARP inhibition or BRCA2 depletion (Supplementary Figures S1c, 5b and S7b). Importantly, we also replicated the increased sensitivity of *HAPSTR1* KO cells depleted of BRCA2 to PARP inhibitors (Supplementary Figures S7d). Together, these results indicate that our observations are neither specific to U2OS cells nor dependent on ALT status.

In page 5 and in Figure 1e in micronuclei assessment. Micronuclei are produced after chromosome mis-segregation. In the figure, U2OS cells semmed to form micronuclei more frequently than expected from many other labs, even in control. How does PDS cause the

profuction of micronuclei and what is the percentage of micronuclei formation vs G4-positive micronuclei?
Micronuclei are well-established markers of replication stress (PMID: 33610905). Therefore, it is not surprising that G4 stabilisation by PDS results in micronuclei formation. We have amended the manuscript to clarify this point (page 6).

With respect to the frequency of micronuclei observed in this study, it is important to note that direct comparisons across studies are inadvisable due to differences in cell lines, staining protocols, antibody sensitivity, genotoxin exposure regimes, and other experimental variables. In our study, PDS treatment resulted in an average increase in micronuclei from 1.0% to 1.5% in parental U2OS cells and from 1.4% to 4.7% in HAPSTR1-deficient cells. If anything, these levels are slightly lower than those reported in other studies (e.g. PMID: 34139015), although these employed different PDS treatment protocols. The important point, however, is that in our experimental system we observe a statistically significant increase in micronuclei formation in *HAPSTR1* knockout cells compared with parental U2OS cells.

Regarding the question of "*the percentage of micronuclei versus G4-positive micronuclei*" we presume this to refers to the proportion of micronuclei that are positive for G4 structures. These data are presented in Figure 1F.

In page 5, the authors write that HAPSTR1 and PARP1/2 act in parallel pathways to facilitate cellular tolerance of G4 accumulation? This reviewer does not agree whether cells "tolerate" G4. Again, according to the study shown by Lee et al (Nature Comm. 2023), the dynamic interconversion of G4 underlies telomere replication homeostasis. It should be clear in the experiment whether it is the perturbation of the dynamic interconversion or other mechanims that may result in G4 accumulation. I have to reiterate that telomere G4 and G4 in transcription have distinct functions in distinct cell cycle phases. Authors really should discriminate the two crucial functions when referring to G4 regulation. And, properly cite the reports.

We thank the reviewer for this comment. We have revised the sentence in question to clarify that our data reflect cellular tolerance to the G4 ligand PDS, rather than tolerance to G4 structures themselves (page 6).

As outlined in our response to the reviewer's broader concerns, we now more explicitly distinguish telomeric G4s from G4s at other genomic loci. In our experiments, we observe a general increase in G4 signal that is not restricted to telomeres or any specific genomic region. Because this accumulation appears genome-wide, we do not interpret our findings as arising from perturbation of the telomere-specific dynamic interconversion of G4s described by Lee *et al.* (Nat. Commun. 2023). Instead, our results suggest a broader disruption of G4 homeostasis that is not telomere-selective.

Page 6, authors and suggest that loss of HAPSTR1 would result in an increased dependence of cells on PARP1/2-mediated G4 regulation. This is one of the example of overinterpretation.

We agree that stating 'This relationship predicts a potential compensatory mechanism whereby loss of HAPSTR1 would result in an increased dependence of cells on PARP1/PARP2-mediated G4 regulation' is misleading and we have toned down this statement. Instead, we believe our conclusion on page 6 of the original manuscript (lines 205-207; now page 8) that states 'Together, these findings support a model in which HAPSTR1 and PARP1/PARP2 function in parallel pathways to recognize and suppress G4 accumulation during DNA replication' is a more accurate conclusion. This is based on our epistasis analysis, designed to assess whether two factors operate within the same pathway, that shows:

a) Nuclear G4 accumulation in *HAPSTR1* KO cells treated with PARP inhibitors is additive relative to either untreated *HAPSTR1* KO cells or parental U2OS cells treated with PARP inhibitors.
b) Disruption of PARP signalling (either by inhibitors or *PARP1/2* gene disruption) increases HAPSTR1 association with G4s, and conversely, loss of HAPSTR1 elevates the accumulation of PAR at G4 structures.

The references used for PARP1 binding to G4 need to be revised.

We have revised these references (page 8).

The study in HAPSTR1 and PARP1/2 in suppressing ssDNA gaps at G4 structures is puzzling. These studies in its own should be investigated rigorously and may be separated in an independent manuscript. In this manuscript, authors have continuously mixed replication and transcription effects by G4, by not synchronizing cells and mixing references. The study in ssDNA gaps is related to replication stage. In this line, it is quite puzzling that authors suggest, with weak evidence, the involvement of PRIMPOL and TLS in the repair of ssDNA gaps in HAPSTR1 KO cells. BRCA2 functions at the lagging strand telomere synthesis where G4 forms, but not in leading strand7, where PRIMPOl works. Therefore, the authors should investigate further on the mechanism of how these discrepancies can be resolved.

We have now added new data to strengthen the link between suppression of G4 accumulation by PARP1, HAPSTR1 and BRCA2, and the formation of ssDNA gaps during DNA replication. We agree with the reviewer that the original ssDNA assay was limited in its ability to specifically detect replication-associated ssDNA gaps. To address this, we have now employed DNA fibre–based approaches that directly measure ssDNA gap accumulation during ongoing DNA replication (Figures 3b, c, e and 4e). In addition, we have used DNA fibre assays to assess replication fork degradation (Figure 4g), providing a more rigorous replication-specific analysis.

We were surprised by the reviewer's comment that the link between PrimPol, TLS and ssDNA gaps was weak, particularly given that these factors have been previously associated with the generation and processing of replication-associated ssDNA gaps, as cited in the original manuscript (PMID: 31676232; PMID: 34624216; PMID: 34645815; PMID: 33203852; PMID: 34508659). Nevertheless, to directly address this concern, we have now included new data that strengthens this section.

Using S1 nuclease DNA fibre assays, we show that depletion of PrimPol markedly suppresses ssDNA gap formation (Figure 3c), demonstrating that the replication-dependent ssDNA gaps observed in HAPSTR1 KO cells arise from PrimPol-dependent repriming events. In addition, newly added PLA analyses reveal that ssDNA formation at G4 structures is also dependent on PrimPol (Figure 3g), supporting a model in which PrimPol reprimes stalled replication forks at G4s, thereby generating ssDNA gaps. Importantly, we further demonstrate that PrimPol depletion suppresses the hypersensitivity of *HAPSTR1* KO cells to the G4-stabilising ligand PDS, (Figure 3d) indicating that PrimPol-dependent repriming and gap formation at stabilised G4s is a key source of toxicity in the absence of HAPSTR1.
With respect to TLS, we show that inhibition of Rev1 exacerbates both ssDNA gap accumulation and replication fork degradation in *HAPSTR1* KO cells (Figure 3e), indicating that TLS activity is required for proper processing of these gaps. Consistent with this, we demonstrate a requirement for Rev1 in maintaining the viability of *HAPSTR1* KO cells (Figure 3f).

Together, these new data provide direct mechanistic evidence linking PrimPol-dependent repriming, TLS-mediated gap processing, and ssDNA gap homeostasis in the context of HAPSTR1 loss.

In page 9, the authors write that BRCA2-dependent increase in G4 levels was completely suppressed by the expression of HAPSTR1. Please provide the mechanism how. This statement is inconsistent with what was described as parallel pathways between BRCA and HAPSTR1 in PARPi sensitivity.

We respectfully disagree that the suppression of elevated G4 levels and PARP-inhibitor sensitivity in siBRCA2 cells by HAPSTR1 overexpression is inconsistent with these proteins functioning in parallel pathways. The epistasis analysis shown in Figure 4 demonstrates that combined disruption of HAPSTR1 and BRCA2 results in additive increases in G4 nuclear foci, as well as enhanced sensitivity to PDS and PARP inhibition. This pattern supports a model in which HAPSTR1 and BRCA2 operate in parallel, partially redundant pathways to limit G4 accumulation and modulate PARP-inhibitor sensitivity. Within this framework, it is plausible that increased activity of one pathway can compensate for loss of the other, thereby reducing G4 levels and restoring PARP-inhibitor resistance. The experiments presented in Figure 5 specifically test this model. Thus, the finding that HAPSTR1 overexpression reduces G4 foci and PARP-inhibitor sensitivity in BRCA2-depleted cells is fully consistent with these pathways acting in parallel.

With regards to the mechanism of how HAPSTR1 reduces G4 foci in siBRCA2 cells, this would be consistent with it coordinating the removal or destabilization of G4 and R-loop structures to prevent replication fork collapse and genome instability, as raised in the Discussion section.

For all graph data measured from immunofluorescence (Figures 1a, b, d, e, Fig2. a-d, g, Fig. 3, 4, fig. 5a, h) please provide convinving image data, at least the representative staining). This is important since antibody to G4 and R-loop are all with background noises. Overall intensity and measuring specific foci would generate quite big differences. Overall, the data in graph do not seem convincing.

We now include representative images for all figures highlighted in the Supplementary Data file. With respect to the comment that the data are not convincing, we note that all differences on which our conclusions are based are statistically significant, thereby supporting the validity of our interpretations.

The G4 foci per nucleus do not seem convincing, as the untreated U2OS cells already have many (Fig. 1C), and that G4 in replication and for transcription are not differentiated throughout the scoring.

It is entirely expected that untreated U2OS cells display detectable G4 foci. As the reviewer notes, G4 structures play important functional roles in gene regulation, and their presence in unperturbed cells is well established. As outlined in the introduction to the manuscript, G4-forming sequences have been mapped extensively through computational prediction, as well as experimental approaches such as ChIP and CUT&RUN. Therefore, the G4 foci we observe in untreated U2OS cells is not unexpected. Whilst different staining protocols and microscope settings will inevitably result in different thresholds of detection, our data are consistent with previous reports of G4 nuclear staining (e.g. PMID: 30591567; PMID: 23422559).

The survival data u Fig 4b seem to suggest that U2OS cells are quite resistant to siBRCA2 and/orsiBRCA2+HAPSTR1 depletion. Increasing concentration of PDS do not significantly affect the toxicity. Please explain.

It is important to note that in the original Figure 4b (now Figure 4a) the untreated cells serve as the reference and therefore show 100% survival. We do not fully understand the reviewer's comment that increasing concentrations of PDS do not significantly affect toxicity. In our experience, cells are generally quite resistant to PDS. Importantly, however, although the sensitization observed in *HAPSTR1* KO or BRCA2-depleted cells is subtle, our statistical analysis clearly indicates that these differences are significant.

Nevertheless, we acknowledge this point and to address this comment we have revised the text to say: 'Consistent with a role for BRCA2 in G4 homeostasis, BRCA2 depletion led to a subtle but significant sensitisation of cells to the G4-stabilizing ligand PDS to a similar extent as HAPSTR1 loss' (page 10).

Fig. 5a, What is the mechanism underlying the overcome of G4 foci per nucleus by HAPSTR1 overexpression, which was increased by depleting BRCA2?

Please see our response to this reviewer's comment above expressing the concerns that suppression of the BRCA2-dependent increase in G4 levels by HAPSTR1 overexpression is incompatible with these factors acting in parallel pathways.

Additionally, we would also like to reiterate that the central objective of this study is to elucidate the basis by which specific DNA lesions drive PARP-inhibitor toxicity in defined genetic contexts, rather than to establish the precise biochemical activities through which HAPSTR1 may regulate G4 structures. Nonetheless, we acknowledge the importance of this point and have expanded the Discussion to outline potential mechanisms by which HAPSTR1 could influence G4 biology.

The level of expression *by transfection and depletion of HAPSTR1 should be provided by WB.*

See response above.

Figure 1b and 1c: The authors claim that G4 accumulation causes R-loop accumulation. However, in untreated HAPSTR1-deficient cells, G4 levels are elevated, whereas R-loop levels are not increased (Figure 1a). This discrepancy challenges the model that G4s drive R-loop formation under basal conditions. Please provide a clearer mechanistic explanation or hypothesis for why R-loops are not elevated despite G4 accumulation.

See response to reviewer 1, minor point 2.

The description of HAPSTR1Δ.3 of Figure 2a is required in the manuscript.

We now clearly state that *HAPSTR1*Δ.2 and *HAPSTR1*Δ.3 are two independently derived HAPSTR1 knockout cell lines and have referenced the original study in which they were generated (page 6).

The authors need to clarify the γH2AX intensity in HAPSTR1Δ.2 since HAPSTR1 depletion increased γH2AX in previous work (Sharma et al., Nat Commun, 2023). whereas there is no significant difference in Figure 1d and Figure 2c. In addition, the immunostaining of gamma-H2Ax should be analyzed by number of foci and the intensities, not the overall nuclear intensity.

Please note that our original study assessed γH2AX induction in response to hydroxyurea exposure, whereas in the present study we evaluate γH2AX both in untreated cells (distinguishing replicating from non-replicating cells) and following PDS treatment. The two

datasets are therefore not directly comparable. Nonetheless, we appreciate the reviewer's concerns and recognize that the inclusion of related experiments in Figures 1d and 2c of the original manuscript may have caused confusion. To address this, we now present only the γH2AX analysis in a revised Figure 1d. This figure contains a new dataset which, in line with comments from Reviewer 1, includes assessment of the effect of the transcription inhibitor DRB on DNA damage. Following this reviewer's suggestion, γH2AX is now quantified as foci per nucleus rather than by signal intensity. These revised data clearly demonstrate a significant increase in γH2AX in untreated replicating U2OS cells, which is further elevated in *HAPSTR1* KO cells.

Figure 2c seems to reconstitute the same results of Figure 1d (untreated).

See above and response to reviewer 1, minor point 6.

The representative PLA image in Figure 2e (U2OS cells) does not reflect the quantified data (~30 foci) shown in the accompanying graph. The image should be replaced or revised to better represent the quantification.

Please note that although the average number of PARP1–G4 PLA foci in U2OS cells represented in Figure 2e is approximately 30, the distribution is broader, ranging from ~10 to ~70 foci per cell. As such, selecting images that precisely reflect the mean number of foci is not representative of the overall dataset. Instead, we believe that the quantitative data presented in the original manuscript more accurately convey the full distribution of PARP1–G4 PLA foci observed in all conditions. For this reason, we are reluctant to replace the existing representative images.

Figure 1d: The authors state that DNA damage (γH2AX) is only elevated in S-phase cells (EdU+). However, in the graph, the EdU-negative cells in HAPSTR1-depleted conditions appear to show a modest increase in γH2AX after PDS treatment. Given this, I suggest increasing the number of biological replicates or providing statistical power analysis to support the claim that the difference is non-significant (ns).

As suggested, we have revised Figure 1d to include additional biological replicates to enhance statistical power and have quantified γH2AX foci rather than overall signal intensity. These updated data show that, in the absence of PDS, a significant increase in γH2AX is observed only in replicating cells. In contrast, PDS treatment leads to elevated γH2AX levels across all cell cycle phases. We have revised the text to more clearly describe these findings.

Figure 2 (especially 2d and 2f): The authors propose that PARP1/2 and HAPSTR1 function in compensatory pathways to resolve G4s. The proximity ligation assay (PLA) in Figure 2f supports this, as PARP inhibition reduces HAPSTR1-G4 proximity (likely due to PARP1 trapping), while PARP1/2 KO restores it. However, in Figure 2d, G4 accumulation in HAPSTR1 KO cells treated with olaparib appears additive rather than compensatory, raising questions about the extent of functional redundancy. To solidify the claim of compensation, I strongly recommend including functional rescue experiments, such as G4 reporter assays or replication fork progression analysis, to determine whether one pathway (HAPSTR1 or PARP1/2) can substitute for the loss of the other in suppressing G4-related replication stress.

We thank this reviewer for raising this point. We believe this reflects the presence of two G4 populations (See response to Reviewer 1, minor point 8). We now also include extensive replication fork analyses using DNA fibre assays to assess ssDNA gaps (Figure 3b, c, e, 4e) and replication fork stability (Figure 4g), which further support our model that HAPSTR1 and PARP1 function in parallel pathways to suppress nuclear G4 levels.

Typographical errors in Figure 1a "HAPSTR11∆.2" and Figure 1c "HASPTR1" should be corrected to "HAPSTR1∆.2" and "HAPSTR1" each.

Noted and corrected.

REVIEWER 6

We thank this reviewer for their comments and for considering our manuscript a strong candidate for publication. We now provide clarification and additional experiments to address their concerns.

1. *The authors state at the bottom of page 4 that their data "demonstrate that the elevated G4 structures observed in HAPSTR1 KO cells are … a cause of R-loop formation". However, just a few lines above that, they state that "although G4 levels were significantly increased in untreated HAPSTR1 KO cells, R-loop levels were independent of HAPSTR1 status (figure 1c)." These statements appear to be conflicting. If G4 structures cause R-loops, would one not expect increased R-loops in HAPSTR1 knockout cells since these cells have more G4 structures? This is an important issue to be resolved, since the authors go on to re-state this claim on page 5: "..our findings that R-loop accumulation in HAPSTR1 KO cells is a consequence of G4 deregulation (figure 1a-c)".*

We recognise that the original wording may have suggested G4s and R-loops are universally linked, which was misleading. Our data argue against this. In *HAPSTR1* KO cells, increased G4s (Supplementary Figure S2a, b) and associated γH2AX induction (Figure 1d) occur independently of transcription, indicating that R-loops are not required for G4 formation, or the resulting DNA damage. Instead, we observe that R-loops arise only following G4 stabilisation with PDS in these cells (Figure 1a). Thus, elevated G4s in HAPSTR1-deficient cells appear transcription-independent, whilst R-loops only occur in response to PDS-induced G4 stabilisation. We therefore conclude that elevated levels of G4 structures observed in *HAPSTR1* KO cells are independent of transcription and are a cause of R-loop formation. We have revised the text (page 5-6) to clarify this point.

………in figure 2b the authors report a different result than in figure 1c: In the untreated (-EdU) samples there is no difference between G4 foci in control vs HAPSTR1 KO cells. In contrast, figure 1c, which appears to be an identical experiment, shows a statistically significant increase, which is described by the authors as mentioned above. Which result is true? How many times were these experiments performed independently, and is the increase reproducible or not?

While the experiments shown in Figures 1c and 2b are similar in design, they address distinct aspects of G4 regulation. In Figure 2b, we assessed nuclear G4 levels in U2OS and *HAPSTR1* KO cells under basal conditions, without PDS treatment. The -/+ symbols in this figure denote whether cells were pulsed with EdU to identify those undergoing DNA replication. The reviewer is right to point out that there is no difference between G4 foci in control vs *HAPSTR1* KO cells. However, it is important to note that 'control' cells in this instance are non-replicating Edu- cells. EdU+ (replicating) cells exhibit elevated G4s.

In Figure 1c, we do not assess cells depending on EdU status. Instead, the –/+ symbols indicate the absence or presence of PDS treatment. The –PDS condition corresponds to untreated cells similar to those shown in Figure 2b. However, unlike Figure 2b, these cells were not separated by cell cycle phase and therefore include both replicating and non-replicating populations. The significant increase in G4s observed in *HAPSTR1* KO cells in Figure 1c is thus consistent with the elevated G4 levels detected in replicating (EdU+) cells in Figure 2b.

2. *If the G4 structures are indeed the cause of increased R-loops in HAPSTR1 KO cells, it would be important to investigate if the increased DNA damage observed in these cells (gH2AX foci, micronuclei) is caused by increased R-loops, or represent a more direct impact of G4 structures, without going through an R-loop intermediate. To address this, the authors could test if the DNA damage in PDS-treated HAPSTR1 knockout cells is suppressed by the transcription inhibitor (DRB) which blocks R-loop formation.*

We include these experiments in the revised manuscript - please see response to Reviewer 1, comments 3.

3. *The micrographs in figure 1f are not convincing. The micronuclei shown are at the edge of the image and partially cut out. Wider field images need to be shown, to be able to judge the data.*

We have now included wider field images in the figure.

4. *Since the PARP1-G4 PLA experiment in figure 2e appears to be the first demonstration that PARP1 is recruited to G4 structures in cells, additional controls should be performed to confirm the specificity of the assay. For example, does treatment with the G4 stabilizer PDS increase the PARP1-G4 PLA foci?*

We have performed these experiments (Supplementary Figure S5c) and now describe them in the revised manuscript (page 8).

5. …. *It is only through an S1 nuclease assay that ssDNA gap accumulation can be unequivocally shown. Without S1 nuclease treatment, regardless of labeling schemes, one cannot conclude that ssDNA gaps are measured. While gaps in HAPSTR1 KO cells are convincingly shown using S1 nuclease treatment in figure 3b, subsequent figure panels (3c, 3d, 3f, 4d) claim to be investigating ssDNA gaps without employing S1 nuclease assays. All these experiments need to be performed using S1 treatment…..…..Moreover, it is customary to show the results of S1 nuclease fiber assays as IdU/CldU ratios, to normalize for differences in fork speed.*

We thank the reviewer for this important comment. We have now replaced all ssDNA measurements with the appropriate assays employing S1 nuclease. The only exception is the original Figure 3f, which assessed ssDNA formation in response to PDS. This has instead been replaced with an experiment suggested by Reviewer 1 demonstrating that the PDS sensitivity of *HAPSTR1* KO cells is suppressed by PrimPol. We felt this experiment was more appropriate, as it complements our data showing that ssDNA gap formation in *HAPSTR1* KO cells is dependent on PrimPol and more directly addresses the link between G4 stabilization by PDS and PrimPol-dependent gap formation.

The revised data in Figure 4e illustrate that olaparib-induced ssDNA gap formation in the absence of BRCA2 and HAPSTR1 is epistatic, suggesting that these factors function in the same pathway to suppress ssDNA gaps. This finding was unexpected, given that combined disruption of HAPSTR1 and BRCA2 resulted in increased sensitivity to PARP inhibitors (Figure 4d), underscoring the importance of the reviewer's comment regarding the use of appropriate assays to measure ssDNA gaps. Accordingly, we instead assessed replication fork stability in these cells and identified that in the absence of BRCA2 and HAPSTR1 olaparib induced severe replication fork instability, relative to siBRCA2 or HAPSTR KO alone. Together, we therefore conclude that HAPSTR1, BRCA2, and PARP1/PARP2 operate through distinct pathways to regulate G4 levels, and that the toxicity of PARP inhibitors in the absence of both BRCA2 and HAPSTR1 is driven by replication fork instability rather than

ssDNA gap formation. The Results and Discussion have been revised accordingly to incorporate these new findings.

With regard to the comment on plotting DNA fibre length versus IdU/CldU ratios, we presented fibre length measurements because this representation is most commonly used for this type of analysis (see PMID: 36455556; 34216544; 34624216; 34508659; 38753485). However, in response to the reviewer's suggestion, we have additionally analysed the data using IdU/CldU ratios (Supplementary Figure S6b–e) and obtained consistent results.

6. *Figure 3g is misleadingly presented as ssDNA-G4 PLA. In fact, the authors treat cells with BrdU and perform a BrdU-G4 PLA. This needs to be clearly acknowledged, as other interpretations of these results can be possible. Next, the authors need to present proper single antibody controls for this experiment, as they showed for other PLA experiments (eg in figures 2e, 2g). Finally, to gain confidence that this assay indeed detects ssDNA gaps at G4 structures, additional controls are needed. For example, depletion of PrimPol can be performed to suppress ssDNA gap formation.*

We have revised this figure in several ways (page 9; Figure 3g). As suggested, we have relabelled the PLA *as* BrdU–G4 PLA to more accurately describe the data. In addition, we have included single-antibody controls for this experiment.

As the reviewer correctly points out, BrdU staining reflects the presence of ssDNA rather than specifically identifying ssDNA gaps. Nevertheless, consistent with the reviewer's suggestion, we observed a reduction in the BrdU–G4 PLA signal following PrimPol depletion in *HAPSTR1* KO cells. This supports our interpretation that the detected signal likely represents PrimPol-dependent ssDNA gaps in proximity to G4 structures as opposed to ssDNA formed through other events such as replication fork collapse/resection.

7. *The G4 accumulation in BRCA2 and HAPSTR1 knockout cells seems additive rather than synergistic, arguing against a synthetic lethality phenotype. It would be informative to measure DNA damage (gH2AX foci) in these cells, with and without olaparib treatment.*

Given this section of the manuscript centres on replication stress, we believe that analyses focused on replication fork dynamics are more informative than measurements of DNA damage. Accordingly, we have added substantial new data examining ssDNA gap formation and replication fork stability in cells lacking HAPSTR1 or BRCA2, both in the presence and absence of PARP inhibitor treatment (Figure 4e–g). These experiments clearly demonstrate increased replication-fork degradation, which we believe provides a more informative readout of the underlying situation than γH2AX alone. We therefore hope that these additional data adequately address the reviewer's concerns.

8. *Is the phenotype restricted to BRCA2, or is BRCA1 involved in a similar manner? Does BRCA1 depletion increase G4 and olaparib sensitivity of HAPSTR1 KO cells? Does HAPSTR1 overexpression rescue the olaparib sensitivity of BRCA1-deficient cells?*

See response to Reviewer 1, comment 2.

9. *The result that HUWE1 depletion rescues the olaparib sensitivity of BRCA2-deficient cells was previously published (PMID: 33257658). This should be acknowledged.*

We thank this reviewer for highlighting this report and we have acknowledged this in the discussion (page 17).

10. *I found the description of the data presentation in the figure legends somewhat confusing. For PLA and fiber experiments in Figure 3 (and other figures) the authors state that "Data represent the SEM from 3 independent experiments". I presume they mean that "" as they indicate for other figures (eg figure 2g). Even so, this is confusing because the data they show is not actually the means of the three experiments, but rather, if I understand correctly, they combine all individual measurements from the three experiments in one large dataset and they then calculate the mean and SEM for the whole dataset. This should be made clear. Moreover, if indeed three independent replicates were performed for these experiments, it would be informative to plot them separately (or to plot the mean values of each individual experiment in a separate graph) in order to be able to assess the reproducibility of the result. This would be particularly important for results with small differences which may or may not be reproducible (see point 1 above).*

We have amended Figure 3 to state that data sets represent mean values ± SEM of 3 biological independent experiments. We have also clarified in the figure legend that this analysis was performed on combined data sets from 3 independent experiments.

With regard to the request to plot independent repeats on separate graphs, doing so for all fibre assays (both fibre length and IdU/CldU ratios), as well as the PLA data in Figure 3, would require 13 additional figures resulting in at least 39 extra panels. As inter-experimental variability is already accounted for in the statistical analyses, and the underlying data are provided in the source files, we believe including these graphs in the main manuscript would lead to unnecessary expansion and detract from the clarity and focus of the primary figures. We therefore respectfully request that this analysis remain in the source data rather than being incorporated into the main figures.

RESPONSE TO REVIEWERS

We thank the reviewers for their assessment of our revised manuscript. Below, we provide a point-by-point response to their additional comments. Changes made during the first round of revision remain indicated in red, while further revisions introduced in this round are highlighted in green.

REVIEWER 1

Major point:
I asked the authors to show whether the PDS sensitivity (and the observed DNA damage) in HAPSTR1 deficient cells could also result from the accumulation of R-loops rather than G4's. The authors explained that technical limitations prevented them from using RNAseH expression to investigate this which we understand. Instead, they performed short DRB treatment to show independence of transcription. However, this data cannot rule out the possibility that R-loops are the determinant of the toxicity/damage. First, it is not shown that the short DRB treatment reduces R-loops. It needs to be demonstrated that R-loops are no longer elevated in PDS treated HAPSTR1 KO cells upon DRB treatment. This is a crucial control and especially important since it has been shown that R-loops can also form independently of ongoing transcription. This could be done by repeating the experiment in Figure 1a in the presence of DRB, and/or by performing G4-R-loop PLA assays in HAPSTR1 KO cells treated with PDS without or without DRB.

We now include data demonstrating that DRB treatment suppresses PDS-induced R-loop accumulation in both U2OS and HAPSTR1 KO cells (Supplementary Figure S1e), and we have referenced these findings in the main text (page 5).

Points to be addressed textually:
1) *While the addition of the 53BP1 focus data is informative and important and follow a similar trend to gammaH2AX foci, EdU-negative HAPSTR1 KO cells exhibit enhanced 53BP1 foci (without accumulation of G4s, Figure 2b). This is surprising and should be explained.*

The increased 53BP1 foci observed in EdU-negative HAPSTR1 KO cells likely represent large 53BP1 nuclear bodies, which are well-established markers of unrepaired DNA lesions carried over from the preceding S-phase. This interpretation is consistent with our previous findings (PMID: 37591890), where we reported the accumulation of 53BP1 bodies in untreated HAPSTR1-deficient cells in G1. We have noted this possibility in the revised manuscript (page 6).

2) *The direct link between genome instability and G4 structures has not been demonstrated. For this, the authors should map DNA damage to G4 sites in the genome, however, this has not been done. Phrases like in the summary: 'genome instability at G4 sites' should be rephrased.*

We agree that a direct genome-wide mapping of DNA damage to G4 sites has not been performed in this study and have modified the summary accordingly.

3) *With respect to testing other DNA damaging agents in HAPSTR1 KO cells, it is good to know that this has been performed in previous work of the authors. It would help to briefly describe this in the introduction. However, the statement that these cells are proficient in HR cannot be concluded from the observation of intact HU-induced RAD51 foci because these are a marker of replication fork protection rather than for HR. If this is the evidence, this statement needs to be rephased.*

We have now included a brief description in the Introduction (page 4) to describe our previous observations that HAPSTR1 KO cells are not sensitive to DSBs, or DNA base damage. Additionally, we have revised the relevant statement in the Discussion (page 13) to remove the inference that RAD51 foci reflect HR proficiency.

4) *The authors should mention in the method section that they are aware of the non-specific staining of the S9.6 antibody and refer to the manuscript in which they performed the controls for this.*

We have inserted a brief description of our validation of the S9.6 antibody in the methods, citing the relevant paper (page 19).

5) *The manuscript became rather lengthy after revision and lacks clarity here and there, especially in the discussion section. In addition, there are several sentences in the discussion that need rephrasing*:

P13
1. *"we report that stabilising G4s contributes to this phenotype to suppress replication-associated DNA damage and genome instability."*

Unclear, stabilising G4s does not suppress replication-associated DNA damage.

Re-phrased as requested.

2. *"However, our observations that HAPSTR1 KO cells are not sensitive to DSB-inducing agents and are HR proficient would argue against this."*

See Point 3 above.

See response to point 3 above.

3. "*Overall, these data support a model in which G4s act as replication barriers that induce DNA damage signalling to γH2AX, with R-loops contributing secondary damage when present."*

The link between G4s, replication stress, and DNA damage has been extensively characterised by the previous studies. Could the authors provide references? Whether R-loops contribute to secondary damage when present is not fully supported in this manuscript. This could simply result from stabilised G4s that cannot be unwound by DNA helicases. Please rephrase.

Unfortunately, the limit of 70 references for this journal constrained our ability for extensive citations. Within this framework, we did our best to cite the relevant literature highlighting the link between G4s, replication stress and DNA damage in the Introduction of the original manuscript. We have now included these references in the Discussion, as requested.

With respect to the contribution of R-loops to secondary DNA damage, our data show that a subset of DNA damage sites marked by 53BP1 foci in PDS-treated cells is transcription dependent. This observation is not readily explained by unresolved G4s alone and instead points to the involvement of a transcription-associated structure, most likely R-loops. We therefore consider it appropriate to retain the conclusion that a transcription-dependent process contributes to secondary DNA damage. However, to avoid overinterpretation, we have revised the text to state that our data "support a transcription-dependent structure such as R-loops contributing to secondary damage, particularly in response to PDS."

P.14
4. *“Whether HAPSTR1 prevents G4 formation, directly remodels these structures, or recruits additional processing factors for their resolution remains unclear.”*

These possibilities are not mutually exclusive. “How HAPSTR1 restrains G4 formation remains unclear” would be better.

Modified as suggested.

P.15
5. *“We observe that ssDNA gaps in HAPSTR1 KO cells exposed to PARP inhibitors are induced by PRIMPOL-dependent repriming of replication forks that require TLS for repair”*

This phenotype is not rescued, and therefore this sentence should be rephrased.

Apologies – we missed this statement following this reviewer’s comment during the first round of revision and is now corrected (page 16).

P.16.
6. *“Notably, loss of BRCA2 results in elevated G4s at both telomeric and non-telomeric regions (Supplementary Figure S7c), suggesting a genome-wide increase in G4s that cause replication obstacles and DNA damage.”*

This sentence suggests a role of BRCA2 before replication forks encounter G4s, which is unclear from the data in this manuscript. This sentence should be rephrased or substantiated by data and/or previous studies.

We have rephrased this sentence as requested.

7. *“Notably, ssDNA gaps induced by HAPSTR1 loss localize to G4 structures (Figure 3g).”*

Figure 3g does not suggest that most ssDNA gaps are formed at G4 structures. ssDNA could be formed in the absence of G4s. Needs rephrasing.

This sentence has been amended to ‘Notably, at least a subset of ssDNA gaps induced by HAPSTR1 loss localize to G4 structures’ (page 17).

Related to this point, whether elevated G4 levels in BRCA2 KD/HAPSTR1 KO cells is causal or consequence of ssDNA accumulation is unclear. This should be addressed textually.

We have inserted a sentence to address this caveat in the discussion (page 16).

8. “*Our data point to increased replication fork instability, …..”*

Change to : “G4-mediated replication fork instability, ….”

Changed as requested (page 17).

REVIEWER 3

1. *Possible contribution of R-loops upon PDS treatment*

Treatment with the G4 stabilizer PDS is central to this study. Because PDS treatment can also induce R-loop accumulation, particularly at replicating telomeres, it is important to clarify

whether R-loops contribute to the observed phenotypes.

This could be assessed by transient expression of RNase H1. The authors state that PDS treatment cannot be prolonged for 72 hours as in the case of RNase H1 overexpression. Nevertheless, optimization of PDS treatment together with transient RNase H1 expression should at least be attempted. This would allow estimation of how much of the S9.6 signal represents bona fide R-loops and would help determine whether the consequences of G4 elevation are indeed independent of R-loops, as suggested.

We have considered the use of RNase H1 expression to directly assess the contribution of R-loops. However, as outlined in our initial response, this approach is challenging when applied across multiple genetic backgrounds and with longer treatment windows. While transient transfection is feasible in principle, we were concerned about variable transfection efficiencies between cell lines, particularly given the need to combine RNase H1 expression with siRNA-mediated depletion in some experiments. We also attempted to employ inducible RNase H1-expressing cell lines to overcome these limitations. However, we encountered significant toxicity associated with these cells which prevented the use of this system.

Given these constraints, as an alternative approach we employed transcriptional inhibition using DRB, as suggested by Reviewers in the first round of revision. This strategy enabled us to assess the contribution of transcription-dependent structures under experimentally tractable conditions. These data indicate that elevated G4s and replication-associated DNA damage (as measured by γH2AX) in HAPSTR1 KO cells occurs independently of transcription. In response to Reviewer 1's comment, we have now demonstrated that short-term DRB treatment effectively suppresses PDS-induced R-loop accumulation, further validating this approach. Taken together, and given the consistent use of DRB across our experiments, we believe these data provide strong evidence that R-loops are not the primary driver of the observed phenotypes.

In Supplementary Fig. S1d, the authors report an increase in S9.6 mean nuclear intensity. It is unclear how this value was quantified. Does it represent overall nuclear intensity? Because EdU labeling was included in this experiment to distinguish replication-associated signals, the quantification method should be clearly described. If overall nuclear intensity was measured, this metric may not accurately reflect discrete R-loop foci. The Methods and figure legends currently lack sufficient information.

We have expanded the figure legend of Figure S1d to include this information.

2. *Level of HAPSTR1 expression*

The level of FLAG-HAPSTR1 expression across the experimental conditions should be more clearly documented. Although overexpression is shown in Fig. S8 and by RT-PCR, the data appear to indicate expression levels exceeding threefold, whereas the text suggests approximately twofold.

Given that western blots are inherently semi-quantitative, we instead used qPCR to quantify HAPSTR1 expression levels and have provided a detailed description of the qPCR protocol in the Methods. It is unclear how the reviewer arrived at the statement that '*data appear to indicate expression levels exceeding threefold'* and if this was stated anywhere in the manuscript we apologise for the confusion. For clarity, in FLAG-HA-HAPSTR1 expressing U2OS cells the values for overall HAPSTR1 expression (endogenous HAPSTR1 + FLAG-HA-HAPSTR1) are increased 2.567-, 2.085-, and 1.404-fold relative to U2OS cells. This

corresponds to an average increase of 2.02-fold. We now report this value in the revised manuscript (page 12) and have expanded the legend of Figure S8b to provide greater clarity.

Because the study consistently compares U2OS, HAPSTR1Δ2, and HAPSTR1Δ2 + HAPSTR1 cells, western blot analysis including all three cell types should be provided in the supplementary information. In Fig. S8, the comparison appears limited to U2OS versus overexpression cells, without inclusion of the HAPSTR1Δ2 line.

The data presented in Figure S8 show HAPSTR1 expression levels in U2OS cells with or without ectopic expression of recombinant FLAG–HA–HAPSTR1. These experiments were included as the cells have not been previously published. The data are to support analyses in the final results section that examines the effect of HAPSTR1 overexpression on G4 levels and PARP inhibitor sensitivity in BRCA2-depleted U2OS cells, not the HAPSTR1 KO cells.

Importantly, these experiments are distinct from those performed in HAPSTR1 KO cells complemented by re-expression of FLAG–HA–HAPSTR1 in a *HAPSTR1*-null background. These complemented cell lines have been previously characterised (PMID: 37591890), including validation of FLAG–HA–HAPSTR1 expression in these genetic contexts.

3. *Micronuclei analysis and antibody specificity*

Antibodies detecting G4 structures and R-loops are known to exhibit background signals. In Fig. 1f, the authors show that the percentage of G4-positive micronuclei increases following PDS treatment. It would be informative to first present the overall level of micronuclei induction and then quantify the fraction of micronuclei that contain G4 signals.

In particular, the extent to which PDS treatment itself induces micronuclei, and whether this induction differs depending on the presence or absence of HAPSTR1, would provide a clearer interpretation of the data.

The requested analyses of micronuclei induction upon PDS treatment were included in the original manuscript. To clarify, Figure 1e shows the frequency of micronuclei induced upon PDS treatment (expressed as the percentage of cells containing micronuclei) in U2OS cells and *HAPSTR1* KO cells, with or without complementation by FLAG–HA–HAPSTR1 expression. Figure 1f shows the proportion of these micronuclei that are positive for G4 structures.

4. *Telomeric versus non-telomeric G4 and R-loops*

In Supplementary Figs. S4–S5 and Fig. S7, G4 immunostaining clearly increases throughout the nucleus, consistent with the authors' claim that DNA damage is not restricted to telomeres. However, telomeric foci can be defined and quantified (even though U2OS cells may show increased numbers due to aneuploidy). Therefore, the analyses in Fig. S1a and Fig. S7 would benefit from explicitly reporting the frequency of TRF1-positive telomeric G4 foci, with non-telomeric G4 foci analyzed separately.

Such analysis would more precisely define the contribution of telomeric versus non-telomeric events, which is important given the unique vulnerability of telomeric DNA to replication stress and DNA damage.

There seems to have been some confusion regarding the figure numbers and analyses in this comment. The reviewer is requesting clarification of G4 immunostaining at either telomeric or non-telomeric regions. The data concerned with this are not Fig. S1a, as stated by the reviewer, but Figures S5a and S7c. We are pleased that the reviewer agrees with our interpretation that '*G4 immunostaining clearly increases throughout the nucleus, consistent*

with the authors' claim that DNA damage is not restricted to telomeres'. However, we were confused by their subsequent request to report '*the frequency of TRF1-positive telomeric G4 foci, with non-telomeric G4 foci analyzed separately'.* These are precisely the analysis that are presented in Figures S5a and S7c – the data represent the average G4 foci per nucleus that co-localise with TRF1 (G4s at telomeres) or do not localise with TRF1 (non-telomeric G4 foci).

In the revised manuscript, the authors include RPE cells and argue that the role of HAPSTR1 in G4 homeostasis is independent of ALT status. I agree that the effect is not restricted to telomeres. However, the previous concern was not simply whether telomeres differ from other genomic regions, but whether HAPSTR1 might differentially influence replication fork stalling caused by G4 stabilization versus G4-related transcriptional processes.

The authors state in Supplementary Fig. S5 and in the text that G4 elevation in HAPSTR1 knockout cells treated with PARP inhibitors is not restricted to telomeres and is independent of ALT status. In my view, the data presented do not fully support the latter conclusion. Given the genome-wide effects of the G4 ligand demonstrated in the revised manuscript, it would be sufficient to state that the effects are not restricted to telomeres. The current data do not conclusively establish that the phenomenon is independent of ALT status.

With respect, we disagree with this comment and maintain that our data support the conclusion that elevated G4 levels in HAPSTR1-deficient cells are not dependent on ALT status. Specifically, we observe increased G4 accumulation in both ALT-positive U2OS cells (Figures 2d and S5a) and ALT-negative RPE1 cells (Figure S5b). These findings clearly indicate that the phenotype is present irrespective of ALT status. As such, we feel our conclusion that G4 elevation is independent of ALT status in valid.

RESPONSE TO REVIEWER 3

We thank this reviewer for their continued engagement with our manuscript.

With regards to the reviewer's comment that 'the revised manuscript still does not provide direct evidence demonstrating the specificity of the S9.6 signal (e.g. reduction upon RNase H1 expression), nor does it convincingly establish the extent to which R-loops contribute to the observed phenotypes', as described in our previous revision, we established robust conditions for S9.6 staining in a previous study (PMID: 37591890). In the current study, we have also demonstrated that our phenotypes are maintained under conditions where R-loops are suppressed using the transcription inhibitor DRB. Nevertheless, we acknowledge the lack of additional RNaseH1 controls and now include caveats regarding the limitations of our approach arising from the absence of this experiment (page 13).

We also thank the reviewer for highlighting the possibility that differences in HAPSTR1 expression levels between cell lines could influence the phenotypes observed under different conditions. However, with the exception of the experiments presented in Figure 5, all analysis concerned with HAPSTR1 are performed in HAPSTR1 KO cells with their matched parental controls. Furthermore, although both U2OS and RPE1 cell lines were used in this study, we do not directly compare the magnitude of phenotypes between KO cell lines; rather, we assess whether the same phenotype is observed in each background. Thus, the phenotypes under investigation are measured in cells lacking HAPSTR1 expression, and therefore we do not consider variation in endogenous HAPSTR1 levels between cell lines to be a significant factor influencing these observations. Nevertheless, we agree that HAPSTR1 expression levels may affect cellular responses under certain conditions. We have therefore added a statement to the Discussion (pages 17–18) acknowledging this possibility, while noting that it is not directly relevant to the majority of experiments in this study, which focus on phenotypes in HAPSTR1-deficient cell lines.

Finally, in response to the reviewer's comments regarding the analysis of telomeric versus non-telomeric G4 signals, we believe the key observation is that under our experimental conditions we detect elevated levels of G4 structures that both co-localise and do not co-localise with TRF1. We therefore consider our conclusion that the observed effects are not restricted exclusively to either telomeric or non-telomeric G4s to be supported by the data. However, we acknowledge that telomeric sequences are more densely enriched in G4-forming motifs than non-telomeric regions and may therefore be preferentially detected in our immunofluorescence experiments. We have now included this caveat in the manuscript (page 8).

Comments on "G-quadruplex homeostasis is a key determinant of PARP inhibitor toxicity in BRCA2-deficient cells"

In this manuscript, the authors report that depletion of HAPSTR1 sensitizes cells to PARP inhibitors, focusing on the deregulation of G-quadruplex (G4) structures as a key determinant of PARPi toxicity, particularly in BRCA2-deficient cells. The authors posit that HAPSTR1, a substrate of the E3 ligase HUWE1 involved in pleiotropic stress responses, acts as a suppressor of G4 accumulation during DNA replication. They further argue that loss of HAPSTR1 in U2OS ALT cells results in increased replication stress, single-stranded DNA gaps, and heightened sensitivity to PARPi. Additionally, the authors propose that HAPSTR1 and BRCA2 function in parallel, independently, yet synergistically, influencing cellular responses to PARPi. They also suggest that HUWE1-dependent degradation of HAPSTR1 modulates G4 homeostasis and contributes to PARPi resistance in BRCA2-deficient contexts.

While the work has the potential impact in clinical application, the manuscript currently appears mostly descriptive and preliminary in its current form. It lacks specific mechanism, likely due to pleiotropic effects following HAPSTR1 depletion, which are not sufficiently dissected. Several interpretations in the manuscript are overextended or inconsistent with previous literature. Multiple facets discussed require clarification and additional mechanistic validation before this work can be considered. Below are the major and minor concerns.

Major Comments:

1. The primary concern is the overly descriptive nature of the study, which touches upon many aspects of DNA damage, replication stress, and synthetic lethality without extending beyond existing knowledge. The repeated use of terms such as "deregulation of G4," "suppression of G4," or "tolerance of G4" lacks clarification, particularly regarding the basis of G4 regulation.
2. The differentiation between types of G4 structures-telomeric versus promoter G4s is crucial. It has been shown that telomeric G4s, which form during lagging strand synthesis and interconvert to remodel stalled forks, are dynamically regulated, with BRCA2 playing a key role in their control (Lee et al., Nature Commun. 2023, PMID: 38587189). The authors, however, refer to "G4 deregulation" without defining what constitutes "regulation" in this context. Clarification is essential for interpreting their findings.
3. The experimental system appears to investigate G4 formation in an asynchronous population of U2OS ALT cells, using the G4 stabilizer PDS without synchronization in S phase. Given that G4s serve physiological roles in transcription regulation and telomere maintenance-functions that are cell cycle-dependent-the authors should differentiate between replication-associated G4 structures and those involved in transcription. Treating G4s as merely "bad" structures is an oversimplification. The manuscript would benefit from a

discussion on the physiological versus pathological roles of G4, including appropriate citations and mechanistic insights.

4. The role of HAPSTR1 in G4 homeostasis remains unclear. Although associations between HAPSTR1 and G4 structures are shown via antibody and PLA assays, the mechanistic basis-how HAPSTR1 directly influences G4 formation, stability, or resolution-is not addressed and should be elaborated.
5. The current title suggests a mechanistic focus on G4 homeostasis; however, the manuscript instead offers a broad overview of HAPSTR1 functions with only superficial discussion of G4 regulation. To justify the title, a clear mechanistic link must be established. At present, the data are insufficient to support a direct role for HAPSTR1 in G4 homeostasis.
6. Prior literature demonstrates that BRCA2 interacts directly with telomeric G4 structures to maintain replication (Lee et al., Nat Commun. 2022), and that BRCA2 deficiency prompts G4 accumulation and R-loop formation via disrupted G4 dynamics (Lee et al., Nucleic Acids Res. 2024). Further, Autjors have published previously that HAPSTR1 has been implicated in R-loop resolution via pathways involving BRCA1/Senataxin (Sharma et al., Nat Commun. 2023), which appear distinct from G4 regulation. Given these findings, it is unclear whether G4 accumulation in HAPSTR1-deficient cells is a direct consequence of G4 deregulation or an indirect effect secondary to unresolved R-loops or other secondary effects. Additional evidence is needed to elucidate the mechanistic interplay between HAPSTR1, G4 structures, and R-loops, and how these influence PARP inhibitor responses in BRCA2-null contexts.

Specific Comments:

- The authors demonstrate that transfection of FLAG-HA-HAPSTR1 can rescue the effects of HASPTR1 depletion, particularly regarding G4 deregulation in U2OS cells. However, the expression levels of the transfected FLAG-HAPSTR1—both the overexpression relative to endogenous levels and the depletion efficiency—are not shown in any figures or supplementary data. It is essential to include Western blot data or quantitative assays to confirm whether the exogenous HAPSTR1 is expressed at physiological levels or overexpressed, and whether it effectively compensates for endogenous depletion.
- The cell line used, U2OS, is an ALT (Alternative Lengthening of Telomeres) cancer model where telomeric G4s are known to be deregulated and depend heavily on Break-Induced Replication (BIR), as well as homologous recombination (HR). In particular, when BRCA2 is absent, these cells adapt via BIR mechanisms (references 1–3). Since the study investigates the effects of HAPSTR1 ablation on PARPi toxicity, and suggests involvement of translesion synthesis (TLS), it is crucial to determine whether these effects are cell line–dependent. Including additional cell lines, especially those with different telomere maintenance mechanisms or DNA repair backgrounds, would

strengthen the conclusions and address the potential cell-specific effects of HAPSTR1 depletion.

- Regarding the micronuclei assessment in Figure 1e and related text: micronuclei are byproducts of chromosome mis-segregation, often indicative of genomic instability. The data show a surprisingly high baseline micronuclei frequency in control U2OS cells, even without treatment, which exceeds the typical levels reported in other studies. It would be helpful to clarify how PDS treatment influences micronuclei formation and to provide quantitative data comparing the percentage of micronuclei that are G4-positive versus total micronuclei, to better interpret these results.
- The statement that HAPSTR1 and PARP1/2 act in parallel pathways to facilitate cellular tolerance of G4 accumulation (page 5) warrants reconsideration. Specifically, the term "tolerance of G4" raises questions, as G4 structures are generally considered potentially deleterious or "toxic," especially if stabilized or accumulated beyond physiological levels. The study by Lee et al. (Nature Communications, 2023) highlighted that the dynamic interconversion of G4 structures is fundamental for telomere replication homeostasis. Therefore, the authors should clarify whether their data pertain to perturbations in G4 dynamics (e.g., stabilization, formation rate) or other mechanisms leading to G4 accumulation, and explicitly distinguish between telomere G4s and G4s involved in transcriptional regulation. Proper citations and a nuanced discussion of these mechanisms are necessary.
- The suggestion that loss of HAPSTR1 increases the cellular dependence on PARP1/2-mediated G4 regulation (page 6) appears to overreach. This interpretation should be tempered, with acknowledgment of the current evidence's limitations, and framed more cautiously.
- The references cited for PARP1 binding to G4 structures need to be revised to ensure accuracy and relevancy. Some cited studies may be outdated or not directly applicable to the specific experimental context.
- The investigation of ssDNA gaps at G4 structures, particularly involving HAPSTR1 and PARP1/2, appears to be a complex and distinct topic. In its current form, mixing replication and transcription effects without synchronization, and referencing both processes simultaneously, creates confusion. Since ssDNA gap formation primarily pertains to replication stress, incorporating cell cycle synchronization or stage-specific assays would improve interpretability. Moreover, the suggested involvement of PRIMPOL and TLS, especially in HAPSTR1 knockout cells, is weakly supported. Considering that BRCA2 mainly functions at lagging strand telomere synthesis where G4s form, and not at the leading strand where PRIMPOL operates, it's critical to investigate these discrepancies further.
- As for the claim in page 9 that BRCA2-dependent increases in G4 levels are fully suppressed by HAPSTR1 expression: the mechanistic basis for this suppression remains unclear. Given their proposed placement in parallel pathways regarding

PARPi sensitivity, it appears contradictory that HAPSTR1 overexpression would directly reduce G4 levels derived from BRCA2 deficiency. Clarification and supporting mechanistic data are necessary.

- Concerning the immunofluorescence data (Figures 1a, b, d, e; Figures 2a–d, g; Figures 3–5, and supplemental figures): representative images should be included for validation, especially considering the background noise associated with G4 and R-loop antibodies. Quantification can be significantly impacted by signal-to-noise ratio; therefore, providing example images demonstrating specific staining—preferably with methodologically rigorous controls—is critical for verifying the reported foci.

References

1. Lee, J.J. *et al.* Disruption of G-quadruplex dynamicity by BRCA2 abrogation instigates phase separation and break-induced replication at telomeres. *Nucleic Acids Res* (2024).
2. In, S., Renck Nunes, P., Valador Fernandes, R. & Lingner, J. TERRA R-loops trigger a switch in telomere maintenance towards break-induced replication and PRIMPOL-dependent repair. *EMBO J* (2025).
3. Zhang, T. *et al.* Break-induced replication orchestrates resection-dependent template switching. *Nature* **619**, 201-208 (2023).
4. Cho, N.W., Dilley, R.L., Lampson, M.A. & Greenberg, R.A. Interchromosomal homology searches drive directional ALT telomere movement and synapsis. *Cell* **159**, 108-121 (2014).
5. Lee, J.J. *et al.* Disruption of G-quadruplex dynamicity by BRCA2 abrogation instigates phase separation and break-induced replication at telomeres. *Nucleic Acids Res* **52**, 5756-5773 (2024).
6. Kwon, M.S. *et al.* Brca2 abrogation engages with the alternative lengthening of telomeres via break-induced replication. *FEBS J* **286**, 1841-1858 (2019).
7. Min, J. *et al.* The breast cancer susceptibility gene BRCA2 is required for the maintenance of telomere homeostasis. *J Biol Chem* **287**, 5091-5101 (2012).
