# Supplementary material for: G-quadruplex homeostasis is a determinant of PARP inhibitor toxicity in BRCA2-deficient cells
Source: Nat Commun. 2026 Jul 3;17:8275. doi: 10.1038/s41467-026-75083-9 (PMC13470221; doi:10.1038/s41467-026-75083-9)
Supplement: Supplementary file 4 [file 41467_2026_75083_MOESM4_ESM.zip › Source_data_n values.pdf]

n values for Figures

Figure 1a – from three independent biological experiments (N=3)
<u>Untreated:</u>
U2OS: *n* = 273
HAPSTR1Δ.2: *n* = 241
HAPSTR1Δ.2 + FLAG-HA-HAPSTR1: *n* = 192
<u>PDS treated:</u>
U2OS: *n* = 213
HAPSTR1Δ.2: *n* = 208
HAPSTR1Δ.2 + FLAG-HA-HAPSTR1: *n* = 189

Figure 1b – from three independent biological experiments (N=3)
U2OS: *n* = 3
HAPSTR1Δ.2: *n* = 3
HAPSTR1Δ.2 + FLAG-HA-HAPSTR1: *n* = 3

Figure 1c – from three independent biological experiments (N=3)
<u>Untreated:</u>
U2OS: *n* = 219
HAPSTR1Δ.2: *n* = 172
HAPSTR1Δ.2 + FLAG-HA-HAPSTR1: *n* = 168
<u>PDS treated:</u>
U2OS: *n* = 219
HAPSTR1Δ.2: *n* = 220
HAPSTR1Δ.2 + FLAG-HA-HAPSTR1: *n* = 161

Figure 1d – from three independent biological experiments (N=3)
<u>Untreated:</u>
- EdU negative
U2OS: *n* = 180
HAPSTR1Δ.2: *n* = 194
- EdU positive
U2OS: *n* = 127
HAPSTR1Δ.2: *n* = 188
<u>DRB treated</u>:
- EdU negative
U2OS: *n* = 220
HAPSTR1Δ.2: *n* = 244
- EdU positive
U2OS: *n* = 159
HAPSTR1Δ.2: *n* = 204
<u>PDS treated:</u>
- EdU negative
U2OS: *n* = 138
HAPSTR1Δ.2: *n* = 182
- EdU positive
U2OS: *n* = 194
HAPSTR1Δ.2: *n* = 189
<u>DRB and PDS treated</u>:
- EdU negative
U2OS: *n* = 199
HAPSTR1Δ.2: *n* = 148
- EdU positive
U2OS: *n* = 197
HAPSTR1Δ.2: *n* = 198

Figure 1e – from four independent biological experiments (N=4)

Untreated:
U2OS: *n* = 4
HAPSTR1Δ.2: *n* = 4
HAPSTR1Δ.2 + FLAG-HA-HAPSTR1: *n* = 4
PDS treated:
U2OS: *n* = 4
HAPSTR1Δ.2: *n* = 4
HAPSTR1Δ.2 + FLAG-HA-HAPSTR1: *n* = 4

Figure 1f – from three independent biological experiments (N=3)
Untreated:
U2OS: *n* = 3
HAPSTR1Δ.2: *n* = 3
HAPSTR1Δ.2 + FLAG-HA-HAPSTR1: *n* = 3
PDS treated:
U2OS: *n* = 3
HAPSTR1Δ.2: *n* = 3
HAPSTR1Δ.2 + FLAG-HA-HAPSTR1: *n* = 3

Figure 2a – from four independent biological experiments (N=4)
Untreated:
U2OS: *n* = 4
HAPSTR1Δ.2: *n* = 4
Olaparib treated:
U2OS: *n* = 4
HAPSTR1Δ.2: *n* = 4
Untreated:
U2OS: *n* = 4
HAPSTR1Δ.3: *n* = 4
Olaparib treated:
U2OS: *n* = 4
HAPSTR1Δ.3: *n* = 4

Figure 2b – from three independent biological experiments (N=3)
EdU negative:
U2OS: *n* = 216
HAPSTR1Δ.2: *n* = 208
EdU positive:
U2OS: *n* = 219
HAPSTR1Δ.2: *n* = 153

Figure 2c – from three independent biological experiments (N=3)
PCNA-G4:
U2OS: *n* = 335
HAPSTR1Δ.2: *n* = 297
HAPSTR1Δ.2 + FLAG-HA-HAPSTR1: *n* = 328
Controls:
U2OS-PCNA: *n* = 269
HAPSTR1Δ.2-PCNA: *n* = 254

Figure 2d – from three independent biological experiments (N=3)
Untreated:
U2OS: *n* = 279
HAPSTR1Δ.2: *n* = 258
Olaparib treated:
U2OS: *n* = 223
HAPSTR1Δ.2: *n* = 280

Figure 2e – from three independent biological experiments (N=3)
PARP1-G4:
U2OS: *n* = 531
HAPSTR1Δ.2: *n* = 586
HAPSTR1Δ.2 + FLAG-HA-HAPSTR1: *n* = 369
Controls:
U2OS-PARP1: *n* = 336
HAPSTR1Δ.2-PARP1: *n* = 329
HAPSTR1Δ.2 + FLAG-HA-HAPSTR1-PARP1: *n* = 286
U2OS-G4: *n* = 94
HAPSTR1Δ.2-G4: *n* = 84

Figure 2f – from three independent biological experiments (N=3)
Untreated:
U2OS: *n* = 436
Olaparib treated:
U2OS: *n* = 334
Veliparib treated:
U2OS: *n* = 413

Figure 2g – from three independent biological experiments (N=3)
Untreated:
U2OS: *n* = 573
HAPSTR1Δ.2: *n* = 358
Olaparib treated:
U2OS: *n* = 375
HAPSTR1Δ.2: *n* = 367
Controls untreated:
U2OS-G4: *n* = 344
HAPSTR1Δ.2-G4: *n* = 365
Controls olaparib treated:
U2OS-G4: *n* = 381
HAPSTR1Δ.2-G4: *n* = 396

Figure 3a – from three independent biological experiments (N=3)
Untreated:
U2OS: n = 499
HAPSTR1Δ.2: n = 558
HAPSTR1Δ.3: n = 590
Olaparib treated:
U2OS: n = 555
HAPSTR1Δ.2: n = 738
HAPSTR1Δ.3: n = 693

Figure 3b - from three independent biological experiments (N=3)
Untreated:
U2OS: *n* = 212
HAPSTR1Δ.2: *n* = 212
HAPSTR1Δ.3: *n* = 214
Untreated and S1 digested:
U2OS: *n* = 216
HAPSTR1Δ.2: *n* = 221
HAPSTR1Δ.3: *n* = 235
Olaparib treated:
U2OS: *n* = 234
HAPSTR1Δ.2: *n* = 210
HAPSTR1Δ.3: *n* = 204
Olaparib and S1 treated:
U2OS: *n* = 235
HAPSTR1Δ.2: *n* = 211

HAPSTR1Δ.3: *n* = 222

Figure 3c - from three independent biological experiments (N=3)
Untreated:
HAPSTR1Δ.2: *n* = 318
HAPSTR1Δ.2 siPrimPol: *n* = 352
Untreated and subjected to S1:
HAPSTR1Δ.2: *n* = 462
HAPSTR1Δ.2 siPrimPol: *n* = 518
Olaparib treated:
HAPSTR1Δ.2: *n* = 412
HAPSTR1Δ.2 siPrimPol: *n* = 476
Olaparib and S1 treated:
HAPSTR1Δ.2: *n* = 499
HAPSTR1Δ.2 siPrimPol: *n* = 514

Figure 3d - from three independent biological experiments (N=3)
U2OS *n* = 3
U2OS siPrimPol *n* = 3
HAPSTR1Δ.2: *n* = 3
HAPSTR1Δ.2 siPrimPol: *n* = 3

Figure 3e - from three independent biological experiments (N=3)
Untreated:
HAPSTR1Δ.2: *n* = 169
Untreated and subjected to S1:
HAPSTR1Δ.2: *n* = 128
Olaparib treated:
HAPSTR1Δ.2: *n* = 198
Olaparib and S1 treated:
HAPSTR1Δ.2: *n* = 214
REV1i treated:
HAPSTR1Δ.2: *n* = 299
REV1i and S1 treated:
HAPSTR1Δ.2: *n* = 260
REV1i and Olaparib treated:
HAPSTR1Δ.2: *n* = 324
REV1i, Olaparib and S1 treated:
HAPSTR1Δ.2: *n* = 334

Figure 3f - from three independent biological experiments (N=3)
U2OS: *n* = 3
HAPSTR1Δ.2: *n* = 3
HAPSTR1Δ.2 + FLAG-HA-HAPSTR1: *n* = 3

Figure 3g - from three independent biological experiments (N=3)
Untreated:
U2OS: *n* = 365
HAPSTR1Δ.2: *n* = 403
HAPSTR1Δ.2 siPrimPol: *n* = 705
Olaparib treated:
U2OS: *n* = 446
HAPSTR1Δ.2: *n* = 385
HAPSTR1Δ.2 siPrimPol: *n* = 615
Controls:
anti-G4: *n* = 327
anti-BrdU: *n* = 270

Figure 4a - from three independent biological experiments (N=3)
U2OS *n* = 3
U2OS siBRCA2 *n* = 3
HAPSTR1Δ.2: *n* = 3
HAPSTR1Δ.2 siBRCA2: *n* = 3

Figure 4b - from three independent biological experiments (N=3)
U2OS: *n* = 255
HAPSTR1Δ.2: *n* = 166
U2OS siBRCA2: *n* = 183
HAPSTR1Δ.2 siBRCA2: *n* = 216

Figure 4c - from three independent biological experiments (N=3)
U2OS: *n* = 515
HAPSTR1Δ.2: *n* = 204
U2OS siBRCA2: *n* = 1123
HAPSTR1Δ.2 siBRCA2: *n* = 1938

Figure 4d - from three independent biological experiments (N=3)
<u>Veliparib:</u>
U2OS *n* = 3
U2OS siBRCA2 *n* = 3
HAPSTR1Δ.2: *n* = 3
HAPSTR1Δ.2 siBRCA2: *n* = 3
<u>Olaparib:</u>
U2OS *n* = 3
U2OS siBRCA2 *n* = 3
HAPSTR1Δ.2: *n* = 3
HAPSTR1Δ.2 siBRCA2: *n* = 3

Figure 4e - from three independent biological experiments (N=3)
<u>Untreated:</u>
U2OS: *n* = 263
U2OS siBRCA2: *n* = 447
HAPSTR1Δ.2: *n* = 220
HAPSTR1Δ.2 siBRCA2: *n* = 300
<u>Untreated and subjected to S1:</u>
U2OS: *n* = 420
U2OS siBRCA2: *n* = 448
HAPSTR1Δ.2: *n* = 294
HAPSTR1Δ.2 siBRCA2: *n* = 413
<u>Olaparib treated:</u>
U2OS: *n* = 275
U2OS siBRCA2: *n* = 303
HAPSTR1Δ.2: *n* = 227
HAPSTR1Δ.2 siBRCA2: *n* = 305
<u>Olaparib and S1 treated:</u>
U2OS: *n* = 270
U2OS siBRCA2: *n* = 454
HAPSTR1Δ.2: *n* = 291
HAPSTR1Δ.2 siBRCA2: *n* = 392

Figure 4f - from three independent biological experiments (N=3)
<u>Untreated:</u>
U2OS: *n* = 251
U2OS siBRCA2: *n* = 618
HAPSTR1Δ.2: *n* = 302
HAPSTR1Δ.2 siBRCA2: *n* = 517
<u>Olaparib treated:</u>
U2OS: *n* = 271

U2OS siBRCA2: *n* = 709
HAPSTR1Δ.2: *n* = 353
HAPSTR1Δ.2 siBRCA2: *n* = 798

Figure 4g - from three independent biological experiments (N=3)
Untreated:
U2OS: *n* = 356
U2OS siBRCA2: *n* = 358
HAPSTR1Δ.2: *n* = 401
HAPSTR1Δ.2 siBRCA2: *n* = 345
Olaparib treated:
U2OS: *n* = 281
U2OS siBRCA2: *n* = 256
HAPSTR1Δ.2: *n* = 387
HAPSTR1Δ.2 siBRCA2: *n* = 410

Figure 5a - from three independent biological experiments (N=3)
U2OS: *n* = 289
U2OS siBRCA2: *n* = 202
U2OS + FLAG-HA-HAPSTR1: *n* = 256
U2OS + FLAG-HA-HAPSTR1 siBRCA2: *n* = 311

Figure 5b - from three independent biological experiments (N=3)
U2OS: *n* = 3
U2OS siBRCA2: *n* = 3
U2OS + FLAG-HA-HAPSTR1: *n* = 3
U2OS + FLAG-HA-HAPSTR1 siBRCA2: *n* = 3

Figure 5d - from three independent biological experiments (N=3)
U2OS siCTRL: *n* = 3
U2OS siHUWE1: *n* = 3

Figure 5h - from three independent biological experiments (N=3)
U2OS: *n* = 271
U2OS siHUWE1: *n* = 283
U2OS siBRCA2: *n* = 282
U2OS siHUWE1 siBRCA2: *n* = 274
HAPSTR1Δ.2: *n* = 308
HAPSTR1Δ.2 siHUWE1: *n* = 269
HAPSTR1Δ.2 siBRCA2: *n* = 258
HAPSTR1Δ.2 siHUWE1 siBRCA2: *n* = 205

Figure 5i - from three independent biological experiments (N=3)
U2OS: *n* = 3
U2OS siHUWE1: *n* = 3
U2OS siBRCA2: *n* = 3
U2OS siHUWE1 siBRCA2: *n* = 3

n values for Supplementary Figures

Supplementary Fig 1c - from three independent biological experiments (N=3)
Untreated:
RPE1: *n*=454
RPE1-G5Δ.HAPSTR1: *n*=450
RPE1-G5Δ.HAPSTR1+HAPSTR1: *n*=444
PDS treated:
RPE1: *n*=591
RPE1-G5Δ.HAPSTR1: *n*=483
RPE1-G5Δ.HAPSTR1+HAPSTR1: *n*=417

Supplementary Fig 1d - from three independent biological experiments (N=3)
U2OS EdU+: *n*=230
U2OS EdU-: *n*=170
H*APSTR1Δ.2 EdU+*: *n*=207
H*APSTR1Δ.2 EdU-*: *n*=257

Supplementary Fig 1e - from three independent biological experiments (N=3)
U2OS: *n*=624
U2OS DRB+: *n*=424
U2OS PDS+: *n*=545
U2OS PDS+ DRB+: *n*=518
HAPSTR1: *n*=566
HAPSTR1 DRB+: *n*=492
HAPSTR1 PDS+: *n*=562
HAPSTR1 DRB+ PDS+: *n*=560

Supplementary Fig 2a - from three independent biological experiments (N=3)
*HASPTR1*Δ.2 + DRB: *n*=502
*HASPTR1*Δ.3 + DRB: *n*=416

Supplementary Fig 2b - from three independent biological experiments (N=3)
WT (4h PDS): *n*=250
*HASPTR1*Δ.2 (4h PDS): *n*=246
WT + DRB (4h PDS): *n*=258
*HASPTR1*Δ.2 + DRB (4h PDS): *n*=242

Supplementary Fig 2c - from three independent biological experiments (N=3)
Untreated:
U2OS: *n*=869
HAPSTR1Δ.2: *n*=801
PDS treated:
U2OS: *n*=585
HAPSTR1Δ.2: *n*=621

Supplementary Fig 2d - from three independent biological experiments (N=3)
U2OS_DRB-_PDS(0)_Edu-: *n*=170
HAPSTR1Δ.2_DRB-_PDS(0)_Edu-: *n*=138
U2OS_DRB+_PDS(0)_Edu-: *n*=221
HAPSTR1Δ.2_DRB+_PDS(0)_Edu-: *n*=238
U2OS_DRB-_PDS(0)_Edu+: *n*=186
HAPSTR1Δ.2_DRB-_PDS(0)_Edu+: *n*=259
U2OS_DRB+_PDS(0)_Edu+: *n*=153
HAPSTR1Δ.2_DRB+_PDS(0)_Edu+: *n*=189
U2OS_DRB-_PDS(50)_Edu-: *n*=208
HAPSTR1Δ.2_DRB-_PDS(50)_Edu-: *n*=221
U2OS_DRB+_PDS(50)_Edu-: *n*=232

HAPSTR1Δ.2_DRB+_PDS(50)_Edu-: *n*=200
U2OS_DRB-_PDS(50)_Edu+: *n*=175
HAPSTR1Δ.2_DRB-_PDS(50)_Edu+: *n*=167
U2OS_DRB+_PDS(50)_Edu+: *n*=119
HAPSTR1Δ.2_DRB+_PDS(50)_Edu+: *n*=219

Supplementary Fig 3b - from three independent biological experiments (N=2)
U2OS: *n*=2
HAPSTR1Δ.2: *n*=2

Supplementary Fig 4b - from three independent biological experiments (N=3)
U2OS Edu-: *n*= 216
U2OS Edu+: *n*= 219
HAPSTR1Δ.2 Edu-: *n*= 208
HAPSTR1Δ.2 Edu+: *n*=153
PARP1/2$^{-/-}$ Edu-: *n*=153
PARP1/2$^{-/-}$ Edu+: *n*=89

Supplementary Fig 5a - from three independent biological experiments (N=3)
U2OS *n*=260
HAPSTR1Δ.2 *n*=262
U2OS+Ola *n*=319
HAPSTR1Δ.2 +Ola *n*=251

Supplementary Fig 5b - from three independent biological experiments (N=3)
Untreated:
RPE1 UT: *n*=516
RPE1-G5Δ.*HAPSTR1* UT: *n*=426
Olaparib (5 uM) treated:
RPE1 + Ola: *n*=488
RPE1-G5Δ.*HAPSTR1* + Ola: *n*=485

Supplementary Fig 5c - from three independent biological experiments (N=3)
U2OS: *n*=345
HAPSTR1Δ.2: *n*=512
U2OS +PDS: *n*=410
HAPSTR1Δ.2 +PDS: *n*=283

Supplementary Fig 6b - from three independent biological experiments (N=3)
Untreated:
U2OS: *n*=320
HAPSTR1Δ.2: *n*=270
HAPSTR1Δ.3: *n*=322
Untreated and S1 digested:
U2OS: *n*=272
HAPSTR1Δ.2: *n*=291
HAPSTR1Δ.3: *n*=192
Olaparib treated:
U2OS: *n*=294
HAPSTR1Δ.2: *n*=303
HAPSTR1Δ.3: *n*=304
Olaparib and S1 treated:
U2OS: *n*=309
HAPSTR1Δ.2: *n*=315
HAPSTR1Δ.3: *n*=282

Supplementary Fig 6c - from three independent biological experiments (N=3)
Untreated:
HAPSTR1Δ.2: *n*=336

HAPSTR1Δ.2 siPrimPol: *n*=330
Untreated and subjected to S1:
HAPSTR1Δ.2: *n*=477
HAPSTR1Δ.2 siPrimPol: *n*=556
Olaparib treated:
HAPSTR1Δ.2: *n*=359
HAPSTR1Δ.2 siPrimPol: *n*=489
Olaparib and S1 treated:
HAPSTR1Δ.2: *n*=531
HAPSTR1Δ.2 siPrimPol: *n*=516

Supplementary Fig 6d - from three independent biological experiments (N=3)
Untreated:
HAPSTR1Δ.2: *n*=610
Untreated and subjected to S1:
HAPSTR1Δ.2: *n*=506
Olaparib treated:
HAPSTR1Δ.2: *n*=390
Olaparib and S1 treated:
HAPSTR1Δ.2: *n*=451
REV1i treated:
HAPSTR1Δ.2: *n*=518
REV1i and S1 treated:
HAPSTR1Δ.2: *n*=509
REV1i and Olaparib treated:
HAPSTR1Δ.2: *n*=571
REV1i, Olaparib and S1 treated:
HAPSTR1Δ.2: *n*=608

Supplementary Fig 6e - from three independent biological experiments (N=3)
Untreated:
U2OS: *n*=213
U2OS siBRCA2: *n*=447
HAPSTR1Δ.2: *n*=220
HAPSTR1Δ.2 siBRCA2: *n*=301
Untreated and subjected to S1:
U2OS: *n*=275
U2OS siBRCA2: *n*=498
HAPSTR1Δ.2: *n*=281
HAPSTR1Δ.2 siBRCA2: *n*=413
Olaparib treated:
U2OS: *n*=290
U2OS siBRCA2: *n*=278
HAPSTR1Δ.2: *n*=277
HAPSTR1Δ.2 siBRCA2: *n*=305
Olaparib and S1 treated:
U2OS: *n*=255
U2OS siBRCA2: *n*=454
HAPSTR1Δ.2: *n*=291
HAPSTR1Δ.2 siBRCA2: *n*=392

Supplementary Fig 7b - from three independent biological experiments (N=3)
RPE1+ siCTRL: *n*=269
RPE1-G5Δ.*HAPSTR1*+ siCTRL: *n*=343
RPE1+ siBRCA2: *n*=236
RPE1- G5Δ.*HAPSTR1*+ siBRCA2: *n*=174

Supplementary Fig 7c - from three independent biological experiments (N=3)
U2OS *n*=420
HAPSTR1Δ.2 *n*=375

U2OS siBRCA2 *n*=289
HAPSTR1Δ.2 siBRCA2 *n*=276

Supplementary Fig 7d - from three independent biological experiments (N=3)
RPE1+siCTR: *n*=3
RPE1+siBRCA2: *n*=3
RPE1-G5Δ.*HAPSTR1*+siCTRL: *n*=3
RPE1-G5Δ.*HAPSTR1*+siBRCA2: *n*=3

Supplementary Fig 8b - from three independent biological experiments (N=3)
U2OS: *n*=3
U2OS + FLAG-HA-HAPSTR1: *n*=3

Supplementary Fig 8d - from three independent biological experiments (N=3)
U2OS siBRCA2: *n*=399
U2OS siBRCA2_S1: *n*=559
U2OS siBRCA2 + oalp: *n*=493
U2OS siBRCA2 + olap_S1: *n*=511
HAPSTR1-OExp_siBRCA2: *n*=587
HAPSTR1-OExp_siBRCA2_S1: *n*=737
HAPSTR1-OExp_siBRCA2_OLAP: *n*=506
HAPSTR1-OExp_siBRCA2_OLAP_S1: *n*=752

U2OS siBRCA2: *n*=399
U2OS siBRCA2_S1: *n*=559
U2OS siBRCA2 + oalp: *n*=493
U2OS siBRCA2 + olap_S1: *n*=511
HAPSTR1-OExp_siBRCA2: *n*=587
HAPSTR1-OExp_siBRCA2_S1: *n*=737
HAPSTR1-OExp_siBRCA2_OLAP: *n*=506
HAPSTR1-OExp_siBRCA2_OLAP_S1: *n*=752
