# Supplementary material for: G-quadruplex homeostasis is a determinant of PARP inhibitor toxicity in BRCA2-deficient cells
Source: Nat Commun. 2026 Jul 3;17:8275. doi: 10.1038/s41467-026-75083-9 (PMC13470221; doi:10.1038/s41467-026-75083-9)
Supplement: Supplementary file 4 [file 41467_2026_75083_MOESM4_ESM.zip › Source_data_p_values.pdf]

p-values for Figures

Figure 1a

Tukey's multiple comparisons test	Summary	Adjusted P Value
U2OS vs HAPSTR1Δ.2	ns	0.8057
U2OS vs HAPSTR1Δ.2+HAPSTR1	ns	0.9787
U2OS vs PDS U2OS	****	<0.0001
PDS U2OS vs PDS HAPSTR1Δ.2	****	<0.0001
PDS HAPSTR1Δ.2 vs PDS HAPSTR1Δ.2+HAPSTR1	****	<0.0001

Figure 1b

Šídák's multiple comparisons test	Summary	Adjusted P Value
U2OS vs. HAPSTR1Δ.2	***	0.0003
HAPSTR1Δ.2 vs. HAPSTR1Δ.2+HAPSTR1	***	0.0001

Figure 1c

Dunnett's T3 multiple comparisons test	Summary	Adjusted P Value
U2OS vs HAPSTRΔ.1	**	0.0017
HAPSTR1Δ.2+ vs HAPSTR1Δ.2+HAPSTR1	****	<0.0001
PDS U2OS vs PDS HAPSTR1Δ.2	****	<0.0001
PDS HAPSTR1Δ.2 vs PDS HAPSTR1Δ.2+HAPSTR1	****	<0.0001

Figure 1d

Dunn's multiple comparisons test	Summary	Adjusted P Value
HAPSTR1Δ.2_DRB+_PDS(50)_EdU+ vs. U2OS DRB+_PDS(50)_EdU+	***	0.0004
HAPSTR1Δ.2_DRB-_PDS(50)_EdU+ vs. HAPSTR1Δ.2_DRB+ PDS(50) EdU+	ns	>0.9999
U2OS _DRB-_PDS(50)_EdU+ vs. HAPSTR1Δ.2_DRB-_PDS(50)_EdU+	****	<0.0001
U2OS _DRB-_PDS(50)_EdU+ vs. HAPSTR1Δ.2_DRB-_PDS(50)_EdU+	ns	0.1533
U2OS _DRB-_PDS(50)_EdU- vs. U2OS _DRB+_PDS(50)_EdU-	ns	>0.9999
U2OS _DRB-_PDS(0) EdU- vs. HAPSTR1Δ.2_DRB-_PDS(0) EdU-	ns	>0.9999
U2OS _DRB-_PDS(0)_EdU+ vs. HAPSTR1Δ.2_DRB-_PDS(0)_EdU+	ns	0.0874
HAPSTR1Δ.2_DRB-_PDS(0)_EdU+ vs. HAPSTR1Δ.2_DRB-_PDS(50) EdU-	****	<0.0001
U2OS _DRB+_PDS(0) _EdU+ vs. HAPSTR1Δ.2_DRB+_PDS(0)_Edu+	**	0.0017

Figure 1e

Tukey's multiple comparisons test	Summary	Adjusted P Value
PDS U2OS vs PDS HAPSTRΔ.1	****	<0.0001
PDS HAPSTRΔ.1 vs PDS HAPSTRΔ.1+ HAPSTR1	*	0.0157

Figure 1f

Tukey's multiple comparisons test	Summary	Adjusted P Value
U2OS vs PDS U2OS	ns	0.9910
U2OS vs HAPSTR1Δ.2+HAPSTR1	ns	0.3449
PDS U2OS vs PDS HAPSTR1Δ.2	**	0.0099
PDS HAPSTR1Δ.2 vs PDS HAPSTR1Δ.2+HAPSTR1	**	0.0077

Figure 2a

Tukey's multiple comparisons test	Summary	Adjusted P Value
U2OS vs HAPSTR1Δ.2	*	0.0177
U2OS vs. U2OS + OLAPARIB	*	0.0175
U2OS vs. HAPSTR1Δ.2+OLAPARIB	**	0.0011
HAPSTR1Δ.2 vs. HAPSTR1Δ.2 + OLAPARIB	**	0.0054
U2OS + OLAPARIB vs. HAPSTR1Δ.2 + OLAPARIB	**	0.0044

Tukey's multiple comparisons test	Summary	Adjusted P Value
U2OS vs. HAPSTR1Δ.3	**	0.0094
U2OS vs. HAPSTR1Δ.3 + OLAPARIB	**	0.0037
U2OS + OLAPARIB vs. HAPSTR1Δ.3 + OLAPARIB	*	0.0174
HAPSTR1Δ.3 vs. HAPSTR1Δ.3 + OLAPARIB	**	0.0098

Figure 2b

Dunnett's T3 multiple comparisons test	Summary	Adjusted P Value
U2OS Edu- vs. U2OS Edu+	****	<0.0001
U2OS Edu- vs. HAPSTR1Δ.2 Edu-	ns	0.5373
U2OS Edu+ vs. HAPSTR1Δ.2 Edu+	****	<0.0001

Figure2c

Tukey's multiple comparisons test	Summary	Adjusted P Value
U2OS (G4-PCNA) vs HAPSTR1Δ.2 (G4-PCNA)	****	<0.0001
HAPSTR1Δ.2 (G4-PCNA) vs. HAPSTR1Δ.2 + HAPSTR1 (G4-PCNA)	****	<0.0001
U2OS (PCNA) vs. HAPSTR1Δ.2 (PCNA)	ns	0.9998

Figure 2d

Tukey's multiple comparisons test	Summary	Adjusted P Value
U2OS UT vs. WT OLAPARIB	****	<0.0001
U2OS UT vs. HAPSTR1Δ.2 UT	****	<0.0001
U2OS OLAPARIB vs. HAPSTR1Δ.2 OLAPARIB	****	<0.0001
HAPSTR1Δ.2 UT vs. HAPSTR1Δ.2 OLAPARIB	****	<0.0001

Figure 2e

Dunn's multiple comparisons test	Summary	Adjusted P Value
U2OS (G4-PARP1) vs. HAPSTR1Δ.2 (G4-PARP1)	***	0.0009
U2OS (G4-PARP)1 vs. HAPSTR1Δ.2+HAPSTR1 (G4-PARP1)	ns	0.0526
HAPSTR1Δ.2 (G4-PARP1) vs. HAPSTR1Δ.2+HAPSTR1 (G4-PARP1)	****	<0.0001
U2OS (PARP1-Only) vs. HAPSTR1Δ.2+HAPSTR1 (PARP1-Only)	ns	0.5487
U2OS (PARP1-Only) vs. U2OS UT (G4-only)	ns	0.7093
HAPSTR1Δ.2 (PARP1-Only) vs. HAPSTR1Δ.2 UT PLA (G4-only)	ns	0.6339

Figure 2f

Holm-Šídák's multiple comparisons test	Summary	Adjusted P Value
U2OS (HAPSTR1-G4) vs. U2OS-VELIPARIB (HAPSTR1-G4)	****	<0.0001
U2OS (HAPSTR1-G4) vs. U2OS OLAPARIB (HAPSTR1-G4)	*	0.0316
U2OS -VELIPARIB (HAPSTR1-G4) vs. U2OS OLAPARIB (HAPSTR1-G4)	****	<0.0001

Figure 2g

Dunn's multiple comparisons test	Summary	Adjusted P Value
U2OS UT (HAPSTR1-G4) vs. PARP1/PARP2 KO (HAPSTR1-G4)	****	<0.0001
U2OS UT (HAPSTR1-G4) vs. U2OS -OLAPARIB (HAPSTR1-G4)	****	<0.0001
PARP1/PARP2 KO (HAPSTR1-G4) vs. U2OS -OLAPARIB (HAPSTR1-G4)	****	<0.0001
PARP1/PARP2 KO (HAPSTR1-G4) vs. PARP1/PARP2 KO OLAPARIB (HAPSTR1-G4)	ns	>0.9999

Figure 3a

Dunnett's T3 multiple comparisons test	Summary	Adjusted P Value
U2O2 vs. HAPSTR1Δ.2	***	0.0004
U2O2 vs. HAPSTR1Δ.3	****	<0.0001
U2O2 vs. U2O2 + OLAPARIB	ns	>0.9999
HAPSTR1Δ.2 vs. HAPSTR1Δ.2 + OLAPARIB	****	<0.0001
HAPSTR1Δ.3 vs. HAPSTR1Δ.3 + OLAPARIB	****	<0.0001

Figure 3b

**Dunnett's T3 multiple comparisons test**	**Summary**	**Adjusted P Value**
U2O2 vs. U2O2 + S1	ns	0.8725
HAPSTR1Δ.2 vs. HAPSTR1Δ.2 + S1	****	<0.0001
HAPSTR1Δ.3 vs. HAPSTR1Δ.3 + S1	****	<0.0001
U2O2 + OLAPARIB vs. U2O2 + S1	***	0.0001
HAPSTR1Δ.2 + OLAPARIB vs. HAPSTR1Δ.2 + OLAPARIB + S1	****	<0.0001
HAPSTR1Δ.3 + OLAPARIB vs. HAPSTR1Δ.3 + OLAPARIB + S1	****	<0.0001
U2O2 + S1 vs. U2O2 + OLAPARIB + S1	ns	>0.9999
HAPSTR1Δ.2 + S1 vs. HAPSTR1Δ.2 + OLAPARIB + S1	****	<0.0001
HAPSTR1Δ.3 + S1 vs. HAPSTR1Δ.3 + OLAPARIB + S1	***	0.0002

Figure 3c

**Dunn's multiple comparisons test**	**Summary**	**Adjusted P Value**
HAPSTR1Δ.2 vs. HAPSTR1Δ.2 _S1	****	<0.0001
HAPSTR1Δ.2 + OLAPARIB vs. HAPSTR1Δ.2 + OLAPARIB_S1	****	<0.0001
HAPSTR1Δ.2_siPrimPol vs. HAPSTR1Δ.2_siPrimPol_S1	ns	>0.9999
HAPSTR1Δ.2_siPrimPol+ OLAPARIB vs. HAPSTR1Δ.2_siPrimPol+ OLAPARIB_S1	ns	>0.9999

Figure 3d

**Tukey's multiple comparisons test**	**Summary**	**Adjusted P Value**
HAPSTR1Δ.2 vs. HAPSTR1Δ.2 siPrimPol	**	0.0055
U2OS vs. U2OS siPrimPol	ns	0.8594

Figure 3e

**Dunn's multiple comparisons test**	**Summary**	**Adjusted P Value**
HAPSTR1Δ.2 vs. HAPSTR1Δ.2 _S1	****	<0.0001
HAPSTR1Δ.2 + OLAPARIB vs. HAPSTR1Δ.2 + OLAPARIB_S1	****	<0.0001
HAPSTR1Δ.2 + OLAPARIB vs. HAPSTR1Δ.2_REV1i + OLAPARIB_S1	****	<0.0001
HAPSTR1Δ.2 _S1 vs. HAPSTR1Δ.2_REV1i_S1	ns	>0.9999
HAPSTR1Δ.2 + OLAPARIB_S1 vs. HAPSTR1Δ.2_REV1i + OLAPARIB_S1	**	0.0013
HAPSTR1Δ.2_REV1i vs. HAPSTR1Δ.2_REV1i_S1	ns	>0.9999

Figure 3f

**Šídák's multiple comparisons test**	**Summary**	**Adjusted P Value**
U2OS vs. HAPSTR1Δ.2	**	0.0035
U2OS vs HAPSTR1Δ.2+HAPSTR1	ns	0.0535

Figure 3g

**Dunn's multiple comparisons test**	Summary	Adjusted P Value
U2O2 vs. U2O2 + OLAPARIB	****	<0.0001
U2O2 vs. HAPSTR1Δ.2	****	<0.0001
U2O2 + OLAPARIB vs. HAPSTR1Δ.2 + OLAPARIB	****	<0.0001
HAPSTR1Δ.2 vs. HAPSTR1Δ.2 + OLAPARIB	****	<0.0001
HAPSTR1Δ.2 vs. HAPSTR1Δ.2 siPrimPol	****	<0.0001
HAPSTR1Δ.2 + OLAPARIB vs. HAPSTR1Δ.2 siPrimPol + OLAPARIB	****	<0.0001
G4 vs. BrdU	ns	>0.9999

Figure 4a

**Tukey's multiple comparisons test**	**Summary**	**Adjusted P Value**
U2OS siCTRL vs. U2OS siBRCA2	**	0.0086
U2OS siCTRL vs. HAPSTR1Δ.2 siCTRL	**	0.0023
U2OS siCTRL vs. HAPSTR1Δ.2 siBRCA2	****	<0.0001
U2OS siBRCA2 vs. HAPSTR1Δ.2 siCTRL	ns	0.9657
U2OS siBRCA2 vs. HAPSTR1Δ.2 2 siBRCA2	**	0.0025

HAPSTR1Δ.2 siCTRL vs. HAPSTR1Δ.2 siBRCA2	**	0.0093

Figure 4b

**Dunnett's T3 multiple comparisons test**	**Summary**	**Adjusted P Value**
U2OS siCTRL vs. HAPSTR1Δ.2 siCTRL	****	<0.0001
U2OS siCTRL vs. U2OS siBRCA2	***	0.0003
HAPSTR1Δ.2 siCTRL vs. U2OS siBRCA2	ns	0.2564
U2OS siBRCA2 vs.HAPSTR1Δ.2 siBRCA2	****	<0.0001

Figure 4c

**Tukey's multiple comparisons test**	**Summary**	**Adjusted P Value**
U2OS siCTRL vs. HAPSTR1Δ.2 siCTRL	****	<0.0001
U2OS siCTRL vs. U2OS siBRCA2	****	<0.0001
HAPSTR1Δ.2 siCTRL vs. HAPSTR1Δ.2 siBRCA2	****	<0.0001
U2OS siBRCA2 vs. HAPSTR1Δ.2 siBRCA2	****	<0.0001

Figure 4d

**Tukey's multiple comparisons test**	**Summary**	**Adjusted P Value**
U2OS vs. U2OS siBRCA2	**	0.0017
U2OS vs. HAPSTR1Δ.2+siCTRL	***	0.0004
HAPSTR1Δ.2+siCTRL vs. HAPSTR1Δ.2+siBRCA2	***	0.0005

**Tukey's multiple comparisons test**	**Summary**	**Adjusted P Value**
U2OS siCTRL vs. U2OS+siBRCA2	****	<0.0001
U2OS siCTRL vs. *HAPSTR1Δ.2*siCTRL	***	0.0009
U2OS+siBRCA2 vs. *HAPSTR1Δ.2*+siBRCA2	**	0.0081

Figure 4e

**Dunn's multiple comparisons test**	**Summary**	**Adjusted P Value**
U2O2 vs. U2O2_S1	ns	0.1835
U2OS + OLAPARIB vs. U2OS + OLAPARIB_S1	ns	>0.9999
U2O2_S1 vs. U2O2_siBRCA2_S1	ns	>0.9999
U2OS + OLAPARIB_S1 vs. U2OS_siBRCA2 + OLAPARIB_S1	****	<0.0001
U2O2_siBRCA2 vs. U2O2_siBRCA2_S1	*	0.0268
U2OS_siBRCA2+ OLAPARIB vs. U2OS_siBRCA2 + OLAPARIB_S1	****	<0.0001
U2O2_siBRCA2_S1 vs. U2OS_siBRCA2 + OLAPARIB_S1	****	<0.0001
HAPSTR1Δ.2 vs. HAPSTR1Δ.2 _S1	****	<0.0001
HAPSTR1Δ.2 + OLAPARIB vs. HAPSTR1Δ.2 + OLAPARIB _S1	****	<0.0001
HAPSTR1Δ.2 _S1 vs. HAPSTR1Δ.2_siBRCA2_S1	ns	0.1767
HAPSTR1Δ.2 + OLAPARIB_S1 vs. HAPSTR1Δ.2_siBRCA2 + OLAPARIB _S1	ns	>0.9999
HAPSTR1Δ.2_siBRCA2 vs. HAPSTR1Δ.2_siBRCA2_S1	****	<0.0001
HAPSTR1Δ.2_siBRCA2+ OLAPARIB vs. HAPSTR1Δ.2_siBRCA2 + OLAPARIB_S1	**	0.0070
HAPSTR1Δ.2_siBRCA2_S1 vs. HAPSTR1Δ.2_siBRCA2 + OLAPARIB_S1	****	<0.0001

Figure 4f

**Dunnett's T3 multiple comparisons test**	**Summary**	**Adjusted P Value**
U2OS vs. U2OS siBRCA2 + OLAPARIB	****	<0.0001
U2OS vs. HAPSTR1Δ.2	****	<0.0001
U2OS + OLAPARIB vs. HAPSTR1Δ.2 siBRCA2 + OLAPARIB	****	<0.0001
U2OS siBRCA2 vs. U2OS siBRCA2 + OLAPARIB	**	0.0035
HAPSTR1Δ.2 vs. HAPSTR1Δ.2 + OLAPARIB	****	<0.0001
HAPSTR1Δ.2 vs. HAPSTR1Δ.2 siBRCA2	****	<0.0001
HAPSTR1Δ.2 + OLAPARIB vs. HAPSTR1Δ.2 siBRCA2 + OLAPARIB	****	<0.0001
HAPSTR1Δ.2 siBRCA2 vs. HAPSTR1Δ.2 siBRCA2 + OLAPARIB	**	0.0029

Figure 4g

Dunn's multiple comparisons test	Summary	Adjusted P Value
U2OS vs. U2OS + OLAPARIB	ns	0.8158
U2OS vs. U2OS siBRCA2	****	<0.0001
HAPSTR1Δ.2 vs. HAPSTR1Δ.2 + OLAPARIB	****	<0.0001
HAPSTR1Δ.2 vs. HAPSTR1Δ.2 siBRCA2	***	0.0001
HAPSTR1Δ.2 siBRCA2 vs. HAPSTR1Δ.2 siBRCA2+ OLAPARIB	****	<0.0001

Figure 5a

Tukey's multiple comparisons test	Summary	Adjusted P Value
U2OS siCTRL vs. U2OS siBRCA2	****	<0.0001
U2OS siBRCA2 vs. U2OS + FLAG-HA-HAPSTR1 siBRCA2	****	<0.0001

Figure 5b

Tukey's multiple comparisons test	Summary	Adjusted P Value
U2OS siCTRL vs U2OS + FLAG-HA-HAPSTR1 siCTRL	*	0.0126
U2OS siCTRL vs. U2OS + FLAG-HA-HAPSTR1 + siBRCA2	ns	0.0790
U2OS siBRCA2 vs. U2OS + FLAG-HA-HAPSTR1 + siBRCA2	***	0.0004

Figure 5h

Uncorrected Dunn's test	Summary	Individual P Value
siCTRL U2OS vs. siBRCA2 U2OS	****	<0.0001
siBRCA2 U2OS vs. siBRCA2 + siHUWE1 U2OS	****	<0.0001
siBRCA2 + siHUWE1 U2OS vs. siBRCA2 + siHUWE1 HAPSTR1Δ.2	****	<0.0001
siBRCA2 HAPSTR1Δ.2 vs. siBRCA2 + siHUWE1 HAPSTR1Δ.2	ns	0.8116

Figure 5i

Tukey's multiple comparisons test	Summary	Adjusted P Value
U2OS siCTRL vs. U2OS siCTRL+ siHUWE1	ns	0.6627
U2OS siBRCA2 vs. U2OS+siBRCA2+ siHUWE1	***	0.0010

p-values for Supplementary Figures

Supplementary Figure 1c

Dunnett's T3 multiple comparisons test	Summary	Adjusted P Value
RPE1 vs. G5ΔHAPSTR1	****	<0.0001
G5Δ.HAPSTR1 vs. G5ΔHAPSTR1+HAPSTR1	**	0.0082
G5ΔHAPSTR1 vs. G5ΔHAPSTR1+PDS	****	<0.0001
RPE1 PDS vs. G5ΔHAPSTR1+PDS	****	<0.0001
G5ΔHAPSTR1+PDS vs. G5ΔHAPSTR1+HAPSTR1+	****	<0.0001

Supplementary Figure 1d

Dunn's multiple comparisons test	Summary	Adjusted P Value
U2OS EdU+ve vs. U2OS EdU-ve	ns	>0.9999
HAPSTR1Δ.2 EdU+ve vs. HAPSTR1Δ.2 EdU-ve	ns	>0.9999
U2OS EdU-ve vs. HAPSTR1Δ.2 EdU-ve	****	<0.0001
U2OS EdU+ve vs. HAPSTR1Δ.2 EdU+ve	****	<0.0001

Supplementary Figure 1e

Dunn's multiple comparisons test	Summary	Adjusted P Value
U2OS vs. U2OS + DRB	****	<0.0001
U2OS vs. HAPSTR1Δ.2	ns	>0.9999
U2OS + PDS vs. U2OS + PDS + DRB	****	<0.0001
U2OS + PDS vs. HAPSTR1Δ.2 + PDS	****	<0.0001
HAPSTR1Δ.2 vs. HAPSTR1Δ.2 + DRB	****	<0.0001
HAPSTR1Δ.2 + PDS vs. HAPSTR1Δ.2 + DRB + PDS	****	<0.0001

Supplementary Figure 2a

Tukey's multiple comparisons test	Summary	Adjusted P Value
*HASPTR1*Δ.2 vs. *HASPTR1*Δ.2 + DRB	ns	>0.9999

Supplementary Figure 2b

Dunn's multiple comparisons test	Summary	Adjusted P Value
WT (4h PDS) vs. *HASPTR1*Δ.2 (4h PDS)	****	<0.0001
*HASPTR1*Δ.2 (4h PDS) vs. *HASPTR1*Δ.2 + DRB (4h PDS)	ns	>0.9999

Supplementary Figure 2c

Dunn's multiple comparisons test	Summary	Adjusted P Value
U2OS-UT vs. HAPSTR1Δ.2-UT	****	<0.0001
U2OS-UT vs. U2OS+PDS	****	<0.0001
HAPSTR1Δ.2-UT vs. HAPSTR1Δ.2+PDS	****	<0.0001
U2OS+PDS vs. HAPSTR1Δ.2+PDS	****	<0.0001

Supplementary Figure 2d

Dunn's multiple comparisons test	Summary	Adjusted P Value
HAPSTR1Δ.2_DRB+_PDS(0)_Edu+ vs. HAPSTR1Δ.2_DRB-_PDS(0)_Edu+	*	0.0385
U2OS_DRB+_PDS(0)_Edu+ vs. HAPSTR1Δ.2_DRB+_PDS(0)_Edu+	ns	0.4753
U2OS _DRB-_PDS(0)_Edu+ vs. HAPSTR1Δ.2_DRB-_PDS(0)_Edu+	*	0.0185
**Dunn's multiple comparisons test**	**Summary**	**Adjusted P Value**
U2OS _DRB-_PDS(50)_Edu+ vs. HAPSTR1Δ.2_DRB-_PDS(50)_Edu+	**	0.0049
HAPSTR1Δ.2_DRB-_PDS(50)_Edu+ vs. HAPSTR1Δ.2_DRB+_PDS(50)_Edu+	**	0.0054
U2OS _DRB+_PDS(50)_Edu+ vs. HAPSTR1Δ.2_DRB+_PDS(50)_Edu+	**	0.0074
**Mann Whitney test**	**Summary**	**P Value**
HAPSTR1Δ.2_DRB-_PDS(0)_Edu- vs. U2OS_DRB-_PDS(0)_Edu-	***	0.0005

Supplementary Figure 4b

Dunnett's T3 multiple comparisons test	Summary	Adjusted P Value
U2OS Edu-ve vs. U2OS Edu+ve	****	<0.0001
U2OS Edu-ve vs. HAPSTR1Δ.2 Edu-ve	ns	0.8523
U2OS Edu-ve vs. PARP1/2$^{-/-}$ Edu-ve	ns	0.6044
U2OS Edu+ve vs. HAPSTR1Δ.2_Edu+ve	****	<0.0001
U2OS Edu+ve vs. PARP1/2$^{-/-}$ Edu+ve	****	<0.0001

Supplementary Figure 5a

Non-telomeric G4s foci analysis

Dunnett's T3 multiple comparisons test	Summary	Adjusted P Value
U2OS Non-telomeric G4s foci (per cell) vs. HAPSTR1Δ.2 Non-telomeric G4s loci	**	0.0072
U2OS Non-telomeric G4s foci vs. U2OS+Ola Non-telomeric G4s foci	*	0.0184
U2OS Non-telomeric G4s foci vs. HAPSTR1Δ.2 +Ola Non-telomeric G4s foci	****	<0.0001
HAPSTR1Δ.2 Non-telomeric G4s foci vs. HAPSTR1Δ.2 +Ola Non-telomeric G4s foci	ns	0.9702
HAPSTR1Δ.2 Non-telomeric G4s foci vs. HAPSTR1Δ.2 +Ola Non-telomeric G4s foci	***	0.0004
U2OS+Ola non-telomeric G4s foci vs. HAPSTR1Δ.2+Ola non-telomeric G4s foci	****	<0.0001

Telomeric G4s foci analysis

Dunnett's T3 multiple comparisons test	Summary	Adjusted P Value
U2OS No. of Telomeric G4s vs. HAPSTR1Δ.2 No. of Telomeric G4s	*	0.0182
U2OS No. of Telomeric G4s vs. U2OS+Ola No. of Telomeric G4s	****	<0.0001
U2OS No. of Telomeric G4s vs. HAPSTR1Δ.2+Ola No. of Telomeric G4s	****	<0.0001
HAPSTR1Δ.2 No. of Telomeric G4s vs. U2OS+Ola No. of Telomeric	*	0.0343
HAPSTR1Δ.2 No. of Telomeric G4s vs. HAPSTR1Δ.2+Ola No. of Telomeric G4s	****	<0.0001
U2OS+Ola No. of Telomeric G4s vs. HAPSTR1Δ.2+Ola No. of Telomeric G4s	*	0.0273

Supplementary Figure 5b

Tukey's multiple comparisons test	Summary	Adjusted P Value
RPE1 UT vs. G5. HAPSTR1 UT	****	<0.0001
G5.HAPSTR1 UT vs. G5.HAPSTR1 5uM Ola	***	0.0005
RPE1 5uM Ola vs. G5.HAPSTR1 5uM Ola	****	<0.0001

Supplementary Figure 6b

Dunnett's T3 multiple comparisons test	Summary	Adjusted P Value
U2O2 vs. U2O2 + S1	ns	0.6000
HAPSTR1Δ.2 vs. HAPSTR1Δ.2 +S1	****	<0.0001
HAPSTR1Δ.3 vs. HAPSTR1Δ.3 + S1	****	<0.0001
U2O2 + OLAPARIB vs. U2O2 + OLAPARIB + S1	ns	0.1395
HAPSTR1Δ.2 + OLAPARIB vs. HAPSTR1Δ.2 + OLAPARIB + S1	****	<0.0001
HAPSTR1Δ.3 + OLAPARIB vs. HAPSTR1Δ.3 + OLAPARIB + S1	****	<0.0001
U2O2 + S1 vs. U2O2 + OLAPARIB + S1	ns	>0.9999
HAPSTR1Δ.2 +S1 vs. HAPSTR1Δ.2 + OLAPARIB + S1	**	0.0041
HAPSTR1Δ.3 + S1 vs. HAPSTR1Δ.3 + OLAPARIB + S1	***	0.0003

Supplementary Figure 6c

Dunn's multiple comparisons test	Summary	Adjusted P Value
HAPSTR1Δ.2 vs. HAPSTR1Δ.2 _S1	****	<0.0001
HAPSTR1Δ.2 + OLAPARIB vs. HAPSTR1Δ.2 + OLAPARIB_S1	****	<0.0001
HAPSTR1Δ.2_siPrimPol vs. HAPSTR1Δ.2_siPrimPol_S1	ns	>0.9999
HAPSTR1Δ.2_siPrimPol+ OLAP vs. HAPSTR1Δ.2_siPrimPol+ OLAPARIB_S1	ns	0.5591

Supplementary Figure 6d

Dunn's multiple comparisons test	Summary	Adjusted P Value
HAPSTR1Δ.2 vs. HAPSTR1Δ.2 _S1	****	<0.0001

HAPSTR1Δ.2 + OLAPARIB vs. HAPSTR1Δ.2 + OLAPARIB_S1	****	<0.0001
HAPSTR1Δ.2 _S1 vs. HAPSTR1Δ.2_REV1i_S1	ns	>0.9999
HAPSTR1Δ.2_REV1i vs. HAPSTR1Δ.2_REV1i_S1	*	0.0363
HAPSTR1Δ.2_REV1i + OLAPARIB vs. HAPSTR1Δ.2_REV1i + OLAPARIB_S1	****	<0.0001
HAPSTR1Δ.2_REV1i_S1 vs. HAPSTR1Δ.2_REV1i + OLAPARIB_S1	****	<0.0001

Supplementary Figure 6e

Dunn's multiple comparisons test	Summary	Adjusted P Value
U2O2 vs. U2O2_S1	ns	>0.9999
U2OS + OLAP vs. U2OS + OLAP_S1	ns	>0.9999
U2O2_S1 vs. HAPSTR1Δ.2_siBRCA2_S1	**	0.0082
U2OS + OLAP_S1 vs. U2OS_siBRCA2+ OLAP_S1	****	<0.0001
U2O2_siBRCA2 vs. U2O2_siBRCA2_S1	*	0.0495
U2OS_siBRCA2+ OLAP vs. U2OS_siBRCA2+ OLAP_S1	****	<0.0001
U2O2_siBRCA2_S1 vs. U2OS_siBRCA2+ OLAP_S1	****	<0.0001
HAPSTR1Δ.2 vs. HAPSTR1Δ.2 + OLAP	ns	0.2031
HAPSTR1Δ.2 + OLAP vs. HAPSTR1Δ.2 + OLAP_S1	**	0.0031
HAPSTR1Δ.2 + OLAP_S1 vs. HAPSTR1Δ.2_siBRCA2+ OLAP_S1	ns	>0.9999
HAPSTR1Δ.2_siBRCA2 vs. HAPSTR1Δ.2_siBRCA2_S1	**	0.0048
HAPSTR1Δ.2_siBRCA2+ OLAP vs. HAPSTR1Δ.2_siBRCA2+ OLAP_S1	****	<0.0001
HAPSTR1Δ.2_siBRCA2_S1 vs. HAPSTR1Δ.2_siBRCA2+ OLAP_S1	**	

Supplementary Figure 7b

Dunnett's T3 multiple comparisons test	Summary	Adjusted P Value
RPE1+ siCTRL vs. G5Δ.HAPSTR1+ siCTRL	****	<0.0001
RPE1+ siCTRL vs. RPE1+ siBRCA2	*	0.0300
G5Δ.HAPSTR1 + siCTRL vs. RPE1+ siBRCA2	ns	0.1116
G5Δ.HAPSTR1 + siCTRL vs. G5Δ.HAPSTR1 + siBRCA2	****	<0.0001
RPE1+ siBRCA2 vs. G5Δ.HAPSTR1 + siBRCA2	****	<0.0001

Supplementary Figure 7c
Telomeric G4s foci analysis

Dunn's multiple comparisons test	Summary	Adjusted P Value
U2OS Telomeric G4s foci vs. HAPSTR1Δ.2 Telomeric G4s foci	***	0.0005
U2OS Telomeric G4s foci vs. U2OS+siBRCA2 Telomeric G4s foci	**	0.0084
U2OS Telomeric G4s foci vs. HAPSTR1Δ.2+siBRCA2 Telomeric G4s foci	****	<0.0001
HAPSTR1Δ.2 Telomeric G4s foci vs. U2OS+siBRCA2 Telomeric G4s foci	ns	>0.9999
HAPSTR1Δ.2 Telomeric G4s foci vs. HAPSTR1Δ.2+siBRCA2 Telomeric G4s foci	ns	0.8678
U2OS+siBRCA2 Telomeric G4s foci vs. HAPSTR1Δ.2+siBRCA2 Telomeric G4s foci	ns	0.7808

Non-telomeric G4s foci analysis

Dunn's multiple comparisons test	Summary	Adjusted P Value
U2OS non-Telomeric G4 foci vs. HAPSTR1Δ.2 non-Telomeric G4 foci	****	<0.0001
U2OS Telomeric G4s foci vs. U2OS+siBRCA2 Telomeric G4s foci	***	0.0002
U2OS non-Telomeric G4 foci vs. HAPSTR1Δ.2+siBRCA2 non-Telomeric G4 foci	****	<0.0001
HAPSTR1Δ.2 non-Telomeric G4 foci vs. siBRCA2 non-Telomeric G4 foci	ns	>0.9999
HAPSTR1Δ.2 non-Telomeric G4 foci vs. HAPSTR1Δ.2+siBRCA2 non-Telomeric G4 foci	*	0.0181
siBRCA2 non-Telomeric G4 foci vs. HAPSTR1Δ.2+siBRCA2 non-Telomeric G4 foci	*	0.0427

Supplementary Figure 7d

Tukey's multiple comparisons test	Summary	Adjusted P Value
RPE1+siCTR vs. RPE1+siBRCA2	****	<0.0001
RPE1+siCTR vs. G5Δ.*HAPSTR1*+siCTRL	****	<0.0001
RPE1+siBRCA2 vs. G5Δ.*HAPSTR1*+siBRCA2	*	0.0488
G5Δ.*HAPSTR1*+siCTRL vs. G5Δ.*HAPSTR1*+siBRCA2	***	0.0001

Supplementary Figure 8b

Unpaired *t* test	Summary	Adjusted P Value
U2OS Telomeric G4 foci vs. HAPSTR1Δ.2 Telomeric G4 foci	*	0.0391

Supplementary Figure 8d

Dunn's multiple comparisons test	Summary	Adjusted P Value
HAPSTR1-OExp_siBRCA2 vs. HAPSTR1-OExp_siBRCA2_S1	***	0.0003
HAPSTR1-OExp_siBRCA2_S1 vs. HAPSTR1-OExp_siBRCA2_OLAP_S1	ns	0.6681
HAPSTR1-OExp_siBRCA2_OLAP vs. HAPSTR1-OExp_siBRCA2_OLAP_S1	ns	>0.9999
U2OS siBRCA2 vs. U2OS siBRCA2_S1	****	<0.0001
U2OS siBRCA2_S1 vs. U2OS siBRCA2+OLAP_S1	****	<0.0001
U2OS siBRCA2 + OLAP vs. U2OS siBRCA2+OLAP_S1	****	<0.0001

Dunn's multiple comparisons test	Summary	Adjusted P Value
HAPSTR1-OExp_siBRCA2 vs. HAPSTR1-OExp_siBRCA2_S1	****	<0.0001
HAPSTR1-OExp_siBRCA2_S1 vs. HAPSTR1-OExp_siBRCA2_OLAP_S1	****	<0.0001
HAPSTR1-OExp_siBRCA2_OLAP vs. HAPSTR1-OExp_siBRCA2_OLAP_S1	ns	>0.9999
U2OS siBRCA2 vs. U2OS siBRCA2_S1	****	<0.0001
U2OS siBRCA2_S1 vs. U2OS siBRCA2+olap_S1	****	<0.0001
U2OS siBRCA2 + oalp vs. U2OS siBRCA2+olap_S1	****	<0.0001
